# A meta-analysis of animal studies evaluating the effect of hydrogen sulfide on ischemic stroke: is the preclinical evidence sufficient to move forward?

**DOI:** 10.1007/s00210-024-03291-5

**Published:** 2024-07-17

**Authors:** Selda Emre Aydıngöz, Ariyan Teimoori, Halit Güner Orhan, Elif Demirtaş, Nargız Zeynalova

**Affiliations:** Department of Medical Pharmacology, Başkent University Faculty of Medicine, Ankara, Turkey

**Keywords:** Animal models, Hydrogen sulfide, Ischemic stroke, Meta-analysis, Systematic review

## Abstract

Hydrogen sulfide (H_2_S) is a gasotransmitter that has been studied for its potential therapeutic effects, including its role in the pathophysiology and treatment of stroke. This systematic review and meta-analysis aimed to determine the sufficiency of overall preclinical evidence to guide the initiation of clinical stroke trials with H_2_S and provide tailored recommendations for their design. PubMed, Web of Science, Scopus, EMBASE, and MEDLINE were searched for studies evaluating the effect of any H_2_S donor on in vivo animal models of regional ischemic stroke, and 34 publications were identified. Pooling of the effect sizes using the random-effect model revealed that H_2_S decreased the infarct area by 34.5% (95% confidence interval (CI) 28.2–40.8%, *p* < 0.0001), with substantial variability among the studies (*I*^2^ = 89.8%). H_2_S also caused a 37.9% reduction in the neurological deficit score (95% CI 29.0–46.8%, *p* < 0.0001, *I*^2^ = 63.8%) and in the brain water content (3.2%, 95% CI 1.4–4.9%, *p* = 0.0014, *I*^2^ = 94.6%). Overall, the studies had a high risk of bias and low quality of evidence (median quality score 5/15, interquartile range 4–9). The majority of the included studies had a “high” or “unclear” risk of bias, and none of the studies overall had a “low” risk. In conclusion, H_2_S significantly improves structural and functional outcomes in in vivo animal models of ischemic stroke. However, the level of evidence from preclinical studies is not sufficient to proceed to clinical trials due to the low external validity, high risk of bias, and variable design of existing animal studies.

**Supplementary Information:**

The online version contains supplementary material available at 10.1007/s00210-024-03291-5.

## Introduction

Stroke is the second most prevalent cause of death and acquired adult disability worldwide (Saini et al. 2021). Despite a decline in mortality rates, the overall burden of stroke is on the rise.

Approximately 80% of all strokes are ischemic, resulting from the blockage of cerebral arteries (Virani et al. 2020). Ischemic stroke treatment has traditionally focused on reperfusion techniques such as intravenous thrombolysis with tissue plasminogen activator (tPA) and endovascular therapy, which are effective but limited by a narrow therapeutic window and risk of hemorrhage (Powers et al. 2018). Other treatment options include stem cell therapy, which promotes neurogenesis and reduces inflammation; natural products, peptides, and other pharmacological agents which decrease excitotoxicity, oxidative stress, apoptosis, and neuroinflammation; and nanomedicine, which targets drug delivery to the brain to improve outcomes (Lyden 2021; Jia et al. 2024). Although these emerging treatment options showed promising results in animal studies, translational challenges, including low study quality and publication bias in preclinical studies, have hindered their clinical application (Macleod et al. 2008; Sena et al. 2010). Consequently, it is advisable to conduct a well-designed systematic review and meta-analysis of preclinical stroke studies before embarking on clinical trials to provide nonbiased summaries of the available evidence (Bath et al. 2009; Dirnagl and Macleod 2009).

Hydrogen sulfide (H_2_S) has emerged as a candidate agent, and its effectiveness has been evaluated in various experimental animal models of ischemic stroke, yielding conflicting results (Gopalakrishnan et al. 2019; Jia et al. 2019; Ding et al. 2023). H_2_S functions as an endogenously produced gasotransmitter and serves as a vital signaling molecule at low concentrations, playing a significant role in vasodilation and blood pressure regulation (Kimura 2021). Growing evidence suggests that exogenous H_2_S donors protect organs, including the brain, from ischemia-reperfusion injury by mitigating inflammation, oxidative stress, mitochondrial damage, and apoptosis (Gopalakrishnan et al. 2019; Jia et al. 2019; Ding et al. 2023; Emre Aydıngöz et al. 2023). H_2_S therapy offers significant advantages over tPA by reducing hemorrhagic complications for ischemic stroke treatment (Liu et al. 2016; Jia et al. 2019). Combining H_2_S with tPA can improve safety and efficacy, suggesting a promising approach for enhancing stroke management (Liu et al. 2016).

Despite the extensive exploration of the beneficial effects of H_2_S against ischemic stroke in individual animal studies, no clinical trials have verified its efficacy in human settings. Furthermore, although many preclinical studies and narrative reviews have shown that H_2_S may be effective for treating stroke (Dou et al. 2016; Chan and Wong 2017; Gopalakrishnan et al. 2019; Narne et al. 2019; Ding et al. 2023), a systematic review of animal studies and quantification of the available data by meta-analysis have not yet been performed. Given the persistent translational challenges in preclinical stroke studies, a thorough systematic review and meta-analysis of existing evidence on the effectiveness of H_2_S against ischemic stroke is crucial.

This systematic review and meta-analysis aimed to determine the sufficiency of overall preclinical evidence to guide the initiation of clinical stroke trials with H_2_S and provide tailored recommendations for their design.

The research question is “How effective is H_2_S compared with no treatment on structural and functional outcomes in in vivo animal models of ischemic stroke?”

## Methods

### Study protocol overview

This systematic review and meta-analysis was conducted following a registered PROSPERO protocol (CRD42023380938, date: 10/01/2023). No amendments were made to the study protocol afterward. The guidelines of the Preferred Reporting Items for Systematic Reviews and Meta-Analyses (PRISMA) (Moher et al. 2009) and recommendations for the systematic review and meta-analysis of animal studies (Vesterinen et al. 2014; Soliman et al. 2020) were followed. The PRISMA checklist is presented in Supplement 1.

### Literature search

The inclusion criterion was an in vivo animal model of regional ischemic stroke treated with any H_2_S donor. The exclusion criteria included in vitro, ex vivo, and in silico studies; observational studies; experimental studies lacking a control group; reviews; conference presentations; studies on human subjects; investigations involving interventions increasing endogenous H_2_S levels; and studies lacking crucial information for meta-analysis. There were no language or date restrictions.

The PubMed, Web of Science, Scopus, EMBASE, and MEDLINE databases were searched using the specific criteria detailed in Supplement 2. Animal filters devised by van der Mierden et al. (2022) and de Vries et al. (2014) were applied to the PubMed and EMBASE databases. The Polyglot Search Translator (Clark et al. 2020) was utilized to translate the search strategies across databases. The initial search was performed on February 23, 2023, with an update on August 1, 2023. Duplicates were removed, and the title/abstract was prescreened using the SyRF screening application (http://app.syrf.org.uk/) (Bahor et al. 2021). Prescreening and full-text screening were performed by two independent reviewers, with discrepancies resolved through discussion with a third reviewer.

### Data extraction and effect measures

The key study characteristics extracted were experimental group, number of animals per group, species, sex, age and weight of the animals, comorbidity status, anesthetic agent, ischemic model, cotreatment, H_2_S donor, dosage, administration time, and route. For the effect measures, data on infarct volume (primary outcome), neurological deficit score, and brain edema were extracted. Mechanistic outcome measures involving biomarkers are reported in five or more studies. In cases where outcomes were reported at different time points for the same group of animals, the last time point measurement was extracted.

For all outcome measures, published continuous data (mean) and variance (standard deviation/standard error of the mean) were extracted without unit conversion. In cases where the variance was given as the standard error of the mean, the standard deviation was estimated on the basis of the sample size. The data presented graphically were extracted using WebPlotDigitizer (version 4.5, August 2021, Pacifica, California, USA). For one study, the standard deviation for infarct volume was obtained from the author of the original paper (Genc et al. 2023). If it was not possible to obtain the necessary effect measures, the paper was not included in the meta-analysis. Two reviewers independently performed the data extraction using the SyRF application. In cases of < 10% difference between extracted data, an average of the two values was taken; discrepancies > 10% were resolved through discussion with a third reviewer. The extracted raw data are presented in Supplement 3.

### Meta-analysis

A meta-analysis was conducted on the full texts of a predefined number of studies (at least 10) with similar study designs, comparing the effects of any H_2_S donor with no treatment on structural and functional outcomes in in vivo animal models of ischemic stroke. All analyses were performed using the “metafor” package (Viechtbauer 2010) in R 4.2.1 (R Foundation for Statistical Computing, Vienna, Austria; https://www.r-project.org/) and guided by the online version “Doing Meta-Analysis with R: A Hands-On Guide” (Harrer et al. 2021). The R codes are presented in Supplement 4.

#### Pooling of data

The data were pooled for the outcome measures that were reported in six or more studies. Pooling of effect sizes was accomplished through the calculation of the normalized mean difference (NMD) for infarct area, neurological deficit score, and brain edema, considering that the data were on a ratio scale. For other outcome measures, Hedges’ g (bias-corrected standardized mean difference (SMD)) served as the effect size. Random-effect models with a restricted maximum likelihood (RELM) estimate of between-study variance were employed to obtain pooled effect sizes for each outcome. The 95% confidence interval (CI) surrounding the pooled effect was computed using Knapp-Hartung adjustments (Knapp and Hartung 2003). Forest plots were generated to summarize the meta-analysis results. The sensitivity of the analysis was assessed by repeating it with a fixed-effect model and performing the leave-one-out test.

#### Assessment of heterogeneity

The between-study variance was estimated by calculating Tau^2^, *Q* (*χ*^2^), and its significance level. The percentage of residual variation attributable to between-study heterogeneity was expressed as *I*^2^. Meta-regression (subgroup) analysis was conducted to investigate the impact of various study characteristics on the treatment effect, including the type, dose, and application time of H_2_S donors; duration and mode of ischemia (temporary vs. permanent); age and species of animals; outcome assessment time; and risk of bias measures. The number of subgroups was minimized, defined according to the cutoff points identified by a data-driven approach (i.e., median), as suggested by Wang et al. (2021). Meta-regression analysis was performed on the pooled infarct volume data, the outcome measure reported by most of the studies.

#### Assessment of publication bias

Publication bias was assessed using small-study effect methods involving visual inspection of the funnel plot and quantification of asymmetry with Egger’s regression test. The trim-and-fill method was applied to address funnel plot asymmetry and obtain a corrected effect estimate. To avoid false-positive results, NMD was used as the effect size measure in the funnel plot, as suggested by Zwetsloot et al. (2017).

#### Assessment of quality and risk of bias

The quality of evidence for each study was evaluated using the CAMARADES study quality checklist (Sena et al. 2007), modified by McCann et al. (2016), to incorporate relevant items from the updated STAIR criteria (Fisher et al. 2009). The risk of bias was categorized according to the SYRCLE risk of bias tool (Hooijmans et al. 2014), and the risk of bias plot was generated using the web-based application robvis (https://mcguinlu.shinyapps.io/robvis/) (McGuinness and Higgins 2021). Two reviewers independently assessed study quality and risk of bias, with discrepancies resolved through discussion with a third reviewer.

The significance level was set at *p* < 0.05 throughout the analysis.

## Results

### Characteristics of the selected studies

**Fig. 1:**
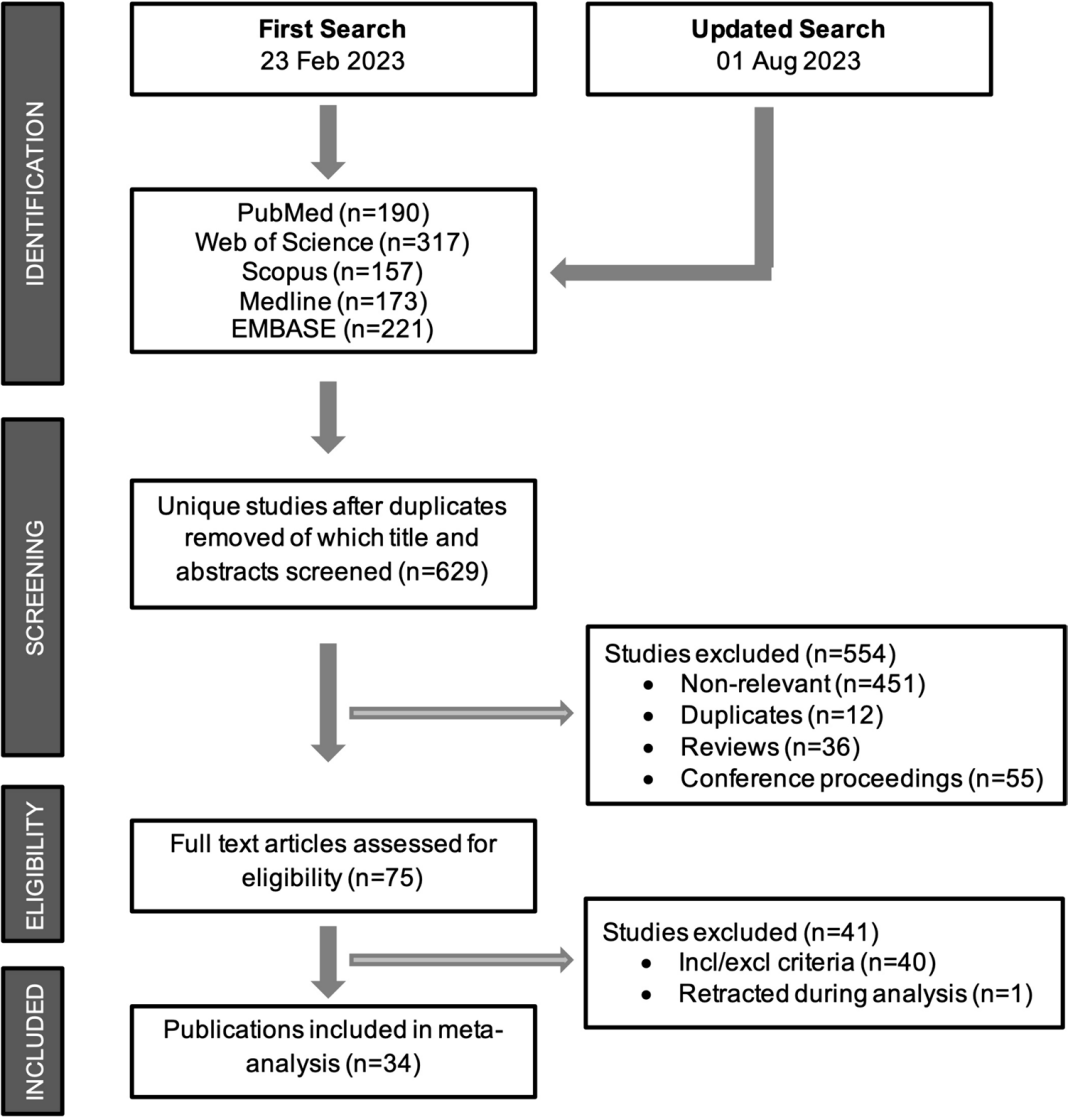
PRISMA flow diagram of the systematic review process

Thirty-four publications meeting the study selection criteria were identified through a systematic review of the literature (Fig. 1). Seven of these studies were conducted in mice, and the remaining 27 were conducted in rats; all of these studies used a middle cerebral artery occlusion model to induce transient stroke. Ischemia was induced for a wide range of durations, from 1 to 24 h. With the exception of three publications where the sex of the animal was not specified, all studies used male animals. Only three studies were carried out on aged animals, and none of the studies used animals with comorbidities. Notably, most studies (26/34) used NaHS as the H_2_S donor, mostly by intraperitoneal administration, with varying doses (1–40 mg/kg) and times of administration (before, during, or after ischemia). The basic characteristics of the included studies and published measures are summarized in Supplement 5.

### Pooled effect measures

#### Infarct area

**Fig. 2:**
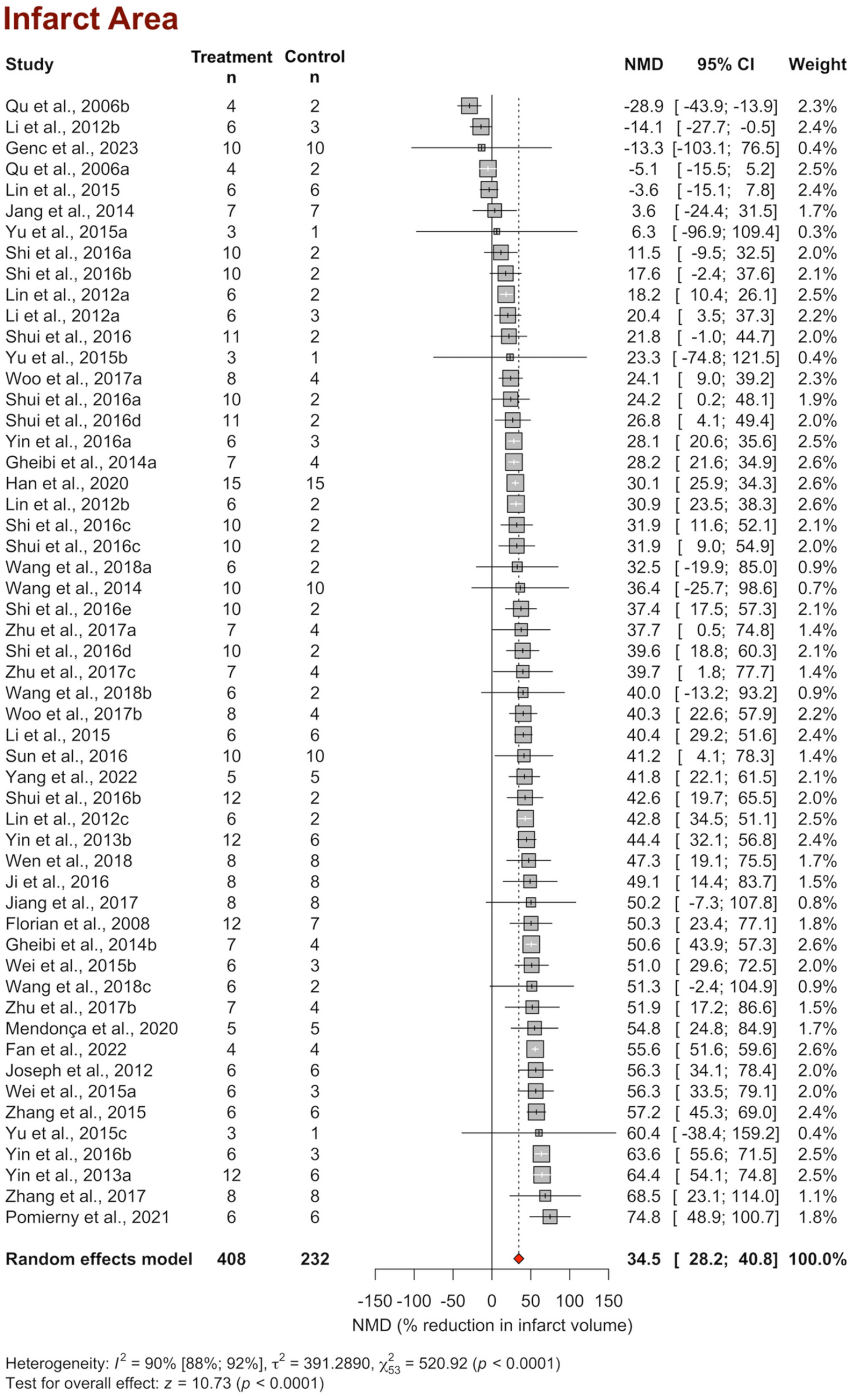
Forest plot of individual and overall effect sizes and heterogeneity statistics for infarct area. NMD, normalized mean difference; CI, confidence interval

The infarct area of 408 H_2_S-treated animals versus 232 control animals from 54 comparisons was reported in 31 studies—mm^3^ in 8 studies and the percentage of brain area in the remaining 23 studies. Pooling the NMDs using a random-effect model revealed that H_2_S decreased the infarct area by 34.5% (95% CI 28.2–40.8%, *p* < 0.0001) (Fig. 2). The fixed-effect model also revealed a large and significant effect of H_2_S (36.9% decrease in the infarct area 95% CI 35.2–38.6%, *p* < 0.01), indicating the sensitivity of the random-effect model to the variability between studies. In the leave-one-out analyses, the pooled estimate ranged between 33.7 and 35.9% when each of the studies was removed from the model and the analysis was repeated, indicating that the results of the meta-analysis were relatively stable and not strongly influenced by any single study (Supplement 6).

Heterogeneity analysis indicated substantial variability among the studies, with a *χ*^2^ (heterogeneity statistics) of 520.9 (df = 53, *p* < 0.0001) and an *I*^2^ of 89.8%, suggesting that approximately 90% of the total variability in effect sizes is due to true differences between studies rather than sampling error (Fig. 2). In the leave-one-out analysis, the iteration of the model by excluding each study caused the heterogeneity to vary between 88.3 and 90.0%, confirming that the sources of variability among studies were not primarily driven by one particular study (Supplement 6).

#### Neurological deficit score

**Fig. 3:**
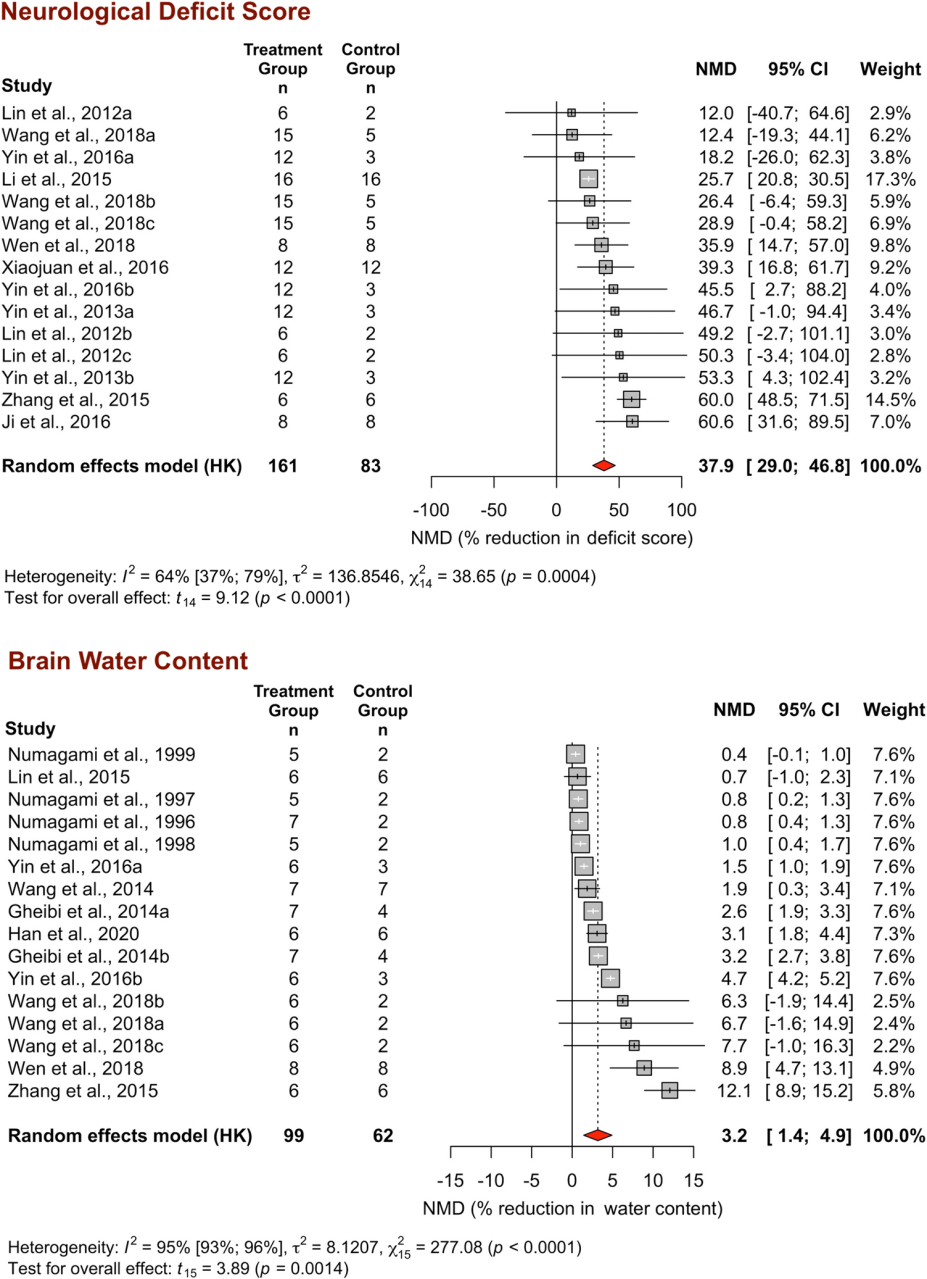
Forest plot of individual and overall effect sizes and heterogeneity statistics for neurological deficit score and brain water content. NMD, normalized mean difference; CI, confidence interval

The neurological deficits of the animals were assessed in 20 studies by various scales, including the Longa, Garcia, mNSS, Bederson, and Philips scoring systems. The most preferred scale among these was the Longa scoring system, which was employed in 161 H_2_S-treated animals versus 83 control animals in 15 comparisons from 9 studies. High scores indicate severe neurological deficits on the five-point (0 to 4) Longa scale (Longa et al., 1989). Overall, H_2_S caused a 37.9% reduction in the Longa score (95% CI 29.0–46.8%, *p* < 0.0001), with moderate variability between studies (*I*^2^ = 63.8%, *χ*^2^ = 38.6, df = 14, *p* = 0.0004) (Fig. 3).

#### Brain water content

The brain water content of 99 H_2_S-treated animals versus 62 control animals was reported in 9 studies as [(ischemic hemisphere wet weight − dry weight)/wet weight] × 100%. Compared to the control group, H_2_S caused a small but significant reduction in brain water content (3.2%, 95% CI 1.4–4.9%, *p* = 0.0014), with very high heterogeneity (*I*^2^ = 94.6%, *χ*^2^ = 277.1, df = 15, *p* < 0.0001) (Fig. 3).

#### Biochemical markers for apoptosis and inflammation

**Fig. 4:**
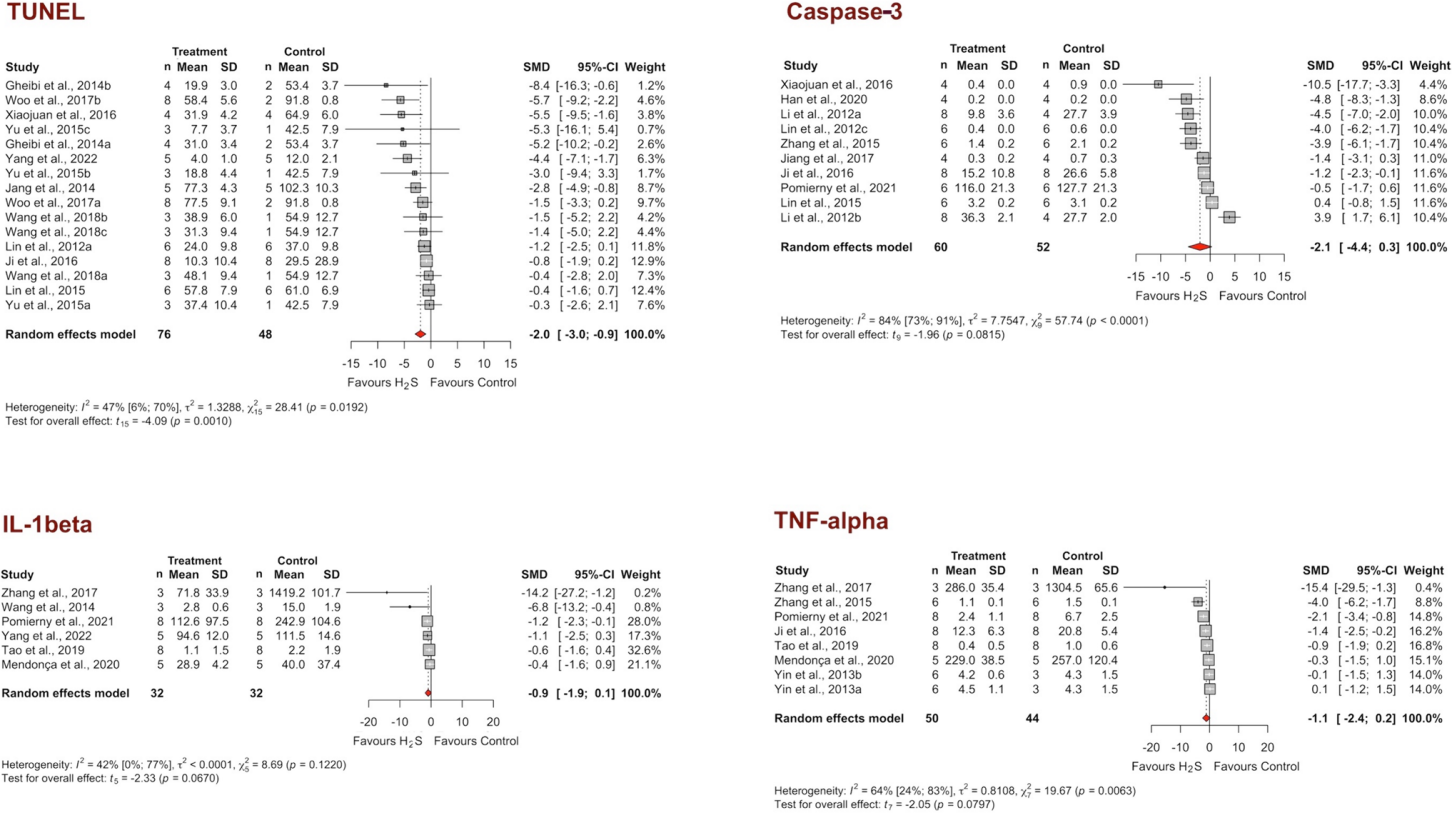
Forest plots of individual and overall effect sizes and heterogeneity statistics for biomarkers of apoptosis (TUNEL and caspase-3) and proinflammatory cytokines (TNF-alpha and IL-1beta). SMD, standardized mean difference; CI, confidence interval

The levels of TUNEL and caspase-3, which are markers of apoptosis, and TNF-alpha and IL-1beta, which are proinflammatory cytokines, were reported in at least six studies. When the data were pooled using the random-effect model, H_2_S significantly decreased only TUNEL (Hedges’ g −2.0, 95% CI −3.0; −0.9, *p* = 0.0010) but had no significant effect on the remaining variables (Hedges’ g −2.1 [−4.4; 0.3], *p* = 0.0815 for caspase-3; −0.9 [−1.9; 0.1], *p* = 0.0670 for IL-1beta; −1.1 [−2.4; 0.2], *p* = 0.0797 for TNF-alpha) (Fig. 4). This finding may suggest that the inhibitory effect of H_2_S on caspase-independent apoptotic pathways or the early/intermediate stage of apoptosis, rather than its anti-inflammatory activity, plays a role in its beneficial effect on the experimental stroke model.

### Subgroup analysis

The results of subgroup analyses are reported in Table 1, which shows the estimated effect in each subgroup, the *p* value of the test for subgroup differences, residual heterogeneity, and the amount of heterogeneity accounting for that study variable. As shown in Table 1, animal species; blinded outcome assessment; controlling temperature; the STAIR score of the study; duration of ischemia; measurement unit for infarct area, type, dose, and application time of H_2_S; and time of outcome measure assessment did not have a significant effect on the observed heterogeneity between studies. However, although the difference was slightly greater than the conventional threshold (*p* = 0.0551), it is remarkable that H_2_S donors other than NaHS induced a much greater reduction in infarct volume than did NaHS donors (40.0% vs. 27.9%). The pooled effect of each H_2_S donor is shown in a forest plot in Supplement 7. An unexpected finding in the subgroup analysis was that randomization significantly increased the effect size (*p* = 0.01). Unexpectedly, H_2_S was found to cause a significantly greater reduction in the infarct area in randomized experiments, accounting for 13.3% of the heterogeneity.

**Table 1:** The results of subgroup analyses

Subgroups	Number of comparisons	NMD (% reduction in infarct volume) [95% CI]	*p* value for subgroup comparison	*I*^2^ (residual heterogeneity)	*R*^2^ (amount of heterogeneity accounted for)
Species					
Rat	42	33.8 [26.8; 40.9]	0.6484	90.6%	0.0%
Mouse	12	37.6 [22.9; 52.3]			
Random allocation to groups					
Yes	36	40.5 [38.6; 42.4]	0.0100	88.6%	13.3%
Unknown	18	25.2 [21.8; 28.7]			
Blinded outcome assessment					
Yes	28	39.3 [30.2; 48.5]	0.1582	89.9%	1.8%
Unknown	26	36.3 [34.4; 38.2]			
Temperature controlled					
Yes	42	32.3 [30.3; 34.2]	0.7933	89.6%	0.0%
Unknown	12	48.3 [45.2; 51.4]			
CAMARADES-STAIR score (0–15)*					
< 5	15	30.5 [27.8; 33.1]	0.1671	89.5%	3.6%
5–9	32	40.7 [38.5; 42.9]			
> 9	7	43.1 [34.7; 51.4]			
Duration of ischemia					
Short	29	38.3 [28.9; 47.6]	0.2807	89.7%	0.0%
Long	25	31.3 [36.5; 40.8]			
Infarct area measurement unit					
%	45	35.5 [28.6; 42.4]	0.5038	90.0%	0.0%
mm^3^	9	29.6 [22.8; 39.9]			
H_2_S type					
NaHS	25	27.9 [18.8; 37.0]	0.0551	89.4%	5.9%
Other	29	40.0 [31.7; 48.4]			
H_2_S dose**					
High	15	25.9 [13.9; 37.9]	0.5806	85.0%	0.0%
Low	16	30.7 [13.6; 47.8]			
H_2_S application time					
Before ischemia	19	32.8 [22.5; 43.2]	0.9163	90.0%	0.0%
During ischemia	25	35.4 [26.2; 44.7]			
During reperfusion	10	36.2 [19.1; 53.3]			
Time of outcome assessment					
> 24 h after stroke	10	39.3 [21.4; 57.2]	0.5726	90.5%	0.0%
24 h after stroke	44	33.8 [27.1; 40.6]			

*CAMARADES study quality checklist modified to include relevant items from the updated Stroke Therapy Academic Industry Roundtable (STAIR) criteria

**Only studies that used NaHS as an H_2_S donor were included in the subgroup analysis for H_2_S dose

### Publication bias

**Fig. 5:**
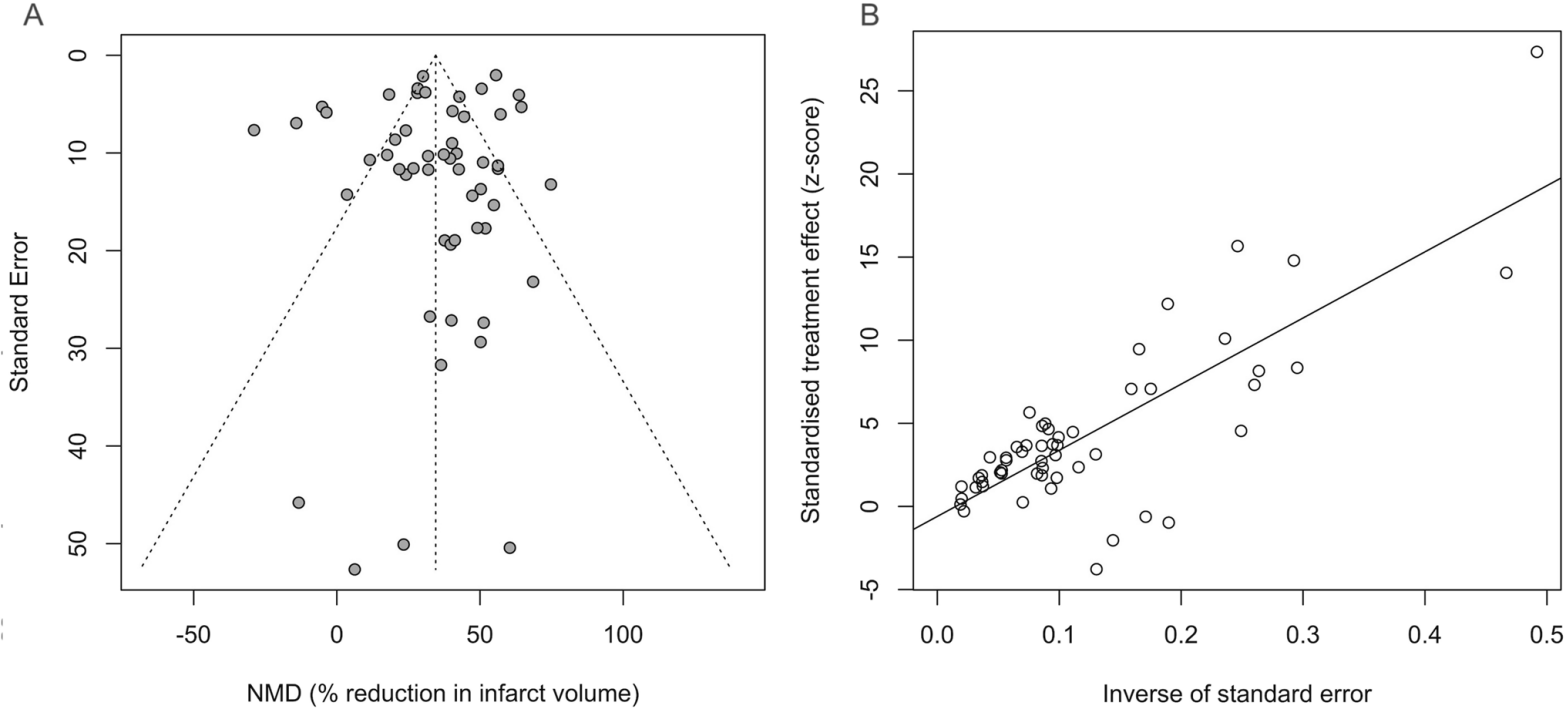
**A** Funnel plot representing the relationship between effect size and standard error (precision) for the studies included in the meta-analysis. The y-axis displays the standard error of the effect size, inversely proportional to study precision, while the x-axis represents the effect size estimate, which is the normalized mean difference (NMD) as a percent reduction in infarct volume. Each point on the plot corresponds to an individual study included in the meta-analysis. The diagonal line in the center represents the overall effect estimate. **B** A scatter plot of effect size against precision with the Egger regression line overlaid (*t* = −0.89, df = 52, *p* = 0.3784)

The visual inspection of the funnel plot and Egger’s regression test (*t* = −0.89, df = 52, *p* = 0.3784) revealed no evidence of funnel plot asymmetry (Fig. 5). Parallel to this finding, the trim-and-fill method revealed no missing studies and did not adjust the model.

### Study quality and risk of bias

**Fig. 6:**
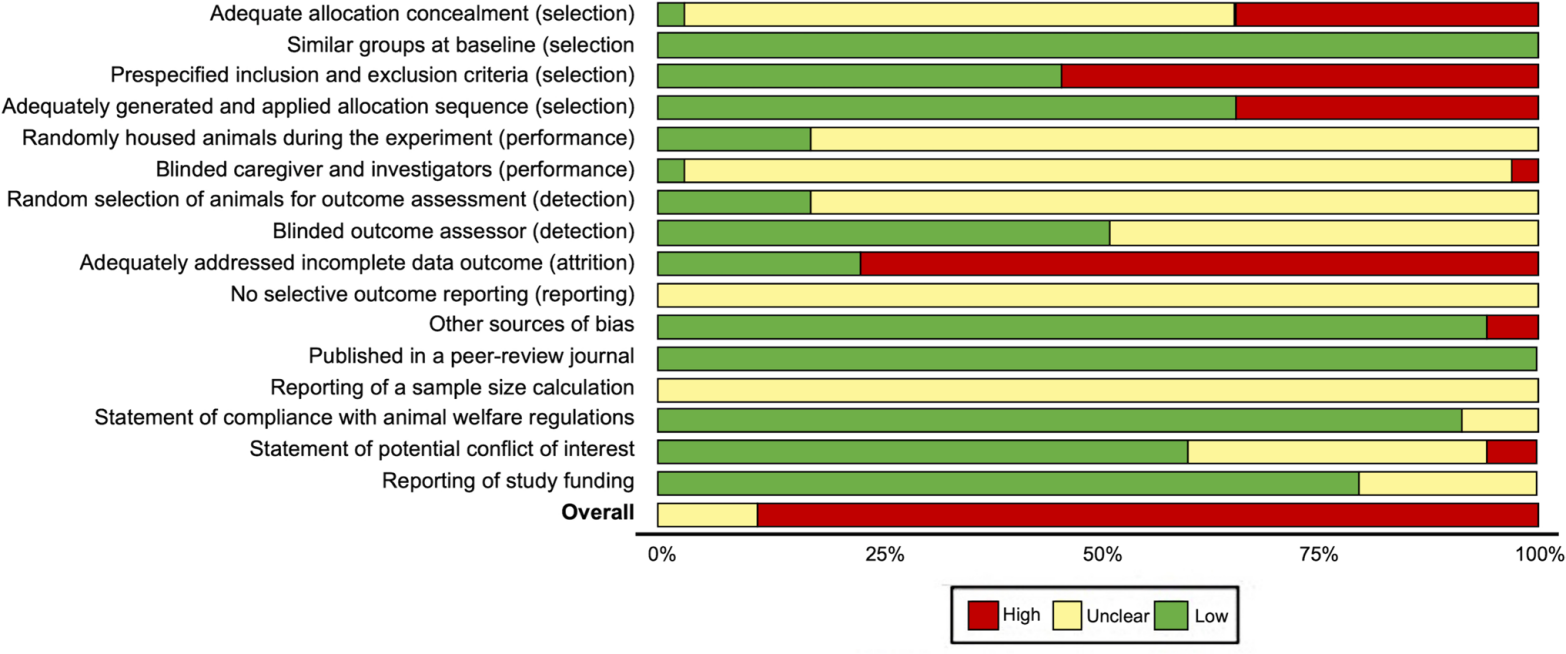
Weighted bar plots of the distribution of risk of bias judgments within each bias domain of the SYRCLE risk of bias tool for all of the included studies (*n* = 34)

According to the CAMARADES study quality checklist modified to include relevant items from the updated STAIR criteria, studies had low quality and high risk of bias (median score 5/15, interquartile range 4–9). Randomization of group allocation, blinded induction of ischemia, and blinded assessment of outcome—factors whose absence created the highest risk of bias—were reported in only 65%, 3%, and 50% of the studies, respectively (Table 2). Categorization of risk of bias by using the SYRCLE risk of bias tool revealed that the majority of the included studies had a “high” or “unclear” risk of bias, and none of the studies overall had a “low” risk (Fig. 6).

**Table 2:** The risk of bias score of each publication according to the CAMARADES study quality checklist was modified to include relevant items from the updated Stroke Therapy Academic Industry Roundtable (STAIR) criteria (Sena et al. 2007; Fisher et al. 2009; McCann et al. 2016)

	Checklist items*															Total score
	1	2	3	4	5	6	7	8	9	10	11	12	13	14	15	
Fan et al. (2022)	✓		✓						✓	✓				✓		5
Florian et al. (2008)	✓	✓			✓				✓							4
Genc et al. (2023)	✓	✓	✓		✓				✓	✓		✓	✓	✓		9
Gheibi et al. (2014)	✓	✓							✓							3
Han et al. (2020)	✓	✓	✓						✓	✓		✓		✓	✓	8
Jang et al. (2014)	✓				✓				✓	✓				✓		5
Ji et al. (2016)	✓	✓	✓		✓				✓	✓		✓	✓	✓	✓	10
Jiang et al. (2017)	✓	✓			✓				✓					✓		5
Joseph et al. (2012)	✓	✓	✓						✓	✓	✓	✓	✓		✓	9
Li et al. (2012)	✓		✓						✓					✓		4
Li et al. (2015)	✓		✓						✓	✓			✓	✓		6
Li et al. (2016)	✓		✓						✓	✓				✓		5
Lin et al. (2012)	✓	✓			✓				✓							4
Lin et al. (2015)	✓	✓	✓						✓					✓		5
Mendonça et al. (2020)	✓	✓	✓		✓				✓			✓	✓	✓		8
Numagami et al. (1996)	✓	✓												✓		3
Pomierny et al. (2021)	✓	✓	✓	✓	✓				✓	✓		✓		✓	✓	10
Qu et al. (2006)	✓	✓							✓					✓		4
Shi et al. (2016)	✓	✓							✓	✓	✓	✓		✓	✓	8
Shui et al. (2016)	✓	✓	✓		✓				✓	✓		✓		✓	✓	9
Sun et al. (2016)	✓		✓						✓					✓		4
Tao et al. (2019)	✓	✓							✓	✓				✓		5
Wang et al. (2014)	✓	✓	✓		✓				✓	✓		✓	✓	✓	✓	10
Wang et al. (2018)	✓		✓		✓				✓	✓		✓	✓	✓	✓	9
Wei et al. (2015)	✓	✓	✓		✓				✓	✓	✓	✓		✓	✓	10
Wen et al. (2018)	✓								✓	✓				✓		4
Woo et al. (2017)	✓	✓	✓		✓				✓	✓	✓	✓	✓	✓	✓	11
Yang et al. (2022)	✓								✓	✓				✓		4
Yin et al. (2013)	✓		✓									✓	✓			4
Yin et al. (2016)	✓	✓	✓		✓									✓		5
Yu et al. (2015)	✓	✓							✓	✓		✓		✓	✓	7
Zhang et al. (2015)	✓	✓	✓		✓				✓							5
Zhang et al. (2017)	✓	✓	✓		✓				✓			✓	✓	✓	✓	9
Zhu et al. (2017)	✓	✓	✓		✓				✓	✓		✓		✓	✓	9
Number of reporting studies	34	23	22	1	17	0	0	0	31	20	4	15	9	27	12	Mean = 5 IQR = 4–9
Percentage	100%	68%	65%	3%	50%	0%	0%	0%	91%	59%	12%	44%	26%	79%	35%	

*1, peer-reviewed publication; 2, control of temperature; 3, randomization of group allocation; 4, blinded induction of ischemia; 5, blinded assessment of outcome; 6, avoidance of anesthetics with intrinsic neuroprotective properties; 7, use of animals with comorbidities (e.g., hypertension, diabetes); 8, sample size calculation; 9, statement of compliance with animal welfare requirements; 10, statement of potential conflicts of interest; 11, physiological monitoring during stroke induction; 12, prespecified inclusion and exclusion criteria; 13, reporting of animals excluded from analysis; 14, reporting of study funding; 15, injury confirmed via laser Doppler or perfusion imaging

## Discussion

To our knowledge, this is the first meta-analysis of preclinical studies evaluating the effect of H_2_S in an experimental stroke model. The analysis revealed that compared with no treatment, H_2_S significantly improved structural and functional outcomes in in vivo animal models of ischemic stroke. However, when considered as a whole, the level of evidence from preclinical studies is not sufficient to proceed to clinical trials due to the low external validity, high risk of bias, and variable design of existing animal studies.

### Neuroprotective effects of H_2_S

Among the studies included in the meta-analysis, infarct volume was reported in 31 (91.2%), whereas neurological deficit scores were reported in only 20 (58.8%). Although these percentages are similar to those found in preclinical stroke studies in the literature, nearly all clinical stroke trials have reported functional outcome as the primary endpoint, and fewer than 15% of clinical trials have reported infarct volume (Schmidt-Pogoda et al. 2020). This factor may reduce the clinical translation of animal studies evaluating the effect of H_2_S on stroke. A meta-analysis of these studies indicated that H_2_S is very effective against stroke, decreasing the infarct area by 34.5% (95% CI 28.2–40.8%, *p* < 0.0001) and improving neurological deficit scores by 37.9% (95% CI 29.0–46.8%, *p* < 0.0001). This finding is in line with the findings of other potential agents against stroke, which decrease infarct volume and improve functional outcome by 24% on average in experimental studies (Sena et al. 2007; McCann et al. 2016; Schmidt-Pogoda et al. 2020). Due to the high risk of bias and low quality of evidence, which are common problems in preclinical studies, the efficacy of stroke treatments has declined from experimental to clinical trials (Macleod et al. 2004; Sena et al. 2007; McCann et al. 2016; Schmidt-Pogoda et al. 2020). Given the high risk of bias of the studies included in our meta-analyses, H_2_S is also likely to be less effective in clinical studies than in preclinical studies. Furthermore, functional outcome, the primary endpoint for clinical trials, has been evaluated in preclinical H_2_S stroke studies by using different methods, a situation that further jeopardizes the translation of animal studies. Twenty studies evaluating neurological deficits utilized various functional scales, including the Longa, Garcia, mNSS, Bederson, and Philips scoring systems, each covering different aspects of neurological function. Since Longa was the most commonly used scale, we performed pooled analyses including only studies using the Longa scale to increase the validity of the meta-analysis. Although this approach reduces between-study heterogeneity, it limits the power of the analysis due to the inclusion of only nine studies.

Another piece of evidence for the neuroprotective effect of H_2_S was a reduction in the brain water content, which was reported in nine studies using the wet-dry weight method. Brain water content is a measure commonly used in experimental animal stroke studies to assess cerebral edema, which occurs as a result of ischemia-induced inflammation and breakdown of the blood-brain barrier (Kozler et al. 2022). In the context of stroke research, cerebral edema is a significant concern because it can contribute to secondary damage and worsen outcomes following a stroke (Chen et al. 2021). H_2_S caused a small but significant reduction in brain water content (3.2%, 95% CI 1.4–4.9%, *p* = 0.0014) when the findings of the nine studies were pooled. As noted by Keep et al. (2012), when the brain water content is calculated in % using the wet-dry weight method, a “small” change in the % water content reflects a large change in the swelling of the tissue. Therefore, keeping in mind the high between-study heterogeneity (*I*^2^ = 94.6%, *p* < 0.0001), H_2_S significantly reduced brain edema after stroke.

### Mechanisms of the neuroprotective effects of H_2_S

**Fig. 7:**
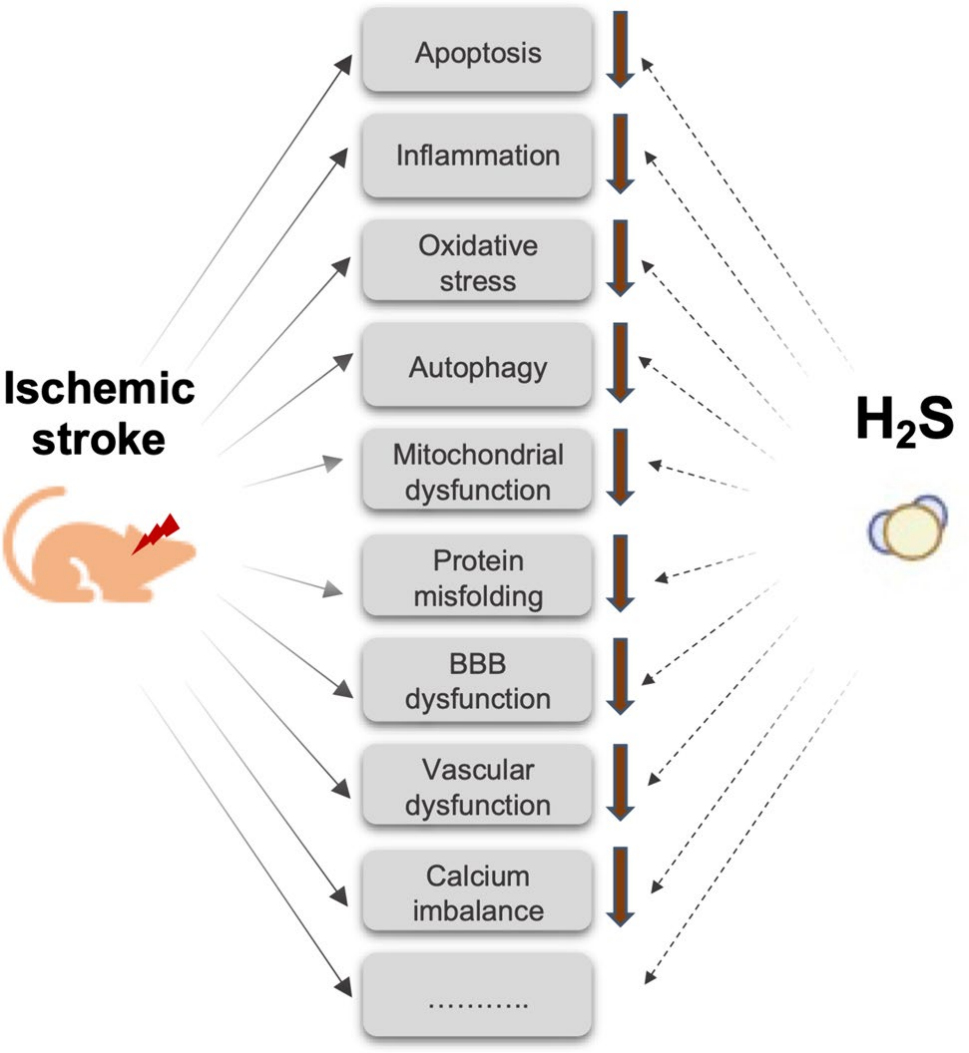
Proposed mechanisms mediating the neuroprotective effect of H_2_S in cerebral ischemic stroke

Research in recent decades has suggested that exogenous H_2_S and H_2_S donors exert neuroprotective effects on animal stroke models by inhibiting apoptosis, inflammation, oxidative stress, mitochondrial dysfunction, blood-brain barrier leakage, and disrupted cerebrovascular homeostasis, all of which contribute to ischemia and reperfusion injury in the brain (Dou et al. 2016; Chan and Wong 2017; Gopalakrishnan et al. 2019; Narne et al. 2019; Ding et al. 2023) (Fig. 7). The studies included in the meta-analysis evaluated these pathways by measuring various biomarkers. Among these, the ones for which the effect sizes could be pooled were the apoptosis markers TUNEL and caspase-3 and the inflammation markers IL-1beta and TNF-alpha. TUNEL was the only biomarker for which H_2_S significantly reduced the pooled results; caspase-3, IL-1beta, and TNF-alpha were not significantly affected. The fact that H_2_S does not affect proinflammatory cytokines but reduces TUNEL staining suggests that its antiapoptotic effect is more important for its neuroprotective effect than its anti-inflammatory effect. The observation that H_2_S decreases the number of TUNEL-positive cells without altering caspase-3 expression has multiple probable interpretations (Didenko et al. 2002). The antiapoptotic effect of H_2_S may involve caspase-independent apoptotic pathways or an early/intermediate stage of apoptosis. Since TUNEL staining could also be associated with other forms of cell death, such as necrosis or autophagy, the effect of H_2_S on TUNEL may also be explained by its inhibitory effect on autophagy induced by stroke (Chen et al. 2014).

### Source of heterogeneity

To identify the sources of high heterogeneity between studies, we performed subgroup analyses to assess the effect of parameters related to the stroke model (e.g., duration of ischemia), treatment (e.g., type, dose, and time of H_2_S administration), and time of outcome assessment on the overall treatment effect. None of these parameters had a significant impact on heterogeneity. However, although not at a statistically significant level, H_2_S donors other than NaHS tended to be more effective in the stroke model. Because many different H_2_S donors were used in the studies, we were unable to obtain a sufficient number of studies for each donor to draw conclusions on the best donor effective against stroke. However, we observed that dual nitric oxide and H_2_S donors, such as 8e and 8d (Yin et al. 2016), and slow H_2_S-releasing agents, such as anethole dithiolethione (ADT) (Powell et al. 2018), are more effective than NaHS in preclinical stroke models. In further animal studies on stroke, we suggest using these or similar H_2_S donors.

### Study quality and risk of bias

Compared with the meta-analyses of preclinical stroke studies in the literature, the quality of the studies included in our model was low (García-Bonilla et al. 2012; Schmidt et al. 2014; McCann et al. 2016; Boboc et al. 2023; Li et al. 2023). In previous studies, the median quality scores ranged between 4 and 7 out of 10 and between 6 and 11 out of 15, whereas the median score for the 34 studies in our analysis was 5 out of 15. Considering that most of the studies were published in 2015 and later, it is surprising that despite all efforts, preclinical stroke studies are still far from the expected quality. Low experimental and reporting quality creates a high risk of bias, making the reliability of the findings questionable. The most critical components for bias in stroke experiments (Sena et al. 2007), which are randomization of group allocation and blinded assessment of outcome, were reported in only 65% and 50% of the studies, respectively. Additionally, none of the studies reported any concern about avoiding the intrinsic neuroprotective effects of anesthesia. Five studies did not specify the anesthetics used. Chloral hydrate (*n* = 14), isoflurane (*n* = 12), halothane (*n* = 1), ketamine (*n* = 1), and sodium pentobarbital (*n* = 1) were also used. Although the CAMARADES checklist emphasizes that ketamine is the only anesthetic to avoid its neuroprotective effect (Sena et al. 2007), as there is evidence in the literature for neuroprotective effects of other anesthetics as well (Carbone and Austin 2016), we considered all studies to have a high risk of bias for this item. The lack of sample size calculations is another factor that increases the risk of bias. None of the studies reported any calculation or explanation for the sample size, which can lead to several drawbacks and limitations in the study design and interpretation of results, such as insufficient statistical power, inaccurate effect size estimates, increased type 1 and 2 errors, limited generalizability of the study findings, and increased ethical considerations (Percie du Sert et al. 2020). The use of male animals only, no use of animals with comorbidities, and no cotreatment were other factors that reduced the clinical and translational validity of the studies included in the meta-analysis.

In the subgroup analysis in which we evaluated the effect of the study quality score and risk of bias parameters on heterogeneity, surprisingly, studies reporting randomization and blinding and those with high-quality scores had larger effect sizes, accounting for 13.3%, 1.8%, and 3.6% of the heterogeneity, respectively. Although the *p* value for only randomization is significant according to the conventional significance threshold, others still indicate a difference at the trend level. The observation that randomization and blinding increase the effect size in an experimental animal study may seem counterintuitive and raise questions about the validity of the included studies. Considering that randomization and blinding correct the impact of sampling variability, selection bias, systemic errors, and confounding variables, we may also suggest that the effect of H_2_S on stroke is even greater than the estimates (McGough and Faraone 2009). However, given the poor study quality and small sample size, it is more plausible to suggest that randomization might result in seemingly large effects due to the limited number of subjects and insufficient study quality.

### Publication bias

Another unexpected finding of the present meta-analysis was the symmetric funnel plot and nonsignificant Egger’s regression test, while the studies included in the model had a high risk of bias. This finding may suggest that publication bias may not be a major concern in our meta-analysis. However, it is crucial to note that funnel plot asymmetry is not solely indicative of publication bias; other factors can also contribute to asymmetry (Page et al. 2021). In our case, funnel plot symmetry may reflect genuine heterogeneity in the effect sizes across studies. A high risk of bias in individual studies may have contributed to this heterogeneity. In the absence of publication bias, heterogeneity in effect sizes may arise from variations in study design, animal models, interventions, or other biological factors. It is also possible that if a high risk of bias within individual studies is evenly distributed across studies and not selectively reported, it may not lead to funnel plot asymmetry.

### Limitations

The main limitation of this meta-analysis is the high between-study heterogeneity, which decreases the power and reliability of the pooled effect sizes, making it challenging to detect a true effect of H_2_S in stroke patients. Subgroup analysis and risk of bias assessment revealed that the possible sources of heterogeneity were the various H_2_S donors used in the studies and the low quality of evidence. Although the pooled neuroprotective effect of H_2_S against stroke seems large, the true effect size needs to be confirmed in further well-designed experimental studies.

## Conclusions and recommendations for further studies

H_2_S significantly reduces the stroke-induced infarct area, brain water content, and neurological deficit, primarily through early antiapoptotic effects. These promising findings are limited by the low quality of evidence and the high risk of bias represented by high heterogeneity in the effect sizes across studies. Despite all the efforts to standardize preclinical stroke studies and to increase translational success (Sena et al. 2007; Hooijmans et al. 2014; Schmidt-Pogoda et al. 2020), it is noteworthy that the internal and external validity of the animal studies evaluating the effect of H_2_S in stroke is low. When the studies included in our analysis are considered in the light of the CAMARADES, STAIR, and IMPROVE guidelines (Sena et al. 2007; Fisher et al. 2009; Percie du Sert et al. 2017), our specific recommendations for the future preclinical stroke studies on H_2_S are as follows:

- Use a valid ischemic stroke model
- Increase translation of findings by including both sexes, different age groups, and animals with comorbidities
- Perform an accurate sample size calculation before the experiments
- Report animal loss with reasons
- Randomly allocate animals to the experimental groups
- Blindly assess the outcome
- Evaluate functional outcome, a more clinically relevant endpoint than stroke volume
- Evaluate long-term effect of H_2_S
- Compare H_2_S with current or emerging treatment options for ischemic stroke
- Report transparently

In conclusion, although H_2_S seems promising against stroke, the available preclinical evidence is far from providing a basis for further clinical studies. Preclinical studies that provide high-quality evidence on the effect of H_2_S in stroke patients are still needed.

## Supplementary information

ESM 1 Supplement 1. The PRISMA checklist (DOCX 31 kb)

ESM 2 Supplement 2. Search criteria (DOCX 42 kb)

ESM 3 Supplement 3. Extracted data (XLSX 153 kb)

ESM 4 Supplement 4. R codes (PDF 143 kb)

ESM 5 Supplement 5. Summary of studies (DOCX 3.75 mb)

ESM 6 Supplement 6. Leave-one-out analysis results (DOCX 16 kb)

ESM 7 Supplement 7. Forest plot showing the effect of various H_2_S donors on the infarct area (JPG 1117 kb)

## Data Availability

All the data are available within each publication and in supplementary files.
