# Supplementary material for: A meta-analysis of animal studies evaluating the effect of hydrogen sulfide on ischemic stroke: is the preclinical evidence sufficient to move forward?
Source: Naunyn Schmiedebergs Arch Pharmacol. 2024 Jul 17;397(12):9533–48. doi: 10.1007/s00210-024-03291-5 (PMC11582254; doi:10.1007/s00210-024-03291-5)
Supplement: Supplementary file 2 — Supplement 2. Search criteria (DOCX 42 kb) [file 210_2024_3291_MOESM2_ESM.docx]

**Supplement 1. PubMed / MEDLINE / EMBASE / Web of Science Core Collection / Scopus Search Criteria**

**1. PubMed Search Criteria**

**Concept 1: Population – Animal Filter Pubmed (van der Mierden et al. 2022)**

("animal experimentation"[Mesh] OR "models, animal"[Mesh] OR invertebrates[Mesh] OR Animals[Mesh:noexp] OR "animal population groups"[Mesh] OR chordata[Mesh:noexp] OR "chordata, nonvertebrate"[Mesh] OR vertebrates[Mesh:noexp] OR amphibians[Mesh] OR birds[Mesh] OR fishes[Mesh] OR reptiles[Mesh] OR mammals[Mesh:noexp] OR primates[Mesh:noexp] OR artiodactyla[Mesh] OR carnivora[Mesh] OR cetacea[Mesh] OR chiroptera[Mesh] OR elephants[Mesh] OR hyraxes[Mesh] OR insectivora[Mesh] OR lagomorpha[Mesh] OR marsupialia[Mesh] OR monotremata[Mesh] OR perissodactyla[Mesh] OR rodentia[Mesh] OR scandentia[Mesh] OR sirenia[Mesh] OR haplorhini[Mesh:noexp] OR strepsirhini[Mesh] OR platyrrhini[Mesh] OR tarsii[Mesh] OR catarrhini[Mesh:noexp] OR cercopithecidae[Mesh] OR hylobatidae[Mesh] OR hominidae[Mesh:noexp] OR "gorilla gorilla"[Mesh] OR "pan paniscus"[Mesh] OR "pan troglodytes"[Mesh] OR "pongo pygmaeus"[Mesh]) OR ((animals[tiab] OR animal[tiab] OR mice[Tiab] OR mus[Tiab] OR mouse[Tiab] OR murine[Tiab] OR woodmouse[tiab] OR rats[Tiab] OR rat[Tiab] OR murinae[Tiab] OR muridae[Tiab] OR cottonrat[tiab] OR cottonrats[tiab] OR hamster[tiab] OR hamsters[tiab] OR cricetinae[tiab] OR rodentia[Tiab] OR rodent[Tiab] OR rodents[Tiab] OR pigs[Tiab] OR pig[Tiab] OR swine[tiab] OR swines[tiab] OR piglets[tiab] OR piglet[tiab] OR boar[tiab] OR boars[tiab] OR "sus scrofa"[tiab] OR ferrets[tiab] OR ferret[tiab] OR polecat[tiab] OR polecats[tiab] OR "mustela putorius"[tiab] OR "guinea pigs"[Tiab] OR "guinea pig"[Tiab] OR cavia[Tiab] OR callithrix[Tiab] OR marmoset[Tiab] OR marmosets[Tiab] OR cebuella[Tiab] OR hapale[Tiab] OR octodon[Tiab] OR chinchilla[Tiab] OR chinchillas[Tiab] OR gerbillinae[Tiab] OR gerbil[Tiab] OR gerbils[Tiab] OR jird[Tiab] OR jirds[Tiab] OR merione[Tiab] OR meriones[Tiab] OR rabbits[Tiab] OR rabbit[Tiab] OR hares[Tiab] OR hare[Tiab] OR diptera[Tiab] OR flies[Tiab] OR fly[Tiab] OR dipteral[Tiab] OR drosphila[Tiab] OR drosophilidae[Tiab] OR cats[Tiab] OR cat[Tiab] OR carus[Tiab] OR felis[Tiab] OR nematoda[Tiab] OR nematode[Tiab] OR nematoda[Tiab] OR nematode[Tiab] OR nematodes[Tiab] OR sipunculida[Tiab] OR dogs[Tiab] OR dog[Tiab] OR canine[Tiab] OR canines[Tiab] OR canis[Tiab] OR sheep[Tiab] OR sheeps[Tiab] OR mouflon[Tiab] OR mouflons[Tiab] OR ovis[Tiab] OR goats[Tiab] OR goat[Tiab] OR capra[Tiab] OR capras[Tiab] OR rupicapra[Tiab] OR chamois[Tiab] OR haplorhini[Tiab] OR monkey[Tiab] OR monkeys[Tiab] OR anthropoidea[Tiab] OR anthropoids[Tiab] OR saguinus[Tiab] OR tamarin[Tiab] OR tamarins[Tiab] OR leontopithecus[Tiab] OR hominidae[Tiab] OR ape[Tiab] OR apes[Tiab] OR pan[Tiab] OR paniscus[Tiab] OR "pan paniscus"[Tiab] OR bonobo[Tiab] OR bonobos[Tiab] OR troglodytes[Tiab] OR "pan troglodytes"[Tiab] OR gibbon[Tiab] OR gibbons[Tiab] OR siamang[Tiab] OR siamangs[Tiab] OR nomascus[Tiab] OR symphalangus[Tiab] OR chimpanzee[Tiab] OR chimpanzees[Tiab] OR prosimians[Tiab] OR "bush baby"[Tiab] OR prosimian[Tiab] OR bush babies[Tiab] OR galagos[Tiab] OR galago[Tiab] OR pongidae[Tiab] OR gorilla[Tiab] OR gorillas[Tiab] OR pongo[Tiab] OR pygmaeus[Tiab] OR "pongo pygmaeus"[Tiab] OR orangutans[Tiab] OR pygmaeus[Tiab] OR lemur[Tiab] OR lemurs[Tiab] OR lemuridae[Tiab] OR horse[Tiab] OR horses[Tiab] OR pongo[Tiab] OR equus[Tiab] OR cow[Tiab] OR calf[Tiab] OR bull[Tiab] OR chicken[Tiab] OR chickens[Tiab] OR gallus[Tiab] OR quail[Tiab] OR bird[Tiab] OR birds[Tiab] OR quails[Tiab] OR poultry[Tiab] OR poultries[Tiab] OR fowl[Tiab] OR fowls[Tiab] OR reptile[Tiab] OR reptilia[Tiab] OR reptiles[Tiab] OR snakes[Tiab] OR snake[Tiab] OR lizard[Tiab] OR lizards[Tiab] OR alligator[Tiab] OR alligators[Tiab] OR crocodile[Tiab] OR crocodiles[Tiab] OR turtle[Tiab] OR turtles[Tiab] OR amphibian[Tiab] OR amphibians[Tiab] OR amphibia[Tiab] OR frog[Tiab] OR frogs[Tiab] OR bombina[Tiab] OR salientia[Tiab] OR toad[Tiab] OR toads[Tiab] OR "epidalea calamita"[Tiab] OR salamander[Tiab] OR salamanders[Tiab] OR eel[Tiab] OR eels[Tiab] OR fish[Tiab] OR fishes[Tiab] OR pisces[Tiab] OR catfish[Tiab] OR catfishes[Tiab] OR siluriformes[Tiab] OR arius[Tiab] OR heteropneustes[Tiab] OR sheatfish[Tiab] OR perch[Tiab] OR perches[Tiab] OR percidae[Tiab] OR perca[Tiab] OR trout[Tiab] OR trouts[Tiab] OR char[Tiab] OR chars[Tiab] OR salvelinus[Tiab] OR "fathead minnow"[Tiab] OR minnow[Tiab] OR cyprinidae[Tiab] OR carps[Tiab] OR carp[Tiab] OR zebrafish[Tiab] OR zebrafishes[Tiab] OR goldfish[Tiab] OR goldfishes[Tiab] OR guppy[Tiab] OR guppies[Tiab] OR chub[Tiab] OR chubs[Tiab] OR tinca[Tiab] OR barbels[Tiab] OR barbus[Tiab] OR pimephales[Tiab] OR promelas[Tiab] OR "poecilia reticulata"[Tiab] OR mullet[Tiab] OR mullets[Tiab] OR seahorse[Tiab] OR seahorses[Tiab] OR mugil curema[Tiab] OR atlantic cod[Tiab] OR shark[Tiab] OR sharks[Tiab] OR catshark[Tiab] OR anguilla[Tiab] OR salmonid[Tiab] OR salmonids[Tiab] OR whitefish[Tiab] OR whitefishes[Tiab] OR salmon[Tiab] OR salmons[Tiab] OR sole[Tiab] OR solea[Tiab] OR "sea lamprey"[Tiab] OR lamprey[Tiab] OR lampreys[Tiab] OR pumpkinseed[Tiab] OR sunfish[Tiab] OR sunfishes[Tiab] OR tilapia[Tiab] OR tilapias[Tiab] OR turbot[Tiab] OR turbots[Tiab] OR flatfish[Tiab] OR flatfishes[Tiab] OR sciuridae[Tiab] OR squirrel[Tiab] OR squirrels[Tiab] OR chipmunk[Tiab] OR chipmunks[Tiab] OR suslik[Tiab] OR susliks[Tiab] OR vole[Tiab] OR voles[Tiab] OR lemming[Tiab] OR lemmings[Tiab] OR muskrat[Tiab] OR muskrats[Tiab] OR lemmus[Tiab] OR otter[Tiab] OR otters[Tiab] OR marten[Tiab] OR martens[Tiab] OR martes[Tiab] OR weasel[Tiab] OR badger[Tiab] OR badgers[Tiab] OR ermine[Tiab] OR mink[Tiab] OR minks[Tiab] OR sable[Tiab] OR sables[Tiab] OR gulo[Tiab] OR gulos[Tiab] OR wolverine[Tiab] OR wolverines[Tiab] OR minks[Tiab] OR mustela[Tiab] OR llama[Tiab] OR llamas[Tiab] OR alpaca[Tiab] OR alpacas[Tiab] OR camelid[Tiab] OR camelids[Tiab] OR guanaco[Tiab] OR guanacos[Tiab] OR chiroptera[Tiab] OR chiropteras[Tiab] OR bat[Tiab] OR bats[Tiab] OR fox[Tiab] OR foxes[Tiab] OR iguana[Tiab] OR iguanas[Tiab] OR xenopus laevis[Tiab] OR parakeet[Tiab] OR parakeets[Tiab] OR parrot[Tiab] OR parrots[Tiab] OR donkey[Tiab] OR donkeys[Tiab] OR mule[Tiab] OR mules[Tiab] OR zebra[Tiab] OR zebras[Tiab] OR shrew[Tiab] OR shrews[Tiab] OR bison[Tiab] OR bisons[Tiab] OR buffalo[Tiab] OR buffaloes[Tiab] OR deer[Tiab] OR deers[Tiab] OR bear[Tiab] OR bears[Tiab] OR panda[Tiab] OR pandas[Tiab] OR "wild hog"[Tiab] OR "wild boar"[Tiab] OR fitchew[Tiab] OR fitch[Tiab] OR beaver[Tiab] OR beavers[Tiab] OR jerboa[Tiab] OR jerboas[Tiab] OR capybara[Tiab] OR capybaras[Tiab]) NOT medline[subset])

**Concept 2: Disease Model – Ischemic Stroke Model**

(("brain"[MeSH Terms:noexp] OR "middle cerebral artery"[MeSH Terms:noexp] OR "anterior cerebral artery"[MeSH Terms:noexp] OR "brain"[MeSH Terms] OR "anterior cerebral artery"[MeSH Terms] OR ("brain"[Title/Abstract] OR "cerebrovascular"[Title/Abstract] OR "middle cerebral artery"[Title/Abstract] OR "MCA"[Title/Abstract] OR "ACA"[Title/Abstract] OR "anterior cerebral artery"[Title/Abstract] OR "MCAO"[Title/Abstract])) AND ("stroke"[MeSH Terms:noexp] OR "stroke"[MeSH Terms] OR "ischemia"[MeSH Terms:noexp] OR "ischemia"[MeSH Terms] OR ("stroke"[Title/Abstract] OR "ischemia"[Title/Abstract]))) OR ("infarction, middle cerebral artery"[MeSH Terms] OR "infarction, anterior cerebral artery"[MeSH Terms] OR ("infarction"[Title/Abstract] AND "cerebral"[Title/Abstract] AND "artery"[Title/Abstract]) OR ("cerebral"[Title/Abstract] AND "artery"[Title/Abstract] AND "stroke"[Title/Abstract]) OR (("ischaemic"[Title/Abstract] OR "ischemic"[Title/Abstract]) AND "stroke"[Title/Abstract]) OR ("occlusive"[Title/Abstract] AND "stroke"[Title/Abstract]) OR ("MCAO"[Title/Abstract] OR "ACAO"[Title/Abstract]) OR (("MCA"[Title/Abstract] OR "ACA"[Title/Abstract]) AND "occlusion"[Title/Abstract]) OR (("cerebral"[Title/Abstract] OR "brain"[Title/Abstract]) AND "artery"[Title/Abstract] AND "occlusion"[Title/Abstract]) OR "PMCAO"[Title/Abstract] OR "TMCAO"[Title/Abstract] OR ("focal"[Title/Abstract] AND ("ischemia"[Title/Abstract] OR "ischaemia"[Title/Abstract])) OR ("focal"[Title/Abstract] AND "cerebral"[Title/Abstract] AND ("ischemia"[Title/Abstract] OR "ischaemia"[Title/Abstract])) OR ("focal"[Title/Abstract] AND ("ischaemic"[Title/Abstract] OR "ischemic"[Title/Abstract]) AND "insult"[Title/Abstract]) OR ("experimental"[Title/Abstract] AND "stroke"[Title/Abstract]) OR ("experimental"[Title/Abstract] AND ("brain"[Title/Abstract] OR "cerebral"[Title/Abstract]) AND ("ischemia"[Title/Abstract] OR "ischaemia"[Title/Abstract])) OR ("experimentally"[Title/Abstract] AND "induced"[Title/Abstract] AND ("brain"[Title/Abstract] OR "cerebral"[Title/Abstract]) AND ("ischemia"[Title/Abstract] OR "ischaemia"[Title/Abstract])) OR ("cerebral"[Title/Abstract] AND "stroke"[Title/Abstract]) OR ("encephalic"[Title/Abstract] AND "vascular"[Title/Abstract] AND "accident"[Title/Abstract]) OR (("cerebro-vascular"[Title/Abstract] OR "cerebrovascular"[Title/Abstract]) AND "accident"[Title/Abstract]) OR (("cerebral"[Title/Abstract] OR "brain"[Title/Abstract]) AND "vascular"[Title/Abstract] AND "accident"[Title/Abstract]) OR (("cerebro-vascular"[Title/Abstract] OR "cerebrovascular"[Title/Abstract]) AND "occlusion"[Title/Abstract]) OR ("cerebral"[Title/Abstract] AND "vascular"[Title/Abstract] AND "occlusion"[Title/Abstract]) OR (("brain"[Title/Abstract] OR "cerebral"[Title/Abstract]) AND ("infarct"[Title/Abstract] OR "infarction"[Title/Abstract])) OR (("cerebrovascular"[Title/Abstract] OR "cerebro-vascular"[Title/Abstract]) AND ("infarction"[Title/Abstract] OR "infarct"[Title/Abstract])) OR ("cortical"[Title/Abstract] AND ("infarction"[Title/Abstract] OR "infarct"[Title/Abstract])) OR "stroke model"[Title/Abstract] OR (("embolic"[Title/Abstract] OR "thrombotic"[Title/Abstract]) AND "stroke"[Title/Abstract]) OR (("ischemic"[Title/Abstract] OR "ischaemic"[Title/Abstract]) AND "brain"[Title/Abstract] AND "injury"[Title/Abstract]))

**Concept 3: Intervention – hydrogen sulfide**

("hydrogen sulfide"[Mesh] OR "hydrogen sulfide"[Mesh:noexp] OR "hydrogen sulfide"[tiab] OR "hydrogen sulphide"[tiab] OR H2S[tiab] OR thiosulfates[Mesh] OR thiosulfates[Mesh:noexp] OR thiosulfate[tiab] OR thiosulphate[tiab] OR NaHS[tiab] OR "Sodium hydrosulfide"[tiab] OR Na2S[tiab] OR "Sodium sulfide"[tiab] OR Thioglycine[tiab] OR "H2S donor"[tiab] OR H2S-releasing[tiab] OR H2S-generating[tiab] OR "H2S donating"[tiab] OR "sulfide donor"[tiab])

NOT

(review[Publication Type] OR comment[Publication Type])

**2. EMBASE and Medline Search Criteria**

(paste the complete query into the search box, and deselect “Map Term to Subject Heading”)

**Concept 1: Population – Animal Filter EMBASE (van der Mierden et al. 2022)**

(exp animal experiment/ OR exp animal model/ OR exp experimental animal/ OR exp transgenic animal/ OR exp male animal/ OR exp female animal/ OR exp juvenile animal/ OR animal/ OR chordata/ OR vertebrate/ OR tetrapod/ OR exp fish/ OR amniote/ OR exp amphibia/ OR mammal/ OR exp reptile/ OR exp sauropsid/ OR therian/ OR exp monotreme/ OR placental mammal/ OR exp marsupial/ OR Euarchontoglires/ OR exp Afrotheria/ OR exp Boreoeutheria/ OR exp Laurasiatheria/ OR exp Xenarthra/ OR primate/ OR exp Dermoptera/ OR expGlires/ OR exp Scandentia/ OR Haplorhini/ OR exp prosimian/ OR simian/ OR exp tarsiiform/ OR Catarrhini/ OR exp Platyrrhini/ OR ape/ OR exp Cercopithecidae/ OR hominid/ OR exp hylobatidae/ OR exp chimpanzee/ OR exp gorilla/ OR exp orang utan/ OR exp cephalopod/) OR (rat OR rats OR animal OR animals OR mice OR "in vivo" OR mouse OR rabbit OR rabbits OR murine OR pig OR pigs OR dog OR dogs OR bovine OR fish OR vertebrate OR vertebrates OR cat OR cats OR rodent OR rodents OR mammal OR mammals OR chicken OR chickens OR monkey OR monkeys OR sheep OR canine OR canines OR porcine OR cattle OR bird OR birds OR hamster OR hamsters OR primate OR primates OR cow OR cows OR chick OR horse OR horses OR avian OR avians OR calf OR swine OR swines OR xenopus OR turkeys OR bear OR bears OR frog OR frogs OR zebrafish OR goat OR goats OR equine OR calves OR poultry OR macaque OR macaques OR mole OR moles OR ovine OR lamb OR lambs OR fishes OR diptera OR amphibian OR amphibians OR snake OR snakes OR ruminant OR ruminants OR henOR hens OR piglet OR piglets OR feline OR felines OR simian OR simians OR laevis OR trout OR trouts OR teleost OR teleosts OR salmon OR salmons OR seal OR seals OR bull OR bulls OR ewe OR ewes OR hedgehog OR hedgehogs OR macaca OR macacas OR proteus OR pigeon OR pigeons OR bat OR bats OR duck OR ducks OR chimpanzee OR chimpanzees OR baboon OR baboons OR deer OR deers OR rana OR ranas OR carp OR carps OR heifer OR swallow OR swallows OR lizard OR lizards OR canis OR sow OR sows OR cynomolgus OR quail OR quails OR reptile OR reptiles OR turtle OR turtles OR buffalo OR gerbil OR gerbils OR boar OR boars OR squirrel OR squirrels OR oncorhynchus OR mus OR toad OR toads OR fowl OR fowls OR rerio OR danio OR ara OR aras OR musculus OR tadpole OR tadpoles OR mulatta OR salmo OR ram OR eagle OR eagles OR ferret OR ferrets OR goldfish OR catfish OR whale OR whales OR fox OR foxes OR ape OR apes OR elephant OR elephants OR bos OR marmoset OR marmosets OR cod OR cods OR shark OR sharks OR wolf OR eel OR eels OR auratus OR rattus OR zebra OR zebras OR tilapia OR tilapias OR gilt OR camel OR camels OR squid OR gallus OR marsupial OR marsupials OR vole OR voles OR fascicularis OR ovis OR salmonid OR salmonids OR tiger OR tigers OR dolphin OR dolphins OR robin OR robins OR carpio OR opossum OR opossums OR cyprinus OR salamander OR salamanders OR felis OR mink OR minks OR swan OR swans OR norvegicus OR bufo OR torpedo OR bass OR lamprey OR lampreys OR sus OR python OR pythons OR tetrapod OR tetrapods OR shrew OR shrews OR lionOR lions OR hog OR hogs OR songbird OR songbirds OR oreochromis OR starling OR starlings OR caprine OR carassius OR owl OR owls OR newt OR newts OR papio OR scrofa OR hare OR hares OR gorilla OR gorillas OR flounder OR flounders OR goose OR herring OR herrings OR therian OR buffaloes OR canary OR sparrow OR sparrows OR microtus OR octopus OR troglodytes OR tuna OR amphibia OR chinchilla OR chinchillas OR ide OR oryzias OR cervus OR kangaroo OR kangaroos OR armadillo OR armadillos OR callithrix OR "pan troglodytes" OR saimiri OR cichlid OR cichlids OR donkey OR donkeys OR bream OR char OR chars OR finch OR raccoon OR raccoons OR bothrops OR anguilla OR perch OR cricetus OR seabird OR seabirds OR buck OR bucks OR naja OR coturnix OR salmonids OR geese OR minnow OR minnows OR raptor OR raptors OR merione OR meriones OR rodentia OR elaphus OR amniote OR amniotes OR elasmobranch OR emu OR emus OR peromyscus OR hominid OR hominids OR bubalus OR crotalus OR gull OR gulls OR anas OR anura OR lemur OR lemurs OR crow OR crows OR camelus OR gibbon OR gibbons OR waterfowl OR parrot OR parrots OR eels OR cob OR stickleback OR sticklebacks OR columba OR mesocricetus OR ambystoma OR raven OR ravens OR gadus OR penguin OR penguins OR orangutan OR orangutans OR sturgeon OR sturgeons OR cuniculus OR aves OR virginianus OR cephalopod OR cephalopods OR cebus OR sparus OR tortoise OR tortoises OR guttata OR morhua OR unguiculatus OR dogfish OR vulpes OR mallard OR mallards OR apodemus OR alligator OR alligators OR oryctolagus OR llama OR llamas OR reindeer OR mustela OR duckling OR ducklings OR wolves OR sander OR amazona OR zebu OR badger OR badgers OR dove OR doves OR ictalurus OR capra OR capras OR equus OR camelid OR camelids OR poecilia OR mule OR mules OR perciformes OR salvelinus OR labrax OR cyprinidae OR ariidae OR crocodile OR crocodiles OR fundulus OR dicentrarchus OR clarias OR cercopithecus OR chiroptera OR alpaca OR alpacas OR pike OR pikes OR paralichthys OR puma OR pumas OR didelphis OR pisces OR macropus OR triturusOR bison OR bisons OR epinephelus OR gasterosteus OR panthera OR acipenser OR mackerel OR mackerels OR tamarin OR tamarins OR ostrich OR anolis OR vervet OR vervets OR wallaby OR glareolus OR beaver OR beavers OR dromedary OR catus OR killifish OR pimephales OR promelas OR aotus OR phoca OR panda OR pandas OR porpoise OR porpoises OR myotis OR yak OR yaks OR agkistrodon OR vipera OR otter OR otters OR turbot OR turbots OR squamate OR carnivora OR mullet OR mullets OR hawk OR hawks OR taeniopygia OR seahorse OR seahorses OR "poecilia reticulata" OR falcon OR falcons OR prosimian OR prosimians OR parus OR perca OR fingerling OR fingerlings OR antelope OR antelopes OR tupaia OR passeriformes OR sepia OR saguinus OR coyote OR coyotes OR pongo OR meleagris OR reptilia OR lepus OR psittacine OR hagfish OR warbler OR warblers OR "russell s viper" OR "russell s vipers" OR smolt OR smolts OR budgerigar OR sardine OR sardines OR cavia OR cavias OR hyla OR pleurodeles OR siluriformes OR "great tit" OR "great tits" OR guppy OR bonobo OR bonobos OR rutilus OR trichosurus OR muridae OR phodopus OR channa OR squalus OR lynx OR sturnus OR petromyzon OR vitulina OR monodelphis OR cuttlefish OR adder OR adders OR lepomis OR canaria OR gambusia OR guppies OR xiphophorus OR flatfish OR koala OR koalas OR labeo OR stingray OR stingrays OR chelonia OR lampetra OR spermophilus OR crocodilian OR "passer domesticus" OR sciurus OR artiodactyla OR ranidae OR corvus OR necturus OR platypus OR canaries OR bovid OR lagopus OR trimeresurus OR gariepinus OR marten OR martens OR drosophilidae OR mugil OR sunfish OR porcellus OR cypriniformes OR alouatta OR scophthalmus OR anser OR electrophorus OR putorius OR iguana OR iguanas OR lama OR lamas OR takifugu OR circus OR eptesicus OR flycatcher OR galago OR galagos OR trachemys OR lungfish OR characiformes OR shorebird OR shorebirds OR giraffe OR giraffes OR micropterus OR scyliorhinus OR cichlidae OR loligo OR porcupine OR porcupines OR chub OR chubs OR solea OR pleuronectes OR hylidae OR viperidae OR echis OR sorex OR anchovy OR lagomorph OR ostriches OR vulture OR vultures OR whitefish OR araneus OR jird OR jirds OR tern OR esox OR drake OR drakes OR elapidae OR gallopavo OR chordata OR myodes OR caretta OR serinus OR grouse OR misgurnus OR meles OR blackbird OR blackbirds OR coregonus OR bobwhite OR bobwhites OR heteropneustes OR mammoth OR mammoths OR turdus OR rhinella OR ateles OR characidae OR clupea OR bungarus OR brill OR "struthio camelus" OR sloth OR sloths OR pteropus OR sculpin OR anthropoids OR pollock OR pollocks OR morone OR "pan paniscus" OR litoria OR chipmunk OR chipmunks OR balaenoptera OR marmota OR melopsittacus OR hyrax OR lemming OR lemmings OR halibut OR hylobates OR lates OR caiman OR caimans OR sigmodon OR stenella OR barbel OR barbels OR sterna OR parakeet OR parakeets OR phocoena OR leptodactylus OR canidae OR buteo OR harengus OR gopher OR gophers OR marmot OR marmots OR gosling OR goslings OR platichthys OR gar OR gars OR sebastes OR marsupialia OR notophthalmus OR gazelle OR gazelles OR insectivora OR paridae OR felidae OR russula OR galliformes OR bombina OR colobus OR echidna OR echidnas OR seabass OR syncerus OR plaice OR "blue tit" OR "blue tits" OR pagrus OR catfishes OR cetacea OR barbus OR cygnus OR ficedula OR chamois OR colubridae OR perches OR coelacanth OR fitch OR urodela OR cynops OR martes OR halichoerus OR aix OR salmonidae OR leuciscus OR magpie OR magpies OR silurus OR whiting OR whitings OR anseriformes OR colinus OR rhea OR chlorocebus OR octodon OR acinonyx OR mouflon OR mouflons OR ibex OR tetraodon OR bufonidae OR equidae OR jackal OR cephalopoda OR dendroaspis OR glama OR muskrat OR muskrats OR sable OR sables OR wildebeest OR streptopelia OR albifrons OR vespertilionidae OR woodpecker OR woodpeckers OR muntjac OR muntjacs OR archosaur OR branta OR cricetulus OR megalobrama OR poeciliidae OR desmodus OR snakehead OR snakeheads OR tench OR teal OR teals OR bandicoot OR bandicoots OR apteronotus OR phyllostomidae OR crocidura OR buzzard OR buzzards OR larimichthys OR cercocebus OR pipistrellus OR erithacus OR impala OR impalas OR rousettus OR haddock OR haddocks OR tinca OR ratite OR calidris OR cynoglossus OR hypophthalmichthys OR bullock OR bullocks OR dromedaries OR alectoris OR filly OR salamandra OR cingulata OR bitis OR grus OR ammodytes OR macaw OR macaws OR hypoleuca OR sapajus OR cyprinodontiformes OR hippopotamus OR pelophylax OR capybara OR capybaras OR weasel OR weasels OR cairina OR cynomys OR lutra OR cockatoo OR cockatoos OR lachesis OR lagomorpha OR rupicapra OR daboia OR "orang utan" OR "orang utans" OR platyrrhini OR charadriiformes OR micrurus OR psittaciformes OR spalax OR loris OR mustelidae OR sylvilagus OR vitticeps OR cockatiel OR mustelus OR cottus OR erythrocebus OR dipodomys OR platessa OR callicebus OR loricariidae OR catostomus OR cuneata OR cyanistes OR cyprinodon OR sigmodontinae OR elasmobranchii OR trichechus OR sauropsid OR xenarthra OR dormouse OR perissodactyla OR nautilus OR cirrhinus OR gulo OR gulos OR tragelaphus OR merula OR numida OR sciaenidae OR cerastes OR sciuridae OR gibbosus OR octopuses OR eland OR elands OR phyllomedusa OR pogona OR walrus OR agamidae OR leptodactylidae OR ridibundus OR leontopithecus OR anteater OR anteaters OR pelodiscus OR cebidae OR columbianus OR "pelteobagrus fulvidraco" OR hominoidea OR mandrillus OR "zonotrichia leucophrys" OR agama OR gobiocypris OR "bearded dragon" OR "bearded dragons" OR sarotherodon OR talpa OR discoglossus OR hagfishes OR sphenodon OR gudgeon OR amphiuma OR aythya OR tenrec OR tenrec OR hominidae OR risoria OR salamandridae OR camelidae OR columbiformes OR latimeria OR plover OR plovers OR afrotheria OR "falco sparverius" OR polecat OR polecats OR crotalinae OR salvadora OR tarsier OR lucioperca OR anchovies OR lungfishes OR terrapin OR "dromaius novaehollandiae" OR lateolabrax OR eigenmannia OR pelamis OR theropithecus OR murinae OR gander OR gymnotus OR pseudacris OR gymnophiona OR gymnotiformes OR laticauda OR falconiformes OR dugong OR dugongs OR pintail OR pintails OR rook OR rooks OR lasiurus OR catshark OR catsharks OR micropogonias OR "red junglefowl" OR paddlefish OR ophiophagus OR hollandicus OR nymphicus OR pimelodidae OR aepyceros OR cobitidae OR strigiformes OR cobitis OR dormice OR alytes OR calloselasma OR guanaco OR guanacos OR phasianidae OR "round goby" OR trichogaster OR catarrhini OR eelpout OR eelpouts OR galaxias OR gaur OR pungitius OR suslik OR susliks OR flatfishes OR percidae OR caprinae OR todarodes OR osmerus OR ameiurus OR anthropoidea OR"castor canadensis" OR pouting OR poutings OR tetraodontiformes OR arvicolinae OR siamang OR siamangs OR "castor fiber" OR nomascus OR "red knot" OR "red knots" OR syngnathidae OR iguanidae OR eretmochelys OR ursidae OR callimico OR columbidae OR microhylidae OR anaxyrus OR menidia OR pipistrelle OR greylag OR pipidae OR scandentia OR bowfin OR bowfins OR dendrobatidae OR zenaida OR bushbaby OR harrier OR harriers OR macropodidae OR pygerythrus OR clupeidae OR odorrana OR corvidae OR jerboa OR jerboas OR canutus OR hylobatidae OR clupeiformes OR "great cormorant" OR "great cormorants" OR scorpaeniformes OR chondrostean OR garfish OR proboscidea OR psetta OR diapsid OR serotinus OR tetrao OR walruses OR carcharhiniformes OR leucoraja OR pumpkinseed OR dosidicus OR acipenseriformes OR daubentonii OR emberizidae OR gadiformes OR hyraxes OR stizostedion OR wolverine OR wolverines OR lissotriton OR acanthurus OR centrarchidae OR gloydius OR laurasiatheria OR limosa OR psittacula OR leporidae OR proteidae OR zander OR zanders OR arapaima OR bagridae OR cyprinodontidae OR mithun OR pandion OR jackdaw OR jackdaws OR procyonidae OR carus OR jaculus OR salmoniformes OR "common sole" OR "common soles" OR protobothrops OR calamita OR brachyteles OR trionyx OR turdidae ORboidae OR luscinia OR pugnax OR euarchontoglires OR saithe OR saithes OR symphalangus OR aardvark OR aardvarks OR oystercatcher OR oystercatchers OR arius OR corydoras OR poacher OR poachers OR aurochs OR cebuella OR crecca OR lemuridae OR sirenia OR lemmus OR perdix OR glires OR lepidosaur OR muskox OR deinagkistrodon OR pholidota OR holocephali OR cercopithecinae OR clariidae OR agapornis OR doryteuthis OR tyrannidae OR dicroglossidae OR godwit OR godwits OR monedula OR pongidae OR atheriniformes OR colobinae OR lophocebus OR atelidae OR cottidae OR leucopsis OR acanthuridae OR didelphimorphia OR elver OR elvers OR lapponica OR dermoptera OR "european hake" OR "european hakes" OR gerbillinae OR banteng OR hartebeest OR hartebeests OR hogget OR haematopus OR "anguis fragilis" OR "grey heron" OR "grey herons" OR "blue whiting" OR "blue whitings" OR furnariidae OR macrovipera OR esocidae OR lapwing OR lapwings OR mylopharyngodon OR wallabia OR beloniformes OR potoroo OR potoroos OR "athene noctua" OR pleuronectidae OR bushbabies OR muscicapidae OR alligatoridae OR fuligula OR "bush baby" OR guineafowl OR spoonbill OR spoonbills OR viverridae OR catostomidae OR zebrafishes OR ibexes OR vendace OR estrildidae OR monotremata OR sepiella OR ambystomatidae OR shelduck OR shelducks OR treeshrew OR treeshrews OR hoplobatrachus OR pochard OR hoolock OR hoolocks OR lynxes OR antilope OR antilopes OR blackbuck OR blackbucks OR cricetinae OR paramisgurnus OR skylark OR skylarks OR soleidae OR allobates OR "northern wheatear" OR "northern wheatears" OR pitheciidae OR takin OR theria OR vanellus OR galaxiidae OR lorisidae OR ostralegus OR palaeognathae OR "stone loach" OR alauda OR callitrichinae OR caniformia OR duttaphrynus OR ictaluridae OR osteoglossiformes OR poultries OR curema OR "ruddy turnstone" OR "ruddy turnstones" OR sheatfish OR sunfishes OR centropomidae OR hemachatus OR platalea OR thamnophilidae OR "song thrush" OR atherinopsidae OR siluridae OR tadorna OR chroicocephalus OR ermine OR ermines OR gavialis OR ruff OR tupaiidae OR diprotodontia OR hyaenidae OR antilopinae OR crocodylidae OR herpestidae OR hippopotamidae OR "northern shoveler" OR "round gobies" OR cheirogaleidae OR indriidae OR fundulidae OR pythonidae OR rhynchocephalia OR anodorhynchus OR "red-backed shrike" OR "red-backed shrikes" OR triakidae OR phalangeridae OR aoudad OR boreoeutheria OR "eurasian jay" OR "eurasian jays" OR feliformia OR haplorhini OR osteoglossidae OR paenungulata OR struthioniformes OR ferina OR sanderling OR sanderlings OR spheniscidae OR cuttlefishes OR cygnet OR dasycneme OR gadwall OR gadwalls OR "pelobates fuscus" OR wryneck OR wrynecks OR afrosoricida OR culaea OR "dover sole" OR "dover soles" OR paralichthyidae OR passeridae OR osteolaemus OR "song thrushes" OR bluethroat OR bluethroats OR hydrophiidae OR megrim OR mephitidae OR strepsirhini OR tomistoma OR epidalea OR osmeriformes OR "bush babies" OR tarsiiform OR atelinae OR bufotes OR "eurasian coot" OR "eurasian coots" OR galagidae OR geopelia OR philomachus OR tubulidentata OR bombinatoridae OR pelobatidae OR tachysurus OR ailuridae OR woodlark OR woodlarks OR alcelaphinae OR redshank OR redshanks OR salientia OR "sand smelt" OR "sand smelts" OR woodmice OR woodmouse OR dasyproctidae OR "eurasian wigeon" OR "eurasianwigeons" OR garganey OR garganeys OR "lemon sole" OR "lemon soles" OR "common dab" OR "common dabs" OR graylag OR graylags OR leucorodia OR osphronemidae OR bewickii OR "common moorhen" OR "common moorhens" OR decapodiformes OR gobbler OR gobblers OR odontophoridae OR paddlefishes OR eutheria OR salmonine OR esociformes OR "eurasian woodcock" OR "eurasian woodcocks" OR "european smelt" OR "european smelts" OR goldfishes OR tenches OR tyranni OR "common chaffinch" OR "common chaffinchs" OR "common redstart"OR "common redstarts" OR "common roach" OR "common roachs" OR "great knot" OR "great knots" OR potoroidae OR alytidae OR coregonine OR dipteral OR leveret OR "poeciliopsis gracilis" OR amphiumidae OR batrachoidiformes OR "bighead goby" OR heteropneustidaeOR lullula OR "norway pout" OR "norway pouts" OR sipunculida OR dogfishes OR sebastidae OR tarsiidae OR alethinophidia OR "common nase" OR "common nases" OR "common sandpiper" OR "common sandpipers" OR "eurasian blackcap" OR "eurasian blackcaps" OR pterocnemia OR syngnathiformes OR "common chaffinches" OR eupleridae OR octopodiformes OR phascolarctidae OR scophthalmidae OR "starry smooth-hound" OR "starry smooth-hounds" OR whitefishes OR cuniculidae OR "european sprat" OR "european sprats" OR "rosy bitterling" OR "rosy bitterlings" OR "common dace" OR "common daces" OR "lesser weever" OR "lesser weevers" OR scaldfish OR "water rail" OR "water rails" OR alouattinae OR centrarchiformes OR "common whitethroat" OR "common whitethroats" OR gavialidae OR "grey gurnard" OR "grey gurnards" OR lateolabracidae OR rheiformes OR "tub gurnard" OR "tub gurnards" OR "common chiffchaff" OR "common chiffchaffs" OR garfishes OR "lesser whitethroat" OR "lesser whitethroats" OR myoxidae OR seabasses OR spariformes OR umbridae OR "yellow boxfish" OR anabantiformes OR aotidae OR "common bleak" OR "common bleaks" OR "common rudd" OR "common rudds" OR "greater pipefish" OR hapale OR nandiniidae OR "stone loaches" OR whinchat OR whinchats OR acanthuriformes OR "brotula barbata" OR "common ling" OR "common lings" OR "common roaches" OR cottonrat OR cottonrats OR douroucoulis OR dromaiidae OR fitches OR fitchew OR galaxiiformes OR laprine OR saimiriinae OR solenette OR tarsii OR "tompot blenny" OR "common dragonet" OR "common dragonets"OR "longspined bullhead" OR "longspined bullheads" OR monotremate OR monotremates OR pempheriformes OR perdicinae OR presbytini OR smegmamorpha OR "bighead gobies" OR "carangaria incertae sedis" OR coiidae OR "fivebeard rockling" OR foulmart OR foumart ORgrasskeet OR "greater pipefishes" OR ibices OR millionfish OR muguliformes OR "norwegian topknot" OR peewit OR "red sea sailfin tang" OR rupicapras OR sheatfishes OR "tompot blennies" OR "twait shad" OR "yellow boxfishes").ti,ab,kw.

**Concept 2: Disease Model – Ischemic Stroke Model**

((stroke/ OR exp stroke/ OR ischemia/ OR exp ischemia/) OR (stroke.tw. OR ischemia.tw.)) AND (brain/ OR "middle cerebral artery"/ OR "anterior cerebral artery"/ OR brain.tw. OR cerebrovascular.tw. OR "middle cerebral artery".tw. OR MCA.tw. OR ACA.tw. OR "anterior cerebral artery".tw. OR MCAO.tw.) OR ((Infarction, middle cerebral artery/) or (Infarction, anterior cerebral artery/)) or ((infarct* or stroke) and cerebral adj3 arter*).ti,ab,kw. or ((isch?emic or cerebral) and (stroke)).ti,ab,kw. or (occlusive and stroke).ti,ab,kw. or (MCAO or (MCA adj5 occlusion)).ti,ab,kw. or (ACAO or (ACA adj5 occlusion)).ti,ab,kw. or (PMCAO or TMCAO).ti,ab,kw. or ((cerebral or brain) and artery and occlusion).ti,ab,kw. or ((focal or cerebral) and isch?emia and insult).ti,ab,kw. or ((experiment* or embolic or thrombotic) and (brain adj5 stroke)).ti,ab,kw. or (experimental and (brain or cerebral) and isch?mia).ti,ab,kw. or (encephalic and vascular and accident).ti,ab,kw. or (cerebro?vascular and (accident or occlusion or infarct*)).ti,ab,kw. or (cerebral and (accident or occlusion)).ti,ab,kw. or (vascular occlusion and (brain or cerebral or cortical)).ti,ab,kw. or ((brain or cerebral or cortical) and infarct*).ti,ab,kw. or (stroke model?).ti,ab,kw. or (isch?emic and brain and injury).ti,ab,kw.

**Concept 3: Intervention – hydrogen sulfide**

exp Hydrogen Sulfide/ OR "hydrogen sulfide".ti,ab,kw. OR "hydrogen sulfide".tw. OR

exp hydrogen sulphide/ OR "hydrogen sulphide".ti,ab,kw. OR "hydrogen sulphide".tw. OR

exp H2S/ OR H2S.ti,ab,kw. OR H2S.tw. OR

exp thiosulfate/ OR thiosulfate.ti,ab,kw. OR thiosulfate.tw. OR

exp thiosulphate/ OR thiosulphate.ti,ab,kw. OR thiosulphate.tw. OR

exp NaHS/ OR NaHS.ti,ab,kw. OR NaHS.tw. OR

exp Sodium hydrosulfide/ OR "Sodium hydrosulfide".ti,ab,kw. OR "Sodium hydrosulfide".tw. OR

exp Sodium sulfide/ OR "Sodium sulfide".ti,ab,kw. OR "Sodium sulfide".tw. OR

exp Na2S/ OR Na2S.ti,ab,kw. OR Na2S.tw. OR

exp Thioglycine/ OR Thioglycine.ti,ab,kw. OR Thioglycine.tw. OR

exp H2S donor/ OR "H2S donor".ti,ab,kw. OR "H2S donor".tw. OR

exp sulfide donor/ OR "sulfide donor".ti,ab,kw. OR "sulfide donor".tw. OR

exp H2S-releasing/ OR "H2S-releasing ".ti,ab,kw. OR "H2S-releasing".tw. OR

exp H2S-generating/ OR "H2S-generating".ti,ab,kw. OR "H2S-generating".tw. OR

exp H2S-donating/ OR "H2S-donating".ti,ab,kw. OR "H2S-donating".tw. OR

NOT

(review.pt. OR comment.pt.)

**3. Medline Search Criteria (EBSCO)**

((MH "animal experimentation+") OR (MH "models, animal+") OR (MH invertebrates+) OR (MH Animals) OR (MH "animal population groups+") OR (MH chordata) OR (MH "chordata, nonvertebrate+") OR (MH vertebrates) OR (MH amphibians+) OR (MH birds+) OR (MH fishes+) OR (MH reptiles+) OR (MH mammals) OR (MH primates) OR (MH artiodactyla+) OR (MH carnivora+) OR (MH cetacea+) OR (MH chiroptera+) OR (MH elephants+) OR (MH hyraxes+) OR (MH insectivora+) OR (MH lagomorpha+) OR (MH marsupialia+) OR (MH monotremata+) OR (MH perissodactyla+) OR (MH rodentia+) OR (MH scandentia+) OR (MH sirenia+) OR (MH haplorhini) OR (MH strepsirhini+) OR (MH platyrrhini+) OR (MH tarsii+) OR (MH catarrhini) OR (MH cercopithecidae+) OR (MH hylobatidae+) OR (MH hominidae) OR (MH "gorilla gorilla+") OR (MH "pan paniscus+") OR (MH "pan troglodytes+") OR (MH "pongo pygmaeus+")) OR (((TI animals OR AB animals) OR (TI animal OR AB animal) OR (TI mice OR AB mice) OR (TI mus OR AB mus) OR (TI mouse OR AB mouse) OR (TI murine OR AB murine) OR (TI woodmouse OR AB woodmouse) OR (TI rats OR AB rats) OR (TI rat OR AB rat) OR (TI murinae OR AB murinae) OR (TI muridae OR AB muridae) OR (TI cottonrat OR AB cottonrat) OR (TI cottonrats OR AB cottonrats) OR (TI hamster OR AB hamster) OR (TI hamsters OR AB hamsters) OR (TI cricetinae OR AB cricetinae) OR (TI rodentia OR AB rodentia) OR (TI rodent OR AB rodent) OR (TI rodents OR AB rodents) OR (TI pigs OR AB pigs) OR (TI pig OR AB pig) OR (TI swine OR AB swine) OR (TI swines OR AB swines) OR (TI piglets OR AB piglets) OR (TI piglet OR AB piglet) OR (TI boar OR AB boar) OR (TI boars OR AB boars) OR (TI "sus scrofa" OR AB "sus scrofa") OR (TI ferrets OR AB ferrets) OR (TI ferret OR AB ferret) OR (TI polecat OR AB polecat) OR (TI polecats OR AB polecats) OR (TI "mustela putorius" OR AB "mustela putorius") OR (TI "guinea pigs" OR AB "guinea pigs") OR (TI "guinea pig" OR AB "guinea pig") OR (TI cavia OR AB cavia) OR (TI callithrix OR AB callithrix) OR (TI marmoset OR AB marmoset) OR (TI marmosets OR AB marmosets) OR (TI cebuella OR AB cebuella) OR (TI hapale OR AB hapale) OR (TI octodon OR AB octodon) OR (TI chinchilla OR AB chinchilla) OR (TI chinchillas OR AB chinchillas) OR (TI gerbillinae OR AB gerbillinae) OR (TI gerbil OR AB gerbil) OR (TI gerbils OR AB gerbils) OR (TI jird OR AB jird) OR (TI jirds OR AB jirds) OR (TI merione OR AB merione) OR (TI meriones OR AB meriones) OR (TI rabbits OR AB rabbits) OR (TI rabbit OR AB rabbit) OR (TI hares OR AB hares) OR (TI hare OR AB hare) OR (TI diptera OR AB diptera) OR (TI flies OR AB flies) OR (TI fly OR AB fly) OR (TI dipteral OR AB dipteral) OR (TI drosphila OR AB drosphila) OR (TI drosophilidae OR AB drosophilidae) OR (TI cats OR AB cats) OR (TI cat OR AB cat) OR (TI carus OR AB carus) OR (TI felis OR AB felis) OR (TI nematoda OR AB nematoda) OR (TI nematode OR AB nematode) OR (TI nematoda OR AB nematoda) OR (TI nematode OR AB nematode) OR (TI nematodes OR AB nematodes) OR (TI sipunculida OR AB sipunculida) OR (TI dogs OR AB dogs) OR (TI dog OR AB dog) OR (TI canine OR AB canine) OR (TI canines OR AB canines) OR (TI canis OR AB canis) OR (TI sheep OR AB sheep) OR (TI sheeps OR AB sheeps) OR (TI mouflon OR AB mouflon) OR (TI mouflons OR AB mouflons) OR (TI ovis OR AB ovis) OR (TI goats OR AB goats) OR (TI goat OR AB goat) OR (TI capra OR AB capra) OR (TI capras OR AB capras) OR (TI rupicapra OR AB rupicapra) OR (TI chamois OR AB chamois) OR (TI haplorhini OR AB haplorhini) OR (TI monkey OR AB monkey) OR (TI monkeys OR AB monkeys) OR (TI anthropoidea OR AB anthropoidea) OR (TI anthropoids OR AB anthropoids) OR (TI saguinus OR AB saguinus) OR (TI tamarin OR AB tamarin) OR (TI tamarins OR AB tamarins) OR (TI leontopithecus OR AB leontopithecus) OR (TI hominidae OR AB hominidae) OR (TI ape OR AB ape) OR (TI apes OR AB apes) OR (TI pan OR AB pan) OR (TI paniscus OR AB paniscus) OR (TI "pan paniscus" OR AB "pan paniscus") OR (TI bonobo OR AB bonobo) OR (TI bonobos OR AB bonobos) OR (TI troglodytes OR AB troglodytes) OR (TI "pan troglodytes" OR AB "pan troglodytes") OR (TI gibbon OR AB gibbon) OR (TI gibbons OR AB gibbons) OR (TI siamang OR AB siamang) OR (TI siamangs OR AB siamangs) OR (TI nomascus OR AB nomascus) OR (TI symphalangus OR AB symphalangus) OR (TI chimpanzee OR AB chimpanzee) OR (TI chimpanzees OR AB chimpanzees) OR (TI prosimians OR AB prosimians) OR (TI "bush baby" OR AB "bush baby") OR (TI prosimian OR AB prosimian) OR (TI "bush babies" OR AB "bush babies") OR (TI galagos OR AB galagos) OR (TI galago OR AB galago) OR (TI pongidae OR AB pongidae) OR (TI gorilla OR AB gorilla) OR (TI gorillas OR AB gorillas) OR (TI pongo OR AB pongo) OR (TI pygmaeus OR AB pygmaeus) OR (TI "pongo pygmaeus" OR AB "pongo pygmaeus") OR (TI orangutans OR AB orangutans) OR (TI pygmaeus OR AB pygmaeus) OR (TI lemur OR AB lemur) OR (TI lemurs OR AB lemurs) OR (TI lemuridae OR AB lemuridae) OR (TI horse OR AB horse) OR (TI horses OR AB horses) OR (TI pongo OR AB pongo) OR (TI equus OR AB equus) OR (TI cow OR AB cow) OR (TI calf OR AB calf) OR (TI bull OR AB bull) OR (TI chicken OR AB chicken) OR (TI chickens OR AB chickens) OR (TI gallus OR AB gallus) OR (TI quail OR AB quail) OR (TI bird OR AB bird) OR (TI birds OR AB birds) OR (TI quails OR AB quails) OR (TI poultry OR AB poultry) OR (TI poultries OR AB poultries) OR (TI fowl OR AB fowl) OR (TI fowls OR AB fowls) OR (TI reptile OR AB reptile) OR (TI reptilia OR AB reptilia) OR (TI reptiles OR AB reptiles) OR (TI snakes OR AB snakes) OR (TI snake OR AB snake) OR (TI lizard OR AB lizard) OR (TI lizards OR AB lizards) OR (TI alligator OR AB alligator) OR (TI alligators OR AB alligators) OR (TI crocodile OR AB crocodile) OR (TI crocodiles OR AB crocodiles) OR (TI turtle OR AB turtle) OR (TI turtles OR AB turtles) OR (TI amphibian OR AB amphibian) OR (TI amphibians OR AB amphibians) OR (TI amphibia OR AB amphibia) OR (TI frog OR AB frog) OR (TI frogs OR AB frogs) OR (TI bombina OR AB bombina) OR (TI salientia OR AB salientia) OR (TI toad OR AB toad) OR (TI toads OR AB toads) OR (TI "epidalea calamita" OR AB "epidalea calamita") OR (TI salamander OR AB salamander) OR (TI salamanders OR AB salamanders) OR (TI eel OR AB eel) OR (TI eels OR AB eels) OR (TI fish OR AB fish) OR (TI fishes OR AB fishes) OR (TI pisces OR AB pisces) OR (TI catfish OR AB catfish) OR (TI catfishes OR AB catfishes) OR (TI siluriformes OR AB siluriformes) OR (TI arius OR AB arius) OR (TI heteropneustes OR AB heteropneustes) OR (TI sheatfish OR AB sheatfish) OR (TI perch OR AB perch) OR (TI perches OR AB perches) OR (TI percidae OR AB percidae) OR (TI perca OR AB perca) OR (TI trout OR AB trout) OR (TI trouts OR AB trouts) OR (TI char OR AB char) OR (TI chars OR AB chars) OR (TI salvelinus OR AB salvelinus) OR (TI "fathead minnow" OR AB "fathead minnow") OR (TI minnow OR AB minnow) OR (TI cyprinidae OR AB cyprinidae) OR (TI carps OR AB carps) OR (TI carp OR AB carp) OR (TI zebrafish OR AB zebrafish) OR (TI zebrafishes OR AB zebrafishes) OR (TI goldfish OR AB goldfish) OR (TI goldfishes OR AB goldfishes) OR (TI guppy OR AB guppy) OR (TI guppies OR AB guppies) OR (TI chub OR AB chub) OR (TI chubs OR AB chubs) OR (TI tinca OR AB tinca) OR (TI barbels OR AB barbels) OR (TI barbus OR AB barbus) OR (TI pimephales OR AB pimephales) OR (TI promelas OR AB promelas) OR (TI "poecilia reticulata" OR AB "poecilia reticulata") OR (TI mullet OR AB mullet) OR (TI mullets OR AB mullets) OR (TI seahorse OR AB seahorse) OR (TI seahorses OR AB seahorses) OR (TI "mugil curema" OR AB "mugil curema") OR (TI "atlantic cod" OR AB "atlantic cod") OR (TI shark OR AB shark) OR (TI sharks OR AB sharks) OR (TI catshark OR AB catshark) OR (TI anguilla OR AB anguilla) OR (TI salmonid OR AB salmonid) OR (TI salmonids OR AB salmonids) OR (TI whitefish OR AB whitefish) OR (TI whitefishes OR AB whitefishes) OR (TI salmon OR AB salmon) OR (TI salmons OR AB salmons) OR (TI sole OR AB sole) OR (TI solea OR AB solea) OR (TI "sea lamprey" OR AB "sea lamprey") OR (TI lamprey OR AB lamprey) OR (TI lampreys OR AB lampreys) OR (TI pumpkinseed OR AB pumpkinseed) OR (TI sunfish OR AB sunfish) OR (TI sunfishes OR AB sunfishes) OR (TI tilapia OR AB tilapia) OR (TI tilapias OR AB tilapias) OR (TI turbot OR AB turbot) OR (TI turbots OR AB turbots) OR (TI flatfish OR AB flatfish) OR (TI flatfishes OR AB flatfishes) OR (TI sciuridae OR AB sciuridae) OR (TI squirrel OR AB squirrel) OR (TI squirrels OR AB squirrels) OR (TI chipmunk OR AB chipmunk) OR (TI chipmunks OR AB chipmunks) OR (TI suslik OR AB suslik) OR (TI susliks OR AB susliks) OR (TI vole OR AB vole) OR (TI voles OR AB voles) OR (TI lemming OR AB lemming) OR (TI lemmings OR AB lemmings) OR (TI muskrat OR AB muskrat) OR (TI muskrats OR AB muskrats) OR (TI lemmus OR AB lemmus) OR (TI otter OR AB otter) OR (TI otters OR AB otters) OR (TI marten OR AB marten) OR (TI martens OR AB martens) OR (TI martes OR AB martes) OR (TI weasel OR AB weasel) OR (TI badger OR AB badger) OR (TI badgers OR AB badgers) OR (TI ermine OR AB ermine) OR (TI mink OR AB mink) OR (TI minks OR AB minks) OR (TI sable OR AB sable) OR (TI sables OR AB sables) OR (TI gulo OR AB gulo) OR (TI gulos OR AB gulos) OR (TI wolverine OR AB wolverine) OR (TI wolverines OR AB wolverines) OR (TI minks OR AB minks) OR (TI mustela OR AB mustela) OR (TI llama OR AB llama) OR (TI llamas OR AB llamas) OR (TI alpaca OR AB alpaca) OR (TI alpacas OR AB alpacas) OR (TI camelid OR AB camelid) OR (TI camelids OR AB camelids) OR (TI guanaco OR AB guanaco) OR (TI guanacos OR AB guanacos) OR (TI chiroptera OR AB chiroptera) OR (TI chiropteras OR AB chiropteras) OR (TI bat OR AB bat) OR (TI bats OR AB bats) OR (TI fox OR AB fox) OR (TI foxes OR AB foxes) OR (TI iguana OR AB iguana) OR (TI iguanas OR AB iguanas) OR (TI "xenopus laevis" OR AB "xenopus laevis") OR (TI parakeet OR AB parakeet) OR (TI parakeets OR AB parakeets) OR (TI parrot OR AB parrot) OR (TI parrots OR AB parrots) OR (TI donkey OR AB donkey) OR (TI donkeys OR AB donkeys) OR (TI mule OR AB mule) OR (TI mules OR AB mules) OR (TI zebra OR AB zebra) OR (TI zebras OR AB zebras) OR (TI shrew OR AB shrew) OR (TI shrews OR AB shrews) OR (TI bison OR AB bison) OR (TI bisons OR AB bisons) OR (TI buffalo OR AB buffalo) OR (TI buffaloes OR AB buffaloes) OR (TI deer OR AB deer) OR (TI deers OR AB deers) OR (TI bear OR AB bear) OR (TI bears OR AB bears) OR (TI panda OR AB panda) OR (TI pandas OR AB pandas) OR (TI "wild hog" OR AB "wild hog") OR (TI "wild boar" OR AB "wild boar") OR (TI fitchew OR AB fitchew) OR (TI fitch OR AB fitch) OR (TI beaver OR AB beaver) OR (TI beavers OR AB beavers) OR (TI jerboa OR AB jerboa) OR (TI jerboas OR AB jerboas) OR (TI capybara OR AB capybara) OR (TI capybaras OR AB capybaras))

AND

((MH stroke) OR (MH stroke+) OR (MH ischemia) OR (MH ischemia+)) OR ((TI stroke OR AB stroke) OR (TI ischemia OR AB ischemia))

AND

(MH brain) OR (MH "middle cerebral artery") OR (MH "anterior cerebral artery") OR (MH brain+) OR (MH "anterior cerebral artery+") OR ((TI brain OR AB brain) OR (TI cerebrovascular OR AB cerebrovascular) OR (TI "middle cerebral artery" OR AB "middle cerebral artery") OR (TI MCA OR AB MCA) OR (TI ACA OR AB ACA) OR (TI "anterior cerebral artery" OR AB "anterior cerebral artery") OR (TI MCAO OR AB MCAO))

AND

((MH "hydrogen sulfide+") OR (MH "hydrogen sulfide") OR (TI "hydrogen sulfide" OR AB "hydrogen sulfide") OR (TI "hydrogen sulphide" OR AB "hydrogen sulphide") OR (TI H2S OR AB H2S) OR (MH thiosulfates+) OR (MH thiosulfates) OR (TI thiosulfate OR AB thiosulfate) OR (TI thiosulphate OR AB thiosulphate) OR (TI NaHS OR AB NaHS) OR (TI "Sodium hydrosulfide" OR AB "Sodium hydrosulfide") OR (TI Na2S OR AB Na2S) OR (TI "Sodium sulfide" OR AB "Sodium sulfide") OR (TI Thioglycine OR AB Thioglycine) OR (TI "H2S donor" OR AB "H2S donor") OR (TI H2S-releasing OR AB H2S-releasing) OR (TI H2S-generating OR AB H2S-generating) OR (TI "H2S donating" OR AB "H2S donating") OR (TI "sulfide donor" OR AB "sulfide donor"))

**4. WoS Search Criteria**

TS=(rat OR rats OR animal OR animals OR mice OR "in vivo" OR mouse OR rabbit OR rabbits OR murine OR pig OR pigs OR dog OR dogs OR bovine OR fish OR vertebrate OR vertebrates OR cat OR cats OR rodent OR rodents OR mammal OR mammals OR chicken OR chickens OR monkey OR monkeys OR sheep OR canine OR canines OR porcine OR cattle OR bird OR birds OR hamster OR hamsters OR primate OR primates OR cow OR cows OR chick OR horse OR horses OR avian OR avians OR calf OR swine OR swines OR xenopus OR turkeys OR bear OR bears OR frog OR frogs OR zebrafish OR goat OR goats OR equine OR calves OR poultry OR macaque OR macaques OR mole OR moles OR ovine OR lamb OR lambs OR fishes OR diptera OR amphibian OR amphibians OR snake OR snakes OR ruminant OR ruminants OR hen OR hens OR piglet OR piglets OR feline OR felines OR simian OR simians OR laevis OR trout OR trouts OR teleost OR teleosts OR salmon OR salmons OR seal OR seals OR bull OR bulls OR ewe OR ewes OR hedgehog OR hedgehogs OR macaca OR macacus OR proteus OR pigeon orpingtons OR bat OR bats OR duck OR ducks OR chimpanzee OR chimpanzees OR baboon OR baboons OR deer OR rana OR ranks OR carp OR carps OR heifer OR swallow OR swallows OR lizard OR lizards OR canis OR sow OR sows OR cynomolgus OR quail OR quails OR reptile OR reptiles OR turtle OR turtles OR buffalo OR gerbil OR gerbils OR boar OR boars OR squirrel OR squirrels OR oncorhynchus OR mus OR toad OR toads OR fowl OR fowls OR rerio OR danio OR ara OR aras OR musculus OR tadpole OR tadpoles OR mulatta OR salmo OR ram OR eagle OR eagles OR ferret OR ferrets OR goldfish OR catfish OR whale OR whales OR fox OR foxes OR ape OR apes OR elephant OR elephants OR bos OR marmoset OR marmosets OR cod OR cods OR shark OR sharks OR wolf OR eel OR eels OR auratus OR rattus OR zebra OR zebras OR tilapia OR tilapias OR gilt OR camel OR camels OR squid OR gallus OR marsupial OR marsupials OR vole OR voles OR fascicularis OR ovis OR salmonid OR salmonids OR tiger OR tigers OR dolphin OR dolphins OR robin OR robins OR carpio OR opossums opossums OR cyprinus OR salamander OR salamanders OR felis OR mink OR minks OR swan OR swans OR norvegicus OR bufo OR torpedo OR bass OR lamprey OR lampreys OR sus OR python OR pythons OR tetrapod OR tetrapods OR shrew OR shrews OR lion OR lions OR honor hogs OR songbird OR songbirds OR oreochromis OR starling OR starlings OR caprine OR carassius OR owl OR owls OR newt OR newts OR papio OR scrofa OR hare OR hares OR gorilla OR gorillas OR flounder OR flounders OR goose OR herring OR herrings OR therianos buffaloes OR canary OR sparrow OR sparrows OR microtus OR octopus OR troglodytes OR tuna OR amphibia OR chinchilla OR chinchillas OR ide OR oryzias OR cervus OR kangaroo OR kangaroos OR armadillo OR armadillos OR callithrix OR "pan troglodytes" OR saimiri OR cichlid OR cichlids OR donkey OR donkeys OR bream OR char OR chars OR finch OR raccoon OR raccoons OR bothrops OR anguilla OR perch OR cricetus OR seabird OR seabirds OR buck OR bucks OR naja OR coturnix OR salmonids OR geese OR minnow OR minnows ozraptor OR raptors OR meriones OR meriones OR rodentia OR elaphus OR amniote OR amniotes OR elasmobranch OR emu OR emus OR peromyscus OR hominid OR hominids OR bubalus OR crotalus OR gull OR gulls OR anas OR anura OR lemur OR lemurs OR crow OR crows OR camelus OR gibbon OR gibbons OR waterfowl OR parrot OR parrots OR eels OR cob OR stickleback OR sticklebacks OR columba OR mesocricetus OR ambystoma OR raven OR ravens OR gadus OR penguin OR penguins OR orangutan OR orangutans OR sturgeon OR sturgeons OR cuniculus OR aves OR virginianus OR cephalopod OR cephalopods OR cebus OR sparus OR tortoise OR tortoises OR guttata OR morhua OR unguiculatus OR dogfish OR vulpes OR mallard OR mallards OR apodemus OR alligator OR alligators OR oryctolagus OR llama OR llamas OR reindeer OR mustela OR duckling OR ducklings OR wolves OR sander OR amazona OR zebu OR badger OR badgers OR dove OR doves OR ictalurus OR capra OR capgras OR equus OR camelid OR camelids OR poecilia OR mule OR mules OR perciformes OR salvelinus OR labrax OR cyprinidae OR ariidae OR crocodile OR crocodiles OR fundulus OR dicentrarchus OR clarias OR cercopithecus OR chiroptera OR alpaca OR alpacas OR pike OR pikes OR paralichthys OR puma OR pumas OR didelphis OR pisces OR macropus OR triturus OR bison OR bosons OR epinephelus OR gasterosteus OR panthera OR acipenser OR mackerel OR mackerels OR tamarin OR tamarins OR ostrich OR anolis OR vervet OR vervets OR wallaby OR glareolus OR beaver OR beavers OR dromedary OR catus OR killifish OR pimephales OR promelas OR aotus OR phoca OR panda OR pandas OR porpoise OR porpoises OR myotis OR yak OR yaks OR agkistrodon OR vipera OR otter OR otters OR turbot OR turbots OR squamate OR carnivora OR mullet OR mullets OR hawk OR hawks OR taeniopygia OR seahorse OR seahorses OR "poecilia reticulata" OR falcon OR falcons OR prosimian OR prosimians OR parus OR perca OR fingerling OR fingerlings OR antelope OR antelopes OR tupaia OR passeriformes OR sepia OR saguinus OR coyote OR coyotes OR pongo OR meleagris OR reptilia OR lepus OR psittacine OR hagfish OR warbler OR warblers OR "russell s viper" OR "russell s vipers" OR smolt OR smolts OR budgerigar OR sardine OR sardines OR cavia OR caviae OR hyla OR pleurodeles OR siluriformes OR "great tit" OR "great tits" OR guppy OR bonobo OR bonobos OR rutilus OR trichosurus OR muridae OR phodopus OR channa OR squalus OR lynx OR sturnus OR petromyzon OR vitulina OR monodelphis OR cuttlefish OR adder OR adders OR lepomis OR canaria OR gambusia OR guppies OR xiphophorus OR flatfish OR koala orkoulas OR labeo OR stingray OR stingrays OR chelonia OR lampetra OR spermophilus OR crocodilian OR "passer domesticus" OR sciurus OR artiodactyla OR ranidae OR corvus OR necturus OR platypus OR canaries OR bovid OR lagopus OR trimeresurus OR gariepinus ormarked OR martens OR drosophilidae OR mugil OR sunfish OR porcellus OR cypriniformes OR alouatta OR scophthalmus OR anser OR electrophorus OR putorius OR iguana OR iguanas OR lama OR lamas OR takifugu OR circus OR eptesicus OR flycatcher OR galago OR galagos OR trachemys OR lungfish OR characiformes OR shorebird OR shorebirds OR giraffe OR giraffes OR micropterus OR scyliorhinus OR cichlidae OR loligo OR porcupine OR porcupines OR chub OR chubs OR solea OR pleuronectes OR hylidae OR viperidae OR echis OR sorex OR anchovy OR lagomorph OR ostriches OR vulture OR vultures OR whitefish OR araneus OR jird OR jirds OR tern OR esox OR drake OR drakes OR elapidae OR gallopavo OR chordata OR myodes OR caretta OR serinus OR grouse OR misgurnus OR meles OR blackbird OR blackbirds OR coregonus OR bobwhite OR bobwhites OR heteropneustes OR mammoth OR mammoths OR turdus OR rhinella OR ateles OR characidae OR clupea OR bungarus OR brill OR "struthio camelus" OR sloth OR sloths OR pteropus OR sculpin OR anthropoids OR pollock OR pollock OR morone OR "pan paniscus" OR litoria OR chipmunk OR chipmunks OR balaenoptera OR marmota OR melopsittacus OR hyrax OR lemming OR lemmings OR halibut OR hylobates OR lates OR caiman OR caimans OR sigmodon OR stenella OR barbel OR barbels orestera OR parakeet OR parakeets OR phocoena OR leptodactylus OR canidae OR buteo OR harengus OR gopher OR gophers OR marmot OR marmots OR gosling OR goslings OR platichthys OR gar OR gars OR sebastes OR marsupialia OR notophthalmus OR gazelle OR gazelles OR insectivora OR paridae OR felidae OR russula OR galliformes OR bombina OR colobus OR echidna OR echidnas OR seabass OR syncerus OR plaice OR "blue tit" OR "blue tits" OR pagrus OR catfishes OR cetacea OR barbus OR cygnus OR ficedula OR chamois OR colubridae OR perches OR coelacanth OR fitch OR urodela OR cynops OR martes OR halichoerus OR aix OR salmonidae OR leuciscus OR magpie OR magpies OR silurus OR whiting OR writings OR anseriformes OR colinus OR rhea OR chlorocebus OR octodon OR acinonyx OR mouflon OR mouflons OR ibex OR tetraodon OR bufonidae OR equidae OR jackal OR cephalopoda OR dendroaspis OR glama OR muskrat OR muskrats OR sable OR sables OR wildebeest OR streptopelia OR albifrons OR vespertilionidae OR woodpecker OR woodpeckers OR muntjac OR muntjac OR archosaur OR branta OR cricetulus OR megalobrama OR poeciliidae OR desmodus OR snakehead OR snakehead OR tench OR teal OR teals OR bandicoot OR bandicoots OR apteronotus OR phyllostomidae OR crocidura OR buzzard OR buzzards OR larimichthys OR cercocebus OR pipistrellus OR erithacus OR impala OR impala OR rousettus OR haddock OR haddock2 OR tinca OR ratite OR calidris OR cynoglossus OR hypophthalmichthys OR bullock OR bullocks OR dromedaries OR alectoris OR filly OR salamandra OR cingulata OR bitis OR grus OR ammodytes OR macaw OR macaws OR hypoleuca OR sapajus OR cyprinodontiformes OR hippopotamus OR pelophylax OR capybara OR capybaras OR weasel OR weasels OR cairina OR cynomys OR lutra OR cockatoo OR cockatoos OR lachesis OR lagomorpha OR rupicapra OR daboia OR "orang utan" OR "orang utans" OR platyrrhini OR charadriiformes OR micrurus OR psittaciformes OR spalax OR loris OR mustelidae OR sylvilagus OR vitticeps OR cockatiel OR mustelus OR cottus OR erythrocebus OR dipodomys OR platessa OR callicebus OR loricariidae OR catostomus OR cuneata OR cyanistes OR cyprinodon OR sigmodontinae OR elasmobranchii OR trichechus OR sauropsid OR xenarthra OR dormouse OR perissodactyla OR nautilus OR cirrhinus OR gulo OR gulls OR tragelaphus OR merula OR numidier sciaenidae OR cerastes OR sciuridae OR gibbosus OR octopuses OR eland OR elanus OR phyllomedusa OR pogona OR walrus OR agamidae OR leptodactylidae OR ridibundus OR leontopithecus OR anteater OR anteaters OR pelodiscus OR cebidae OR columbianus OR "pelteobagrus fulvidraco" OR hominoidea OR mandrillus OR "zonotrichia leucophrys" OR agama OR gobiocypris OR "bearded dragon" OR "bearded dragons" OR sarotherodon OR talpa OR discoglossus OR hagfishes OR sphenodon OR gudgeon OR amphiuma OR aythya OR tenrec OR tenrec OR hominidae OR risoria OR salamandridae OR camelidae OR columbiformes OR latimeria OR plover OR plovers OR afrotheria OR "falco sparverius" OR polecat OR polecats OR crotalinae OR salvadora OR tarsier OR lucioperca OR anchovies OR lungfishes OR terrapin OR "dromaius novaehollandiae" OR lateolabrax OR eigenmannia OR pelamis OR theropithecus OR murinae OR gander OR gymnotus OR pseudacris OR gymnophiona OR gymnotiformes OR laticauda OR falconiformes OR dugong OR dugongs OR pintail OR pintails OR rook orriols OR lasiurus OR catshark OR catsharks OR micropogonias OR "red junglefowl" OR paddlefish OR eutheria OR ophiophagus OR hollandicus OR nymphicus OR pimelodidae OR aepyceros OR cobitidae OR strigiformes OR cobitis OR dormice OR alytes OR calloselasma OR guanaco OR guanacos OR phasianidae OR "round goby" OR trichogaster OR catarrhini OR eelpout OR eelpouts OR galaxias OR gaur OR pungitius OR suslin OR sousliks OR flatfishes OR percidae OR caprinae OR todarodes OR osmerus OR ameiurus OR anthropoides OR "castor canadensis" OR pouting OR postings OR tetraodontiformes OR arvicolinae OR siamang OR siamangs OR "castor fiber" OR nomascus OR "red knot" OR "red knots" OR syngnathidae OR iguanidae OR eretmochelys OR ursidae OR callimico OR columbidae OR microhylidae anaxyrus OR menidia OR pipistrelle OR greylag OR pipidae OR scandentia OR bowfin OR boffins OR dendrobatidae OR zenaida OR bushbaby OR harrier OR harriers OR macropodidae OR pygerythrus OR clupeidae OR odorrana OR corvidae OR jerboa OR jerboas OR canutus OR hylobatidae OR clupeiformes OR "great cormorant" OR "great cormorants" OR scorpaeniformes OR chondrostean OR garfish OR proboscidea OR psetta OR diapsid OR serotinus OR tetrao OR walruses OR carcharhiniformes OR leucoraja OR pumpkinseed OR dosidicus OR acipenseriformes OR daubentonii OR emberizidae OR gadiformes OR hyraxes OR stizostedion OR wolverine OR wolverines OR lissotriton OR acanthurus OR centrarchidae OR gloydius OR laurasiatherian OR limosa OR psittacula OR leporidae OR proetidae OR zander oleanders OR arapaima OR bagridae OR cyprinodontidae OR mithun OR pandion OR jackdaw OR jackdaws OR procyonidae OR carus OR jaculus OR salmoniformes OR "common sole" OR "common soles" OR protobothrops OR calamita OR brachyteles OR trionyx OR turdidae OR boidae OR luscinia OR pugnax OR euarchontoglires OR saithe OR staithes OR symphalangism OR aardvark OR aardvark OR oystercatcher OR oystercatchers OR arius OR corydoras OR poacher OR poachers OR aurochs OR cebuella OR crecca OR leporidae OR sirenia OR lemmus OR perdix OR glires OR lepidosaurs OR muskox OR deinagkistrodon OR pholidota OR holocephali OR cercopithecinae OR clariidae OR agapornis OR doryteuthis OR tyrannidae OR dicroglossidae OR godwit OR godwits OR molecula OR ponginae OR atheriniformes OR colobinae OR lophocebus OR atelidae OR cottidae OR leucopsis OR acanthuridae OR didelphimorphia OR elver OR elvers OR lapponica OR dermaptera OR "european hake" OR "european hakes" OR gerbillinae OR banteng OR hartebeest OR hartebeest OR hogget OR haematopus OR "anguis fragilis" OR "grey heron" OR "grey herons" OR "blue whiting" OR "blue writings" OR furnariidae OR macrovipera OR esocidae OR lapwing OR lapwings OR mylopharyngodon OR wallabia OR beloniformes OR potoroo OR potorous OR "athene noctua" OR pleuronectidae OR bushbabies OR muscicapidae OR alligatoridae OR fuligula OR "bush baby" OR guineafowl OR spoonbill OR spoonbills OR viverridae OR catostomidae OR zebrafish OR indexes OR vendace OR estrildidae OR monotremata OR spizella OR ambystomatidae OR shelduck OR shelduck OR treeshrewdb OR treeshrewdb OR hoplobatrachus OR pochard OR hoolock OR hoolock OR lynxes OR antelope OR antelopes OR blackback OR blackbuck OR cricetidae OR paramisgurnus OR skylark OR skylarks OR soleidae OR allobates OR "northern wheatear" OR "northern wheatears" OR pitheciidae OR takin OR theria OR vanellus OR galaxiidae OR loriinae OR ostralegus OR palaeognathae OR "stone loach" OR alauda OR callitrichidae OR california OR duttaphrynus OR ictaluridae OR osteoglossiformes OR poultices OR curema OR "ruddy turnstone" OR "ruddy turnstones" OR shellfish OR sunfishes OR centropomidae OR haemachates OR platalea OR thamnophilidae OR "song thrush" OR atherinopsidae OR siluridae OR tadorna OR chroicocephalus OR ermine OR erminea OR gavialis OR ruffe OR tiphiidae OR diprotodontid OR hyaenidae OR antilopine OR crocodyloidea OR herpestidae OR hippopotamidae OR "northern shoveler" OR "round gobies" OR cheirogaleids OR indridae OR fundulids OR pythonique OR rhynchocephalia OR anodorhynchus OR "red-backed shrike" OR "red-backed shrikes" OR triozidae OR phalangeroidea OR aoudads OR boreoeutherian OR "eurasian jay" OR "eurasian jays" OR filiformis OR haplorhine OR osteoglossidae OR paenungulate OR struthioniforms OR ferina OR sanderling OR sanderlings OR spheniscidae OR cuttlefish OR cygnea OR dasycnema OR gadwall OR gadwall OR "pelobates fuscus" OR wryneck OR wryneck OR afrosoricids OR culaea OR "dover sole" OR "dover soles" OR paralichthyidae OR passerinae OR osteolaus OR "song thrushes" OR bluethroat OR bluethroat OR hydrophilidae OR megrim OR mephitinae OR strepsirhine OR toxostoma OR epidalea OR osmeriformes OR "bush babies" OR tarsiiforms OR atelidae OR bufotes OR "eurasian coot" OR "eurasian coots" OR galaginae OR geovelia OR philomachus OR tubilidentata OR bombinatoridae OR pelobatidae OR trachysaurus OR aneuridae OR woodlark OR woodlark OR alcelaphine OR redshank OR redshanks OR sapientia OR "sand smelt" OR "sand smelts" OR woodlice OR woodhouse OR dasyproctidae OR "eurasian wigeon" OR "eurasian wigeons" OR garganta OR garganey OR "lemon sole" OR "lemon soles" OR "common dab" OR "common dabs" OR greylag OR greylags OR leucopodia OR osphronemidae OR bewickii OR "common moorhen" OR "common moorhens" OR decapodiforme OR gobbled OR gobbler OR odontiphoridae OR paddlefish OR salmoninae OR esociforms OR "eurasian woodcock" OR "eurasian woodcocks" OR "european smelt" OR "european smelts" OR goldfisher OR trenches OR tyranny OR "common chaffinch" OR "common chaffinchs" OR "common redstart" OR "common redstarts" OR "common roach" OR "common roachs" OR "great knot" OR "great knots" OR potoroinae OR alydidae OR coregoninae OR dipteran OR leverett OR "poeciliopsis gracilis" OR amphiuridae OR batrachoidiform OR "bighead goby" OR heteropneusticlae OR luapula OR "norway pout" OR "norway pouts" OR sipunculids OR dogfights OR sebastinae OR taeniidae OR alethinophidian OR "common nase" OR "common nases" OR "common sandpiper" OR "common sandpipers" OR "eurasian blackcap" OR "eurasian blackcaps" OR petrochemia OR syngnathiformes OR "common chaffinches" OR eupelmidae OR octopediformis OR phascolarctids OR scophthalmidae OR "starry smooth-hound" OR "starry smooth-hounds" OR whitefish OR cuniculina OR "european sprat" OR "european sprats" OR "rosy bitterling" OR "rosy bitterlings" OR "common dace" OR "common daces" OR "lesser weever" OR "lesser weevers" OR scalefish OR "water rail" OR "water rails" OR alouattidae OR centrarchiform OR "common whitethroat" OR "common whitethroats" OR gavialinae OR "grey gurnard" OR "greygurnards" OR lateolabracidae OR rheiforme OR "tub gurnard" OR "tub gurnards" OR "common chiffchaff" OR "common chiffchaffs" OR garnishes OR "lesser whitethroat" OR "lesser whitethroats" OR myoxide OR seabastes OR spariformis OR umbridge OR "yellow boxfish" OR anabantiforms OR aoridae OR "common bleak" OR "common bleaks" OR "common rudd" OR "common rudds" OR "greater pipefish" OR happle OR nandinoideae OR "stone loaches" OR whinchat OR whinchat OR acanthodiformes OR "brotula barbata" OR "common ling" OR "common lings" OR "common roaches" OR cottonian OR cottonrat OR douroucouli OR dromadidae OR finches OR fitchen OR galaxiformes OR latrine OR saimiriine OR sornette OR tarski OR "tompot blenny" OR "common dragonet" OR "common dragonets" OR "longspinedbullhead" OR "longspined bullheads" OR monotremata OR monotremate OR pempheriformes OR perdicidae OR presbytina OR smegmamorphs OR "bighead gobies" OR "carangaria incertae sedis" OR conidae OR "fivebeard rockling" OR foulart OR foucart OR grassweed OR "greater pipefishes" OR ibises OR millionwith OR mugiliformes OR "norwegian topknot" OR prewit OR "red sea sailfin tang" OR rupicaprae OR shellfishes OR "tompot blennies" OR "twait shad" OR "yellow boxfishes")

AND

**ALL=(stroke) OR ALL=(ischemia)**

AND

ALL=(brain) OR ALL=(cerebrovascular) OR ALL=(MCA) OR ALL=(middle cerebral artery) OR ALL=(ACA) OR ALL=(anterior cerebral artery) OR ALL=(MCAO)

AND

ALL=(hydrogen sulfide) OR ALL=(hydrogen sulphide) OR ALL=(H2S) OR ALL=(thiosulfates) OR ALL=(thiosulfate) OR ALL=(thiosulphate) OR ALL=(NaHS) OR ALL=(Sodium hydrosulfide) OR ALL=(Na2S) OR ALL=(Sodium sulfide) OR ALL=(Thioglycine) OR ALL=(H2S donor) OR ALL=(H2S-releasing) OR ALL=(H2S-generating) OR ALL=(H2S donating) OR ALL=(sulfide donor) OR ALL=(sulphide donor)

**5. Scopus Search Criteria**

(INDEXTERMS ( "animal experimentation" ) OR INDEXTERMS ( "models, animal" ) OR INDEXTERMS ( invertebrates ) OR INDEXTERMS ( animals ) OR INDEXTERMS ( "animal population groups" ) OR INDEXTERMS ( chordata ) OR INDEXTERMS ( "chordata, nonvertebrate" ) OR INDEXTERMS ( vertebrates ) OR INDEXTERMS ( amphibians ) OR INDEXTERMS ( birds ) OR INDEXTERMS ( fishes ) OR INDEXTERMS ( reptiles ) OR INDEXTERMS ( mammals ) OR INDEXTERMS ( primates ) OR INDEXTERMS ( artiodactyla ) OR INDEXTERMS ( carnivora ) OR INDEXTERMS ( cetacea ) OR INDEXTERMS ( chiroptera ) OR INDEXTERMS ( elephants ) OR INDEXTERMS ( hyraxes ) OR INDEXTERMS ( insectivora ) OR INDEXTERMS ( lagomorpha ) OR INDEXTERMS ( marsupialia ) OR INDEXTERMS ( monotremata ) OR INDEXTERMS ( perissodactyla ) OR INDEXTERMS ( rodentia ) OR INDEXTERMS ( scandentia ) OR INDEXTERMS ( sirenia ) OR INDEXTERMS ( haplorhini ) OR INDEXTERMS ( strepsirhini ) OR INDEXTERMS ( platyrrhini ) OR INDEXTERMS ( tarsii ) OR INDEXTERMS ( catarrhini ) OR INDEXTERMS ( cercopithecidae ) OR INDEXTERMS ( hylobatidae ) OR INDEXTERMS ( hominidae ) OR INDEXTERMS ( "gorilla gorilla" ) OR INDEXTERMS ( "pan paniscus" ) OR INDEXTERMS ( "pan troglodytes" ) OR INDEXTERMS ( "pongo pygmaeus" ) OR INDEXTERMS ( rat ) OR INDEXTERMS ( rats ) OR INDEXTERMS ( mouse ) OR INDEXTERMS ( mice ) OR INDEXTERMS ( rabbit ) OR INDEXTERMS ( rabbits ) OR INDEXTERMS ( in AND vivo ) OR INDEXTERMS ( rodent ) OR INDEXTERMS ( rodents ) OR INDEXTERMS ( pig ) OR INDEXTERMS ( pigs )) **AND** (INDEXTERMS ( stroke ) OR INDEXTERMS ( stroke ) OR INDEXTERMS ( ischemia ) OR INDEXTERMS ( ischemia ) ) OR ( TITLE-ABS ( stroke ) OR TITLE-ABS ( ischemia )) **AND** (TITLE-ABS(brain) OR TITLE-ABS(cerebrovascular) OR TITLE-ABS("middle cerebral artery") OR TITLE-ABS(MCA) OR TITLE-ABS(ACA) OR TITLE-ABS("anterior cerebral artery") OR TITLE-ABS(MCAO)) **AND** (INDEXTERMS("hydrogen sulfide") OR TITLE-ABS("hydrogen sulfide") OR TITLE-ABS("hydrogen sulphide") OR TITLE-ABS(H2S) OR INDEXTERMS(thiosulfates) OR TITLE-ABS(thiosulfate) OR TITLE-ABS(thiosulphate) OR TITLE-ABS(NaHS) OR TITLE-ABS("Sodium hydrosulfide") OR TITLE-ABS(Na2S) OR TITLE-ABS("Sodium sulfide") OR TITLE-ABS(Thioglycine) OR TITLE-ABS("H2S donor") OR TITLE-ABS(H2S-releasing) OR TITLE-ABS(H2S-generating) OR TITLE
