# Supplementary material for: A meta-analysis of animal studies evaluating the effect of hydrogen sulfide on ischemic stroke: is the preclinical evidence sufficient to move forward?
Source: Naunyn Schmiedebergs Arch Pharmacol. 2024 Jul 17;397(12):9533–48. doi: 10.1007/s00210-024-03291-5 (PMC11582254; doi:10.1007/s00210-024-03291-5)
Supplement: Supplementary file 4 — Supplement 4. R codes (PDF 143 kb) [file 210_2024_3291_MOESM4_ESM.pdf]

Final R Scripts

Selda Emre Aydıngöz

2023-12-23

study title: “A Meta-Analysis of Animal Studies Evaluating the Effect of Hydrogen Sulfide on Ischemic Stroke Models”

protokol: “PROSPERO CRD42023380938”

```
# Load the required R packages: meta, dmetar, tidyverse.
library(meta)
library(dmetar)
library(metafor)
library(tidyverse)
library(readxl)

#1 Infarct Volume
  ##NMD pooling, forest plot, influence analysis, meta-regression, funnel plot
#2 Neurological Deficit Score
  ##NMD pooling, forest plot
#3 Brain Water Content
  ##NMD pooling, forest plot
#4 TUNEL
  ##SMD pooling, forest plot
#5 TNF-alpha
  ##SMD pooling, forest plot
#6 Caspase 3
  ##SMD pooling, forest plot
#7 IL-1beta
  ##SMD pooling, forest plot

# 1 Infarct Volume
infarctVol <- read_excel("Supplement2_Dataset.xlsx",
                         sheet = "Infarct_All")
#save the group means to a variable called mCi
mCi1 <- infarctVol$m1i

# save the group standard deviations to a variable called sdCi
sdCi1 <- infarctVol$sd1i

# save the number of animals to a variable called nCi
nCi1 <- infarctVol$n1i

# save the group means to a variable called mTi
mTi1 <- infarctVol$m2i

# save the group standard deviations to a variable called sdTi
sdTi1 <- infarctVol$sd2i

# save the number of animals to a variable called nTi
nTi1 <- infarctVol$n2i

# calculate the NMD effect size for each experiment and save the results to the
ESi variable
ESi1 <- (mCi1 - mTi1) / mCi1 * 100

# add the effect size data as a new column
infarctVol <- cbind(infarctVol, ESi1)

# control group
```

```
nsdCi1 <- sdCi1 / mCi1 * 100

# treatment group
nsdTi1 <- sdTi1 / mCi1 * 100

# calculate the NMD standard error
SEi1 <- sqrt((nsdCi1^2) / nCi1 + (nsdTi1^2) / nTi1)

# add the standard error data as a new column in the table
infarctVol <- cbind(infarctVol, SEi1)

infarctVolMA <- metagen(
  # specify the variable that contains the effect size for each experiment
  ESi1,

  # specify the variable that contains the standard error for each experiment
  SEi1,

  # specify the data set
  data = infarctVol,

  # specify the study labels
  studlab = Authors,

  # specify a random effects model
  comb.random = TRUE,

  # specify a fixed effect model
  comb.fixed = TRUE,

  # specify which method is used to estimate the between-study variance, τ^2: re
stricted maximum-likelihood (REML) estimator
  method.tau = "REML",

  # Define the sample sizes for treatment and control groups
  n.e = infarctVol$n2i,  n.c = infarctVol$n1i)

summary (infarctVolMA)

png(file = "forestplot_infarct_area_All.png", width = 8500, height = 11500, res
= 800)
infarctVolFor <- forest.meta(
  # specify the meta-analysis to plot
  infarctVolMA, sortvar = TE, leftcols = c("studlab", "n.e", "n.c"),
  leftlabs = c("Study", "n (H2S)","n (Control)"), just = "center",
  comb.random = TRUE,  common = FALSE, digits = 1, digits.pval = 4,
  digits.pval.Q = 4, digits.TE = 1, print.Q = TRUE, print.I2.ci = TRUE,
  print.pval.Q = TRUE, xlab = "NMD (% reduction in infarct volume)",
  rightlabs = c("NMD", "95% CI", "Weight"), test.overall.random = TRUE,
  addrows.below.overall = 4, col.diamond.random ="red",
  col.diamond.lines.random = "black",
  col.square = "grey",
```

```
  col.square.lines = "black")
dev.off()

###########Influence analyses
# Use the influence function (metainf) to obtain influence statistics.
meta_info1c <- metainf(infarctVolMA, pooled = "random")
print(meta_info1c)

###########Meta-regression
## 1. Species
# check the different levels of the variable and number of experiments in each
table(infarctVol$Species)

infarctVolSpecies <- metareg(
  # specify the meta-analysis
  infarctVolMA,

  # specify the experimental variable of interest
  ~ Species)

infarctVolSpecies

# view the meta-regression
infarctVolMA.Species <- update.meta(infarctVolMA,
                                    subgroup = Species,
                                    tau.common = TRUE)
infarctVolMA.Species$pval.random.w
infarctVolMA.Species

# 2. Blinded Outcome Assessment
# check the different levels of the variable and number of experiments in each
table(infarctVol$Blinded_Outcome_Assessment)
infarctVolBlind <- metareg(
  # specify the meta-analysis
  infarctVolMA,

  # specify the experimental variable of interest
  ~ Blinded_Outcome_Assessment)

infarctVolBlind

# view the meta-regression
infarctVolMA.Blind <- update.meta(infarctVolMA,
                                  subgroup = Blinded_Outcome_Assessment,
                                  tau.common = TRUE)
infarctVolMA.Blind$pval.random.w
infarctVolMA.Blind

## 3. Random outcome assesment
# check the different levels of the variable and number of experiments in each
table(infarctVol$Random_outcome_assesment)
```

```
infarctVolRandom <- metareg(
  # specify the meta-analysis
  infarctVolMA,

  # specify the experimental variable of interest
  ~ Random_outcome_assesment)

infarctVolRandom
# view the meta-regression
infarctVolMA.Random <- update.meta(infarctVolMA,
                                   subgroup = Random_outcome_assesment,
                                   tau.common = TRUE)
infarctVolMA.Random$pval.random.w
infarctVolMA.Random

## 4. Application time
# check the different levels of the variable and number of experiments in each
table(infarctVol$H2S_application_time)
infarctVolApplication <- metareg(
  # specify the meta-analysis
  infarctVolMA,

  # specify the experimental variable of interest
  ~ H2S_application_time)

infarctVolApplication
# view the meta-regression
infarctVolMA.Application <- update.meta(infarctVolMA,
                                        subgroup = H2S_application_time,
                                        tau.common = TRUE)
infarctVolMA.Application$pval.random.w
infarctVolMA.Application

## 5. Duration of Ischemia
# check the different levels of the variable and number of experiments in each
table(infarctVol$Duration_of_ischemia)
infarctVolDuration <- metareg(
  # specify the meta-analysis
  infarctVolMA,

  # specify the experimental variable of interest
  ~ Duration_of_ischemia)

infarctVolDuration

# view the meta-regression
infarctVolMA.Duration <- update.meta(infarctVolMA,
                                     subgroup = Duration_of_ischemia,
                                     tau.common = TRUE)
infarctVolMA.Duration$pval.random.w
infarctVolMA.Duration
```

```
# 6. H2S_Type
# check the different levels of the variable and number of experiments in each
table(infarctVol$H2S_donor_group)
infarctVolH2S_donor_group <- metareg(
  # specify the meta-analysis
  infarctVolMA,

  # specify the experimental variable of interest
  ~ H2S_donor_group)

infarctVolH2S_donor_group

# view the meta-regression
infarctVolMA.H2S_donor_group <- update.meta(infarctVolMA,
                                            subgroup = H2S_donor_group,
                                            tau.common = TRUE)
infarctVolMA.H2S_donor_group$pval.random.w
infarctVolMA.H2S_donor_group

# 7. H2S_Dose
# check the different levels of the variable and number of experiments in each
table(infarctVol$H2S_dose)
infarctVolH2S_dose <- metareg(
  # specify the meta-analysis
  infarctVolMA,

  # specify the experimental variable of interest
  ~ H2S_dose)

infarctVolH2S_dose

# view the meta-regression
infarctVolMA.H2S_dose <- update.meta(infarctVolMA,
                                     subgroup = H2S_dose,
                                     tau.common = TRUE)
infarctVolMA.H2S_dose$pval.random.w
infarctVolMA.H2S_dose

# 8. outcome_assessment_time
# check the different levels of the variable and number of experiments in each
table(infarctVol$outcome_assessment_time)
infarctVoloutcome_assessment_time <- metareg(
  # specify the meta-analysis
  infarctVolMA,

  # specify the experimental variable of interest
  ~ outcome_assessment_time)

# view the meta-regression
infarctVoloutcome_assessment_time
infarctVolMA.outcome_assessment_time <- update.meta(infarctVolMA,
                                                    subgroup = outcome_assessmen
```

```
t_time,
                                                     tau.common = TRUE)
infarctVolMA.outcome_assessment_time$pval.random.w
infarctVolMA.outcome_assessment_time

# 9. outcome_unit
# check the different levels of the variable and number of experiments in each
table(infarctVol$outcome_unit)
infarctVoloutcome_unit <- metareg(
  # specify the meta-analysis
  infarctVolMA,

  # specify the experimental variable of interest
  ~ outcome_unit)

infarctVoloutcome_unit

# view the meta-regression
infarctVolMA.outcome_unit <- update.meta(infarctVolMA,
                                         subgroup = outcome_unit,
                                         tau.common = TRUE)
infarctVolMA.outcome_unit$pval.random.w
infarctVolMA.outcome_unit

# 10. STAIRS_groups
# check the different levels of the variable and number of experiments in each
table(infarctVol$STAIRS_groups)
infarctVolSTAIRS_groups <- metareg(
  # specify the meta-analysis
  infarctVolMA,

  # specify the experimental variable of interest
  ~ STAIRS_groups)

infarctVolSTAIRS_groups

# view the meta-regression
infarctVolMA.STAIRS_groups <- update.meta(infarctVolMA,
                                          subgroup = STAIRS_groups,
                                          tau.common = TRUE)
infarctVolMA.STAIRS_groups$pval.random.w
infarctVolMA.STAIRS_groups

# 11. Randomization_Applied_allocation_sequence
# check the different levels of the variable and number of experiments in each
table(infarctVol$Randomization_Applied_allocation_sequence)
infarctVolRandomization_Applied_allocation_sequence <- metareg(
  # specify the meta-analysis
  infarctVolMA,

  # specify the experimental variable of interest
  ~ Randomization_Applied_allocation_sequence)
```

```
infarctVolRandomization_Applied_allocation_sequence
# view the meta-regression
infarctVolMA.Randomization_Applied_allocation_sequence <- update.meta(infarctVol
MA,
                                                                       subgroup =
Randomization_Applied_allocation_sequence,
                                                                       tau.common
= TRUE)
infarctVolMA.Randomization_Applied_allocation_sequence$pval.random.w
infarctVolMA.Randomization_Applied_allocation_sequence

# 12. Temperature_controlled
# check the different levels of the variable and number of experiments in each
table(infarctVol$Temperature_controlled)
infarctVolTemperature_controlled <- metareg(
  # specify the meta-analysis
  infarctVolMA,

  # specify the experimental variable of interest
  ~ Temperature_controlled)

infarctVolTemperature_controlled
# view the meta-regression
infarctVolMA.Temperature_controlled <- update.meta(infarctVolMA,
                                                   subgroup = Temperature_contro
lled,
                                                   tau.common = TRUE)
infarctVolMA.Temperature_controlled$pval.random.w
infarctVolMA.Temperature_controlled

###########Publication Bias Analysis
# infarctVolFun
png(file = "Funnel_plot.png", width = 4500, height = 4500, res = 800)

infarctVolFun <- funnel(
  # specify the meta-analysis to plot
  infarctVolMA,

  # plot the random effects estimate
  comb.random = TRUE,

  # specify the x axis label
  xlab = "NMD (% reduction in infarct volume)",

  # specify the measure plotted on the y axis, "invse" = inverse of the standard
error
  yaxis = "invse")

infarctVolFun

# Save the file to the working directory folder.
```

```
dev.off()

png(file = "Funnel_plot_Bias.png", width = 4500, height = 4500, res = 800)
infarctVolBias <- metabias(
  # specify the meta-analysis to analyse
  infarctVolMA,

  # specify the method behind the test statistic
  method.bias = "Egger",

  # plot the results
  plotit = TRUE)

infarctVolBias

# Save the file to the working directory folder.
dev.off()

infarctVolTrim <- trimfill(
  # specify the meta-analysis to plot
  infarctVolMA,

  # specify the side of the plot where missing studies are expected
  left = TRUE,

  # plot the random effects estimate
  comb.random = TRUE,

  # specify which method is used to estimate the between-study variance τ^2, res
tricted maximum-likelihood (REML) estimator
  method.tau = "REML")

# view the results of the trim-and-fill analysis
infarctVolTrim


png(file = "Funnel_trimfill.png", width = 4500, height = 4500, res = 800)
infarctVolTrimFun <- funnel(infarctVolTrim,
                            # plot the random effects estimate
                            comb.random = TRUE,

                            # specify the x axis label
                            xlab = "NMD (% reduction in infarct volume)",

                            # specify the measure plotted on the y axis, "invse"
= inverse of the standard error
                            yaxis = "se")
# Save the file to the working directory folder.
dev.off()


####################
```

```
### 2 Neurobehavioral
Longa <- read_excel("Supplement2_Dataset.xlsx",
                    sheet = "Neurological deficit score_LONG")

#save the group means to a variable called mCi
mCi2 <- Longa$m1i

# save the group standard deviations to a variable called sdCi
sdCi2 <- Longa$sd1i

# save the number of animals to a variable called nCi
nCi2 <- Longa$n1i

# save the group means to a variable called mTi
mTi2 <- Longa$m2i

# save the group standard deviations to a variable called sdTi
sdTi2 <- Longa$sd2i

# save the number of animals to a variable called nTi
nTi2 <- Longa$n2i

# calculate the NMD effect size for each experiment and save the results to the
ESi variable
ESi2 <- (mCi2 - mTi2) / mCi2 * 100

# add the effect size data as a new column
Longa <- cbind(Longa, ESi2)

# control group
nsdCi2 <- sdCi2 / mCi2 * 100

# treatment group
nsdTi2 <- sdTi2 / mCi2 * 100

# calculate the NMD standard error
SEi2 <- sqrt((nsdCi2^2) / nCi2 + (nsdTi2^2) / nTi2)

# add the standard error data as a new column in the table
Longa <- cbind(Longa, SEi2)

LongaMA <- metagen(
  # specify the variable that contains the effect size for each experiment
  ESi2,

  # specify the variable that contains the standard error for each experiment
  SEi2,

  # specify the data set
  data = Longa,

  # specify the study labels
```

```
  studlab = Authors,

  # specify a random effects model
  comb.random = TRUE,

  # specify a fixed effect model
  comb.fixed = FALSE,

  # specify which method is used to estimate the between-study variance, τ^2: re
stricted maximum-likelihood (REML) estimator
  method.tau = "REML",hakn = TRUE,
  control = list(stepadj=0.5, maxiter=1000),

  # Define the sample sizes for treatment and control groups
  n.e = Longa$n2i,  n.c = Longa$n1i)

LongaMA

png(file = "Forest Plot_Longa.png", width = 8000, height = 5000, res = 800)
LongaFor <- forest.meta(
  LongaMA, sortvar = TE, leftcols = c("studlab", "n.e", "n.c"),
  leftlabs = c("Study", "n (H2S)","n (Control)"), just = "center",
  comb.random = TRUE,  common = FALSE, digits = 1, digits.pval = 4,
  digits.pval.Q = 4, digits.TE = 1, print.Q = TRUE, print.I2.ci = TRUE,
  print.pval.Q = TRUE, xlab = "NMD (% reduction in deficit score)",
  rightlabs = c("NMD", "95% CI", "Weight"), test.overall.random = TRUE,
  addrows.below.overall = 4, col.diamond.random ="red",
  col.diamond.lines.random = "black",
  col.square = "grey",
  col.square.lines = "black")

# Save the file to working directory folder.
dev.off()

#####################

### 3 Brain Water
BrainWater <- read_excel("Supplement2_Dataset.xlsx",
                         sheet = "BrainWaterContent_Edema_BBBintg")

#save the group means to a variable called mCi
mCi2 <- BrainWater$m1i

# save the group standard deviations to a variable called sdCi
sdCi2 <- BrainWater$sd1i

# save the number of animals to a variable called nCi
nCi2 <- BrainWater$n1i

# save the group means to a variable called mTi
mTi2 <- BrainWater$m2i
```

```
# save the group standard deviations to a variable called sdTi
sdTi2 <- BrainWater$sd2i

# save the number of animals to a variable called nTi
nTi2 <- BrainWater$n2i

# calculate the NMD effect size for each experiment and save the results to the
ESi variable
ESi2 <- (mCi2 - mTi2) / mCi2 * 100

# add the effect size data as a new column
BrainWater <- cbind(BrainWater, ESi2)

# control group
nsdCi2 <- sdCi2 / mCi2 * 100

# treatment group
nsdTi2 <- sdTi2 / mCi2 * 100

# calculate the NMD standard error
SEi2 <- sqrt((nsdCi2^2) / nCi2 + (nsdTi2^2) / nTi2)

# add the standard error data as a new column in the table
BrainWater <- cbind(BrainWater, SEi2)

BrainWaterMA <- metagen(
  # specify the variable that contains the effect size for each experiment
  ESi2,

  # specify the variable that contains the standard error for each experiment
  SEi2,

  # specify the data set
  data = BrainWater,

  # specify the study labels
  studlab = Authors,

  # specify a random effects model
  comb.random = TRUE,

  # specify a fixed effect model
  comb.fixed = FALSE,

  # specify which method is used to estimate the between-study variance, τ^2: re
stricted maximum-likelihood (REML) estimator
  method.tau = "REML",hakn = TRUE,
  control = list(stepadj=0.5, maxiter=1000),

  # Define the sample sizes for treatment and control groups
  n.e = BrainWater$n2i,  n.c = BrainWater$n1i)
```

```
BrainWaterMA

png(file = "Brain Water Content forestplot.png", width = 8500, height = 5000, re
s = 800)

BrainWaterFor <- forest.meta(BrainWaterMA, sortvar = TE, leftcols = c("studlab",
"n.e", "n.c"),
                             leftlabs = c("Study", "n (H2S)","n (Control)"), jus
t = "center",
                             comb.random = TRUE,  common = FALSE, digits = 1, di
gits.pval = 4,
                             digits.pval.Q = 4, digits.TE = 1, print.Q = TRUE, p
rint.I2.ci = TRUE,
                             print.pval.Q = TRUE, xlab = "NMD (% reduction in wa
ter content)",
                             rightlabs = c("NMD", "95% CI", "Weight"), test.over
all.random = TRUE,
                             addrows.below.overall = 4, col.diamond.random ="re
d",
                             col.diamond.lines.random = "black",
                             col.square = "grey",
                             col.square.lines = "black")

dev.off()

########################

### 4 TNF
TNF <- read_excel("Supplement2_Dataset.xlsx",
                  sheet = "TNF-alpha")

#Control Group
# save the Control group means to a variable called mean.c
mean.c4 <- TNF$m1i

# save the Control group standard deviations to a variable called sd.c
sd.c4 <- TNF$sd1i

# save the number of control animals to a variable called n.c
n.c4 <- TNF$n1i

#Treatment Group
# save the Treatment group means to a variable called mean.e
mean.e4 <- TNF$m2i

# save the treatment group standard deviations to a variable called sd.e
sd.e4 <- TNF$sd2i

# save the number of animals in the treatment group to a variable called n.e
n.e4 <- TNF$n2i

# Use metcont to pool results (random-effects model)
```

```
TNFMA <- metacont(n.e = n.e4,
                  mean.e = mean.e4,
                  sd.e = sd.e4,
                  n.c = n.c4,
                  mean.c = mean.c4,
                  sd.c = sd.c4,
                  studlab = TNF$Authors,
                  data = TNF,
                  sm = "SMD",
                  method.smd = "Hedges",
                  fixed = FALSE,
                  random = TRUE,
                  method.tau = "REML",
                  hakn = TRUE,
                  title = "TNF-alpha")

summary(TNFMA)

png(file = "TNF-alpha forestplot.png", width = 8500, height = 4000, res = 800)

TNFFor <- forest.meta(TNFMA,
                      sortvar = TE,
                      prediction = FALSE,
                      print.tau2 = TRUE,
                      comb.random = TRUE,  common = FALSE,
                      leftlabs = c("Author", "g", "SE"),
                      label.left = "Favours H2S",
                      label.right = "Favours Control",
                      digits = 1,
                      digits.se = 1,
                      digits.pval.Q = 4, digits.pval = 4,
                      print.I2.ci = TRUE,
                      print.pval.Q = TRUE, print.Q = TRUE,
                      col.diamond.random ="red",
                      col.diamond.lines.random = "black",
                      col.square = "grey",
                      col.square.lines = "black", addrows.below.overall = 4,
                      test.overall.random = TRUE)

dev.off()

##############################

### 5 TUNEL
TUNEL <- read_excel("Supplement2_Dataset.xlsx",
                    sheet = "TUNEL")

#Control Group
# save the Control group means to a variable called mean.c
mean.c5 <- TUNEL$m1i

# save the Control group standard deviations to a variable called sd.c
```

```
sd.c5 <- TUNEL$sd1i

# save the number of control animals to a variable called n.c
n.c5 <- TUNEL$n1i

#Treatment Group
# save the Treatment group means to a variable called mean.e
mean.e5 <- TUNEL$m2i

# save the treatment group standard deviations to a variable called sd.e
sd.e5 <- TUNEL$sd2i

# save the number of animals in the treatment group to a variable called n.e
n.e5 <- TUNEL$n2i

# Use metcont to pool results (random-effects model)
TUNELMA <- metacont(n.e = n.e5,
                    mean.e = mean.e5,
                    sd.e = sd.e5,
                    n.c = n.c5,
                    mean.c = mean.c5,
                    sd.c = sd.c5,
                    studlab = TUNEL$Authors,
                    data = TUNEL,
                    sm = "SMD",
                    method.smd = "Hedges",
                    fixed = FALSE,
                    random = TRUE,
                    method.tau = "REML",
                    hakn = TRUE,
                    title = "TUNEL")

summary(TUNELMA)

png(file = "TUNEL forestplot.png", width = 8500, height = 5000, res = 800)

TUNELFor <- forest.meta(TUNELMA,
                        sortvar = TE,
                        prediction = FALSE,
                        print.tau2 = TRUE,
                        comb.random = TRUE,  common = FALSE,
                        leftlabs = c("Author", "g", "SE"),
                        label.left = "Favours H2S",
                        label.right = "Favours Control",
                        digits = 1,
                        digits.se = 1,
                        digits.pval.Q = 4, digits.pval = 4,
                        print.I2.ci = TRUE,
                        print.pval.Q = TRUE, print.Q = TRUE,
                        col.diamond.random ="red",
                        col.diamond.lines.random = "black",
                        col.square = "grey",
```

```
                         col.square.lines = "black", addrows.below.overall = 4,
                         test.overall.random = TRUE)

dev.off()

#############################

### 6 Caspase-3
Caspase <- read_excel("Supplement2_Dataset.xlsx",
                      sheet = "Caspase 3")

#Control Group
# save the Control group means to a variable called mean.c
mean.c6 <- Caspase$m1i

# save the Control group standard deviations to a variable called sd.c
sd.c6 <- Caspase$sd1i

# save the number of control animals to a variable called n.c
n.c6 <- Caspase$n1i

#Treatment Group
# save the Treatment group means to a variable called mean.e
mean.e6 <- Caspase$m2i

# save the treatment group standard deviations to a variable called sd.e
sd.e6 <- Caspase$sd2i

# save the number of animals in the treatment group to a variable called n.e
n.e6 <- Caspase$n2i

# Use metcont to pool results (random-effects model)
CaspaseMA <- metacont(n.e = n.e6,
                      mean.e = mean.e6,
                      sd.e = sd.e6,
                      n.c = n.c6,
                      mean.c = mean.c6,
                      sd.c = sd.c6,
                      studlab = Caspase$Authors,
                      data = Caspase,
                      sm = "SMD",
                      method.smd = "Hedges",
                      fixed = FALSE,
                      random = TRUE,
                      method.tau = "REML",
                      hakn = TRUE,
                      title = "Caspase 3")

summary(CaspaseMA)

png(file = "Caspase 3 forestplot.png", width = 8500, height = 3500, res = 800)
```

```
CaspaseFor <- forest.meta(CaspaseMA,
                          sortvar = TE,
                          prediction = FALSE,
                          print.tau2 = TRUE,
                          comb.random = TRUE,  common = FALSE,
                          leftlabs = c("Author", "g", "SE"),
                          label.left = "Favours H2S",
                          label.right = "Favours Control",
                          digits = 1,
                          digits.se = 1,
                          digits.pval.Q = 4, digits.pval = 4,
                          print.I2.ci = TRUE,
                          print.pval.Q = TRUE, print.Q = TRUE,
                          col.diamond.random ="red",
                          col.diamond.lines.random = "black",
                          col.square = "grey",
                          col.square.lines = "black", addrows.below.overall = 4,
                          test.overall.random = TRUE)

dev.off()

#####################

### 7 IL-1beta
IL <- read_excel("Supplement2_Dataset.xlsx",
                 sheet = "IL-1beta")

#Control Group
# save the Control group means to a variable called mean.c
mean.c7 <- IL$m1i

# save the Control group standard deviations to a variable called sd.c
sd.c7 <- IL$sd1i

# save the number of control animals to a variable called n.c
n.c7 <- IL$n1i

#Treatment Group
# save the Treatment group means to a variable called mean.e
mean.e7 <- IL$m2i

# save the treatment group standard deviations to a variable called sd.e
sd.e7 <- IL$sd2i

# save the number of animals in the treatment group to a variable called n.e
n.e7 <- IL$n2i

# Use metcont to pool results (random-effects model)
ILMA <- metacont(n.e = n.e7,
                 mean.e = mean.e7,
                 sd.e = sd.e7,
                 n.c = n.c7,
```

```
                 mean.c = mean.c7,
                 sd.c = sd.c7,
                 studlab = IL$Authors,
                 data = IL,
                 sm = "SMD",
                 method.smd = "Hedges",
                 fixed = FALSE,
                 random = TRUE,
                 method.tau = "REML",
                 hakn = TRUE,
                 title = "IL-1beta")

summary(ILMA)

png(file = "IL-1beta forestplot.png", width = 8500, height = 3500, res = 800)

     ILFor <- forest.meta(ILMA,
                          sortvar = TE,
                          prediction = FALSE,
                          print.tau2 = TRUE,
                          comb.random = TRUE,  common = FALSE,
                          leftlabs = c("Author", "g", "SE"),
                          label.left = "Favours H2S",
                          label.right = "Favours Control",
                          digits = 1,
                          digits.se = 1,
                          digits.pval.Q = 4, digits.pval = 4,
                          print.I2.ci = TRUE,
                          print.pval.Q = TRUE, print.Q = TRUE,
                          col.diamond.random ="red",
                          col.diamond.lines.random = "black",
                          col.square = "grey",
                          col.square.lines = "black", addrows.below.overall = 4,
                          test.overall.random = TRUE)

dev.off()
```
