# Supplementary material for: A meta-analysis of animal studies evaluating the effect of hydrogen sulfide on ischemic stroke: is the preclinical evidence sufficient to move forward?
Source: Naunyn Schmiedebergs Arch Pharmacol. 2024 Jul 17;397(12):9533–48. doi: 10.1007/s00210-024-03291-5 (PMC11582254; doi:10.1007/s00210-024-03291-5)
Supplement: Supplementary file 5 — Supplement 5. Summary of studies (DOCX 3.75 mb) [file 210_2024_3291_MOESM5_ESM.docx]

**Table.** Basic characteristics of the included studies

| **Study** | **Species** | **Gender / Weight** | **Type of ischemic model** | **Duration of ischemia** | **Anesthetic** | **H_2_S donor** | **Dose of H_2_S donor** | **App. route** | **Timing of H_2_S administration** | **Outcome measures** |
| --- | --- | --- | --- | --- | --- | --- | --- | --- | --- | --- |
| Fan et al. (2022) | Rat | - / 250-310 g | Temporary | 120 min | Chloral hydrate | MTC | 5 mg/kg | - | Daily for 3 days before ischemia | Infarct area |
| Florian et al. (2008) | Rat | Male / 520-600 g | Temporary | 90 min | Not reported | H_2_S | 80 ppm | Inhalation | For 48 h during reperfusion | Infarct area, functional outcome, weight loss %, ED-1 positive cells in the infarcted brain, fold changes of mRNA (Casp12, Nfkb1, Grp78) at 2 week post-stroke |
| Genc et al. (2023) | Rat | Male / 240-260 g | Temporary | 60 min | Ketamine | H_2_S | 25 micromol/kg | IP | Daily for 3-13 days after reperfusion | Infarct area, functional outcome, CD34, CD5, CD11b and GFAP intensity |
| Gheibi et al. (2014) | Rat | Male / 260-300 g | Temporary | 60 min | Chloral hydrate | NaHS | 1.5 mg | IP | Begining of ischemia | Infarct area, BBB impairment/edema, brain water content, apoptosis (TUNEL assay) |
| Han et al. (2020) | Rat | Male / 220-240 g | Temporary | 90 min | Isoflurane | GYY4137 | 1 mM | ICV | Just before reperfusion | Infarct area, BBB impairment/edema, NDS, H₂S concentration, caspase-3 activity, the phosphorylation of p38 MAPK, ERK1-2 and JNK, the expression of BAX and Bcl-2 |
| Jang et al. (2014) | Rat | Male / 250-300 g | Temporary | 120 min | Not reported | NaHS | 5 mg/kg | IP | Daily for 3-12 days after reperfusion | Infarct area, functional outcome, GFAP, NeuN, BrdU and TUNEL assay, phosphorylation of AKT and ERK, level of HIF1α, VEGF and Ang-1 expression |
| Ji et al. (2016) | Mouse | Male / 30-35 g | Temporary | 120 min | Pentobarbital | H_2_S | 40 ppm | Inhalation | Daily for 7 days before ischemia | Infarct area, NDS, functional outcome, TUNEL, MDA, IL-6, TNF- α, 8-OHdG, Cleaved caspase-3/Uncleaved Caspase-3, Nuclear Nrf2, HSP70, HSP90 |
| Jiang et al. (2017) | Rat | Male / 250-270 g | Temporary | 120 min | Isoflurane | NaHS | 5,6 mg/kg | IP | Twice daily for 3 weeks before ischemia | Infarct area, NDS, LDH activity in serum, cleaved caspase-3 expression, autophagy (LC3 II/I, p62 expression) |
| Joseph et al. (2012) | Rat | Male / 520-600 g | Temporary | 90 min | Isoflurane | H_2_S | 70 ppm | Inhalation | Inhalation for 48 hours after reperfusion | Infarct area, EEG, Annexin A1 |
| Li et al. (2012) | Rat | Male / 250-280 g | Permanent | 24 hours | Chloral hydrate | NaHS | 2.8, 11.2 mg/kg | IP | 3 hours after ischemia | Infarct area, mitochondrial injury, mitochondrial SOD, mitochondrial GPx, mitochondrial MDA, neuronal bcl-2, neuronal bax, neuronal caspase 3 |
| Li et al. (2015) | Rat | Male / 180-220 g | Temporary | 120 min | Chloral hydrate | NaHS | 25 micromol/kg | IP | 10 min after MCAO | Infarct area, NDS, mortality, P2X immunreactive cell number, P2X7 receptor immunreactivity |
| Li et al. (2016) | Rat | Male / 209-251 g | Temporary | 120 min | Chloral hydrate | NaHS | 25 micromol/kg | IP | 10 min after ischemia | Functional outcome, neurological deficit, surviving neuron numbers, morphological changes, TUNEL staining (apoptotic rate), caspase 3 and caspase 9 expression, pAKT expression |
| Lin et al. (2012) | Rat | Male / 250-300 g | Temporary | 120 min | Chloral hydrate | DAS | 100, 150, 200 mg/kg | IP | Daily for 7 days before ischemia | Infarct area, NDS, TUNEL, BCL-2, Caspase-3 |
| Lin et al. (2015) | Rat | Male / 280-300 g | Temporary | 60 min | Isoflurane | Allicin | 50 mg/kg | IP | 3, 6 or 9 hours after MCAO | Infarct area, NDS, brain water content, TUNEL, Caspase-3, sphingosine kinases 2 |
| Mendonça et al. (2020) | Rat | Male / 300 g | Temporary | 90 min | Isoflurane | ATTM | 10 mg/kg | IV | Just prior to reperfusion | Infarct area, functional outcome, antioxidant enzyme capacity (SOD; catalase, lipid peroxidation), oxidative damage, proinlammatory markers (TNF-α, IL-1b, IL-6), nitric oxide products |
| Numagami et al. (1996) | Rat | Male / 250-300 g | Temporary | 60 min | Isoflurane | WG-1, OC2186, OC5186, U74006F | 0.5, 6, 9, 6 mg/kg | IP | 30 min before ischemia | Infarct area, brain water content |
| Pomierny et al. (2021) | Rat | Male / 280-320 g | Temporary | 90 min | Isoflurane | AP39 | 50 nmol/kg | IV | Daily for 7 days until 72 h before ischemia | Infarct area, NDS, growth factor receptors, neurotrophic factors/receptors, IL-1ß, IL-6, IL-10 and TNF-α, Caspase 3, H_2_S levels |
| Qu et al. (2006) | Rat | Male / 200-250 g | Permanent | 24 hours | Not reported | NaHS | 0.09, 0.018 mmol/kg | IP | 10 min before ischemia | Infarct area, H_2_S synthesis activity, rt-PCR (CBS, CSE mRNA), effect of H_2_S synthesis inhibitors on infarct volume |
| Shi et al. (2016) | Rat | - / 230-270 g | Temporary | 120 min | Chloral hydrate | Na_2_S | 2.5, 5, 10, 20, 40 mg/kg | IV | Within 15 min right after ischemia | Infarct area, NDS |
| Shui et al. (2016) | Mouse | Male / 22-25 g | Both temporary and permanent | 90 min | Chloral hydrate | NaHS | 1, 2, 4, 8, 16 mg/kg | IP | Onset of ischemia | Infarct area, NDS, LC3-I-II, p62 and Beclin-1 expression level (biomarkers of autophagy) |
| Sun et al. (2016) | Mouse | Male / 25-28 g | Temporary | 60 min | Not reported | A6 | 100 mg/kg | IP | 3 hours after reperfusion | Infarct area |
| Tao et al. (2019) | Mouse / | - | Temporary | 60 min | Isoflurane | NaHS | 10 mg/kg | IP | 24 hours before ischemia | Functional outcome, IL-1beta, TNF-α, IL-6, IL-10, IL-12 |
| Wang et al. (2014) | Mouse | Male / 25-28 g | Temporary | 60 min | Isoflurane | NaHS, ADT | 25, 50 micromol/kg, mg/kg | IP | 3 hours after reperfusion | Infarct area, BBB impairment/edema, NDS, mortality, tight junction protein expressions, MMP9, NADPH oxidase, blood H_2_S concentration, MPO activity assay, ELISA measurement of IL-10, NF-KB translocaiton, iNOS, arginase, IL-1b, NOX 1-2-4 |
| Wang et al. (2018) | Rat | Male / 250-280 g | Temporary | 60 min | Chloral hydrate | 8e | 5, 10, 20 mg/kg | IV | Daily for 3 days after reperfusion | Infarct area, brain water content, NDS, functional outcomes, TUNEL, oxidative stress evaluation, superoxide production, PI3Kγ signaling, *NOX2* modulation |
| Wei et al. (2015) | Rat | Male / 220-250 g | Temporary | 120 min | Isoflurane | H_2_S | 40, 80 ppm | Inhalation | At the beginning of reperfusion | Infarct area, functional outcome, BBB impairment/edema, NDS, Western blot (AQP4), PKC activity detection |
| Wen et al. (2018) | Rat | Male / 250-300 g | Temporary | 120 min | Chloral hydrate | NaHS | 10^-6^ mol/kg | IV | After ischemia | Infarct area, NDS, brain water content, LDH activity, MDA content, isolated organ bath experiments on MCA |
| Woo et al. (2017) | Rat | Male / 280-310 g | Temporary | 60 min | Isoflurane | NaHS | 25 micromol/kg | IV | 1 min or 30 min before reperfusion | Infarct area, BBB impairment/edema, TUNEL staining, N asetyl L aspartate concentration, NGT score (NAA + glutamate + taurine), regional cerebral blood flow |
| Yang et al. (2022) | Rat | Male / 250-320 g | Temporary | 120 min | Chloral hydrate | NaHS | 50 micromol/kg | IP | 15 min after ischemia or sham surgery | Infarct area, NDS, ELISA assay, Western blot, H&E staining, TUNEL assay. IL1b, IL18, NLRP3, procaspase 1, cleaved caspase 1, GSDMD expression |
| Yin et al. (2013) | Rat | Male / - | Temporary | 120 min | Not reported | NaHS | 50, 100 micromol/kg | IP | 20 min before reperfusion | Infarct area, NDS, HSP20, TNF-α |
| Yin et al. (2016) | Rat | Male / 280-320 g | Temporary | 120 min | Chloral hydrate | 8d | 80, 380 mg/kg | Oral gavage | Daily for 7 days until 1 day before ischemia | Infarct area, brain water content, NDS, MDA, GSH, SOD, GSH-Px (from brain tissue) |
| Yu et al. (2015) | Rat | Male / 220-250 g | Temporary | 60 min | Halothane | NaHS | 1.25, 2.5, 5 mg/kg | IP | 2 hours after the onset of ischemia | Infarct area, NDS, TUNEL, SOD, MDA, GSH-Px |
| Zhang et al. (2015) | Rat | Male / 250-300 g | Temporary | 90 min | Chloral hydrate | Allicin | 50 mg/kg | IP | Daily for 5 days 3 hours after reperfusion | Infarct area, NDS, brain water content, NeuN‑positive staining, TNF-α, MPO, Caspase-3 |
| Zhang et al. (2017) | Mouse | Male / 25-30 g | Temporary | 60 min | Isoflurane | ADT | 50 mg/kg | IP | 3 hours after reperfusion | Infarct area, rt-PCR (IL1-β, IL-6, TNF, arginase-1, iNOS, CD32, CD206, CBS, CSE), Western blot (pAMPK, AMPK, CBS) H₂S synthesizing activity |
| Zhu et al. (2017) | Mouse / | Male / 22-25 g | Temporary | 90 min | Chloral hydrate | NaHS | 1, 2, 4 mg/kg | IP | Onset of ischemia | Infarct area, autophagic vacuol accumulatin (LC3-II and p62 expression level) |

**Abbreviations:** ADT, 5-(4-methoxyphenyl)-3H-1,2-dithiole-3-thione; ATTM, ammonium tetrathiomolybdate; BBB, blood-brain barrier; BrdU, 5-bromo-2-deoxyuridine; DAS, diallyl sulfide; EEG, electroencephalogram; GFAP, glial fibrillary acidic protein; GSH-Px, glutathione peroxidase; HIF1-α, phosphohypoxia-inducible factor-1a; HSP, heat shock protein; ICV, intracerebroventricular; IP, intraperitoneal; IV, intravenous; MAPK, mitogen-activated protein kinase; MCA, middle cerebral artery; MDA, malondialdehyde; NaHS, sodium hydrosulfide; NeuN, neuron-specific nuclear protein; NDS, neurological deficit score; MTC, S-(4-fluorobenzyl)-N-(3,4,5-trimethoxybenzoyl)-L-cysteinate; Na_2_S, sodium sulfide; NBP, 3-n-butylphthalide; SOD, superoxide dismutase.

**REFERENCES**

1. Fan J, Du J, Zhang Z, Shi W, Hu B, Hu J, Xue Y, Li H, Ji W, Zhuang J, Lv P, Cheng K, Chen K. The Protective Effects of Hydrogen Sulfide New Donor Methyl S-(4-Fluorobenzyl)-N-(3,4,5-Trimethoxybenzoyl)-l-Cysteinate on the Ischemic Stroke. Molecules. 2022 Feb 25;27(5):1554.
2. Florian B, Vintilescu R, Balseanu AT, Buga AM, Grisk O, Walker LC, Kessler C, Popa-Wagner A. Long-term hypothermia reduces infarct volume in aged rats after focal ischemia. Neurosci Lett. 2008 Jun 20;438(2):180-5.
3. Genc C, Tahta A, Erdag E, Dolas I, Sahin S, Karaoz E, Aras Y, Sabanci PA. Human-derived hair follicle stem cells and hydrogen sulfide on focal cerebral ischemia model: A comparative evaluation of radiologic, neurobehavioral and immunohistochemical results. Brain Res. 2023 Jan 15;1799:148170.
4. Gheibi S, Aboutaleb N, Khaksari M, Kalalian-Moghaddam H, Vakili A, Asadi Y, Mehrjerdi FZ, Gheibi A. Hydrogen sulfide protects the brain against ischemic reperfusion injury in a transient model of focal cerebral ischemia. J Mol Neurosci. 2014;54(2):264-70.
5. Han X, Mao Z, Wang S, Xin Y, Li P, Maharjan S, Zhang B. GYY4137 protects against MCAO via p38 MAPK mediated anti-apoptotic signaling pathways in rats. Brain Res Bull. 2020 May;158:59-65.
6. Jang H, Oh MY, Kim YJ, Choi IY, Yang HS, Ryu WS, Lee SH, Yoon BW. Hydrogen sulfide treatment induces angiogenesis after cerebral ischemia. J Neurosci Res. 2014 Nov;92(11):1520-8.
7. Ji K, Xue L, Cheng J, Bai Y. Preconditioning of H2S inhalation protects against cerebral ischemia/reperfusion injury by induction of HSP70 through PI3K/Akt/Nrf2 pathway. Brain Res Bull. 2016 Mar;121:68-74.
8. Jiang WW, Huang BS, Han Y, Deng LH, Wu LX. Sodium hydrosulfide attenuates cerebral ischemia/reperfusion injury by suppressing overactivated autophagy in rats. FEBS Open Bio. 2017 Sep 21;7(11):1686-1695.
9. Joseph C, Buga AM, Vintilescu R, Balseanu AT, Moldovan M, Junker H, Walker L, Lotze M, Popa-Wagner A. Prolonged gaseous hypothermia prevents the upregulation of phagocytosis-specific protein annexin 1 and causes low-amplitude EEG activity in the aged rat brain after cerebral ischemia. J Cereb Blood Flow Metab. 2012 Aug;32(8):1632-42.
10. Li GF, Luo HK, Li LF, Zhang QZ, Xie LJ, Jiang H, Li LP, Hao N, Wang WW, Zhang JX. Dual effects of hydrogen sulphide on focal cerebral ischaemic injury via modulation of oxidative stress-induced apoptosis. Clin Exp Pharmacol Physiol. 2012 Sep;39(9):765-71.
11. Li X, Zhang J, Zhu X, Li X, Wang X, Li D. Hydrogen sulfide protects focal cerebral ischemia-reperfusion injury in rats through the PI3K/Akt signaling pathway. Int J Clin Exp Pathol. 2016; 9(6):5930-5936.
12. Li XJ, Li CK, Wei LY, Lu N, Wang GH, Zhao HG, Li DL. Hydrogen sulfide intervention in focal cerebral ischemia/reperfusion injury in rats. Neural Regen Res. 2015 Jun;10(6):932-7.
13. Lin JJ, Chang T, Cai WK, Zhang Z, Yang YX, Sun C, Li ZY, Li WX. Post-injury administration of allicin attenuates ischemic brain injury through sphingosine kinase 2: In vivo and in vitro studies. Neurochem Int. 2015 Oct;89:92-100. doi: 10.1016/j.neuint.2015.07.022.
14. Lin X, Yu S, Chen Y, Wu J, Zhao J, Zhao Y. Neuroprotective effects of diallyl sulfide against transient focal cerebral ischemia via anti-apoptosis in rats. Neurol Res. 2012 Jan;34(1):32-7.
15. Mendonça BP, Cardoso JDS, Michels M, Vieira AC, Wendhausen D, Manfredini A, Singer M, Dal-Pizzol F, Dyson A. Neuroprotective effects of ammonium tetrathiomolybdate, a slow-release sulfide donor, in a rodent model of regional stroke. Intensive Care Med Exp. 2020 Apr 9;8(1):13.
16. Numagami Y, Sato S, Ohnishi ST. Attenuation of rat ischemic brain damage by aged garlic extracts: a possible protecting mechanism as antioxidants. Neurochem Int. 1996 Aug;29(2):135-43.
17. Pomierny B, Krzyżanowska W, Jurczyk J, Skórkowska A, Strach B, Szafarz M, Przejczowska-Pomierny K, Torregrossa R, Whiteman M, Marcinkowska M, Pera J, Budziszewska B. The Slow-Releasing and Mitochondria-Targeted Hydrogen Sulfide (H2S) Delivery Molecule AP39 Induces Brain Tolerance to Ischemia. Int J Mol Sci. 2021 Jul 22;22(15):7816.
18. Qu K, Chen CP, Halliwell B, Moore PK, Wong PT. Hydrogen sulfide is a mediator of cerebral ischemic damage. Stroke. 2006 Mar;37(3):889-93.
19. Shi HQ, Zhang Y, Cheng MH, Fan BS, Tian JS, Yu JG, Chen B. Sodium Sulfide, a Hydrogen Sulfide-Releasing Molecule, Attenuates Acute Cerebral Ischemia in Rats. CNS Neurosci Ther. 2016 Jul;22(7):625-32.
20. Shui M, Liu X, Zhu Y, Wang Y. Exogenous hydrogen sulfide attenuates cerebral ischemia-reperfusion injury by inhibiting autophagy in mice. Can J Physiol Pharmacol. 2016 Nov;94(11):1187-1192.
21. Sun Y, Zhang Y, Li Y, Cheng J, Chen S, Xiao Y, Ao G. Synthesis and biological evaluation of novel hydrogen sulfide releasing nicotinic acid derivatives. Bioorg Med Chem. 2016 Nov 1;24(21):5368-5373.
22. Tao L, Yu Q, Zhao P, Yang Q, Wang B, Yang Y, Kuai J, Ding Q. Preconditioning with hydrogen sulfide ameliorates cerebral ischemia/reperfusion injury in a mouse model of transient middle cerebral artery occlusion. Chem Biol Interact. 2019 Sep 1;310:108738.
23. Wang L, Wang X, Li T, Zhang Y, Ji H. 8e Protects against Acute Cerebral Ischemia by Inhibition of PI3Kγ-Mediated Superoxide Generation in Microglia. Molecules. 2018 Oct 31;23(11):2828.
24. Wang Y, Jia J, Ao G, Hu L, Liu H, Xiao Y, Du H, Alkayed NJ, Liu CF, Cheng J. Hydrogen sulfide protects blood-brain barrier integrity following cerebral ischemia. J Neurochem. 2014 Jun;129(5):827-38.
25. Wei X, Zhang B, Cheng L, Chi M, Deng L, Pan H, Yao X, Wang G. Hydrogen sulfide induces neuroprotection against experimental stroke in rats by down-regulation of AQP4 via activating PKC. Brain Res. 2015 Oct 5;1622:292-9.
26. Wen JY, Wang M, Li YN, Jiang HH, Sun XJ, Chen ZW. Vascular Protection of Hydrogen Sulfide on Cerebral Ischemia/Reperfusion Injury in Rats. Front Neurol. 2018 Oct 19;9:779.
27. Woo CW, Kwon JI, Kim KW, Kim JK, Jeon SB, Jung SC, Choi CG, Kim ST, Kim J, Ham SJ, Shim WH, Sung YS, Ha HK, Choi Y, Woo DC. The administration of hydrogen sulphide prior to ischemic reperfusion has neuroprotective effects in an acute stroke model. PLoS One. 2017 Nov 21;12(11):e0187910.
28. Yang KL, Li WH, Liu YJ, Wei YJ, Ren YK, Mai CD, Zhang SY, Zuo Y, Sun ZZ, Li DL, Yang CH. Hydrogen Sulfide Attenuates Neuroinflammation by Inhibiting the NLRP3/Caspase-1/GSDMD Pathway in Retina or Brain Neuron following Rat Ischemia/Reperfusion. Brain Sci. 2022 Sep 15;12(9):1245.
29. Yin J, Zeng QH, Shen Q, Yang XS. [Neuroprotective mechanism of hydrogen sulfide after cerebral ischemia-reperfusion in rats]. Zhonghua Yi Xue Za Zhi. 2013 Mar 19;93(11):868-72. Chinese.
30. Yin W, Lan L, Huang Z, Ji J, Fang J, Wang X, Ji H, Peng S, Xu J, Zhang Y. Discovery of a ring-opened derivative of 3-n-butylphthalide bearing NO/H2S-donating moieties as a potential anti-ischemic stroke agent. Eur J Med Chem. 2016 Jun 10;115:369-80.
31. Yu Q, Lu Z, Tao L, Yang L, Guo Y, Yang Y, Sun X, Ding Q. ROS-Dependent Neuroprotective Effects of NaHS in Ischemia Brain Injury Involves the PARP/AIF Pathway. Cell Physiol Biochem. 2015;36(4):1539-51.
32. Zhang B, Li F, Zhao W, Li J, Li Q, Wang W. Protective effects of allicin against ischemic stroke in a rat model of middle cerebral artery occlusion. Mol Med Rep. 2015 Sep;12(3):3734-3738.
33. Zhang M, Wu X, Xu Y, He M, Yang J, Li J, Li Y, Ao G, Cheng J, Jia J. The cystathionine β-synthase/hydrogen sulfide pathway contributes to microglia-mediated neuroinflammation following cerebral ischemia. Brain Behav Immun. 2017 Nov;66:332-346.
34. Zhu Y, Shui M, Liu X, Hu W, Wang Y. Increased autophagic degradation contributes to the neuroprotection of hydrogen sulfide against cerebral ischemia/reperfusion injury. Metab Brain Dis. 2017 Oct;32(5):1449-1458.
