# Supplementary material for: A meta-analysis of animal studies evaluating the effect of hydrogen sulfide on ischemic stroke: is the preclinical evidence sufficient to move forward?
Source: Naunyn Schmiedebergs Arch Pharmacol. 2024 Jul 17;397(12):9533–48. doi: 10.1007/s00210-024-03291-5 (PMC11582254; doi:10.1007/s00210-024-03291-5)
Supplement: Supplementary file 6 — Supplement 6. Leave-one-out analysis results (DOCX 16 kb) [file 210_2024_3291_MOESM6_ESM.docx]

**Table. Leave-one-out (Influential )analysis (random effects model)**

| **Study** | **NMD** | **[95%CI]** | **I^2^** |
| --- | --- | --- | --- |
| Omitting Wang et al. 2018a | 34.6 | [28.2-40.9] | 90.0% |
| Omitting Wang et al. 2018b | 34.5 | [28.1-40.8] | 90.0% |
| Omitting Wang et al. 2018c | 34.4 | [28.0-40.7] | 90.0% |
| Omitting Woo et al. 2017a | 34.8 | [28.4-41.2] | 90.0% |
| Omitting Woo et al. 2017b | 34.4 | [28.0-40.8] | 90.0% |
| Omitting Zhang et al. 2017 | 34.1 | [27.8-40.5] | 90.0% |
| Omitting Yin et al. 2016a | 34.7 | [28.3-41.2] | 89.9% |
| Omitting Yin et al. 2016b | 33.7 | [27.5-40.0] | 89.1% |
| Omitting Li et al. 2012a | 34.9 | [28.4-41.3] | 89.9% |
| Omitting Li et al. 2012b | 35.6 | [29.6-41.7] | 88.8% |
| Omitting Shui et al. 2016a | 34.7 | [28.3-41.2] | 90.0% |
| Omitting Shui et al. 2016b | 34.4 | [28.0-40.8] | 90.0% |
| Omitting Shui et al. 2016c | 34.6 | [28.2-41.0] | 90.0% |
| Omitting Shui et al. 2016d | 34.7 | [28.3-41.1] | 90.0% |
| Omitting Shui et al. 2016 | 34.8 | [28.4-41.2] | 90.0% |
| Omitting Han et al. 2020 | 34.7 | [28.2-41.1] | 89.8% |
| Omitting Genc et al. 2023 | 34.7 | [28.4-41.1] | 90.0% |
| Omitting Yang et al. 2022 | 34.4 | [28.0-40.8] | 90.0% |
| Omitting Wei et al. 2015a | 34.1 | [27.7-40.5] | 90.0% |
| Omitting Wei et al. 2015b | 34.2 | [27.8-40.6] | 90.0% |
| Omitting Li et al. 2015 | 34.4 | [28.0-40.8] | 90.0% |
| Omitting Qu et al. 2006a | 35.5 | [29.3-41.7] | 88.6% |
| Omitting Qu et al. 2006b | 35.9 | [30.1-41.7] | 88.3% |
| Omitting Wang et al. 2014 | 34.5 | [28.2-40.9] | 90.0% |
| Omitting Gheibi et al. 2014a | 34.7 | [28.3-41.2] | 89.9% |
| Omitting Gheibi et al. 2014b | 34.1 | [27.7-40.5] | 89.7% |
| Omitting Jang et al. 2014 | 35.1 | [28.7-41.4] | 89.9% |
| Omitting Zhu et al. 2017a | 34.5 | [28.1-40.9] | 90.0% |
| Omitting Zhu et al. 2017b | 34.3 | [27.9-40.7] | 90.0% |
| Omitting Zhu et al. 2017c | 34.5 | [28.1-40.9] | 90.0% |
| Omitting Mendonça et al. 2020 | 34.2 | [27.8-40.6] | 90.0% |
| Omitting Lin et al. 2012a | 35.0 | [28.5-41.4] | 89.6% |
| Omitting Lin et al. 2012b | 34.6 | [28.2-41.1] | 90.0% |
| Omitting Lin et al. 2012c | 34.3 | [27.9-40.8] | 90.0% |
| Omitting Yin et al. 2013a | 33.7 | [27.5-40.0] | 89.5% |
| Omitting Yin et al. 2013b | 34.3 | [27.9-40.7] | 90.0% |
| Omitting Lin et al. 2015 | 35.5 | [29.2-41.7] | 89.0% |
| Omitting Ji et al. 2016 | 34.3 | [27.9-40.7] | 90.0% |
| Omitting Joseph et al. 2012 | 34.1 | [27.7-40.5] | 90.0% |
| Omitting Zhang et al. 2015 | 34.0 | [27.6-40.3] | 89.8% |
| Omitting Fan et al. 2022 | 34.0 | [27.6-40.3] | 87.6% |
| Omitting Yu et al. 2015a | 34.6 | [28.3-41.0] | 90.0% |
| Omitting Yu et al. 2015b | 34.6 | [28.2-40.9] | 90.0% |
| Omitting Yu et al. 2015c | 34.4 | [28.1-40.8] | 90.0% |
| Omitting Pomierny et al. 2021 | 33.8 | [27.5-40.0] | 89.9% |
| Omitting Jiang et al. 2017 | 34.4 | [28.1-40.8] | 90.0% |
| Omitting Shi et al. 2016a | 35.0 | [28.6-41.4] | 89.9% |
| Omitting Shi et al. 2016b | 34.9 | [28.5-41.3] | 89.9% |
| Omitting Shi et al. 2016c | 34.6 | [28.2-41.0] | 90.0% |
| Omitting Shi et al. 2016d | 34.4 | [28.0-40.9] | 90.0% |
| Omitting Shi et al. 2016e | 34.5 | [28.1-40.9] | 90.0% |
| Omitting Sun et al. 2016 | 34.4 | [28.1-40.8] | 90.0% |
| Omitting Florian et al. 2008 | 34.3 | [27.9-40.6] | 90.0% |
| Omitting Wen et al. 2018 | 34.3 | [27.9-40.7] | 90.0% |
| **Pooled estimate** | **34.5** | **[28.2-40.8]** | **89.8%** |
