# Supplementary figures and images for: A meta-analysis of animal studies evaluating the effect of hydrogen sulfide on ischemic stroke: is the preclinical evidence sufficient to move forward?
Source: Naunyn Schmiedebergs Arch Pharmacol. 2024 Jul 17;397(12):9533–48. doi: 10.1007/s00210-024-03291-5 (PMC11582254; doi:10.1007/s00210-024-03291-5)

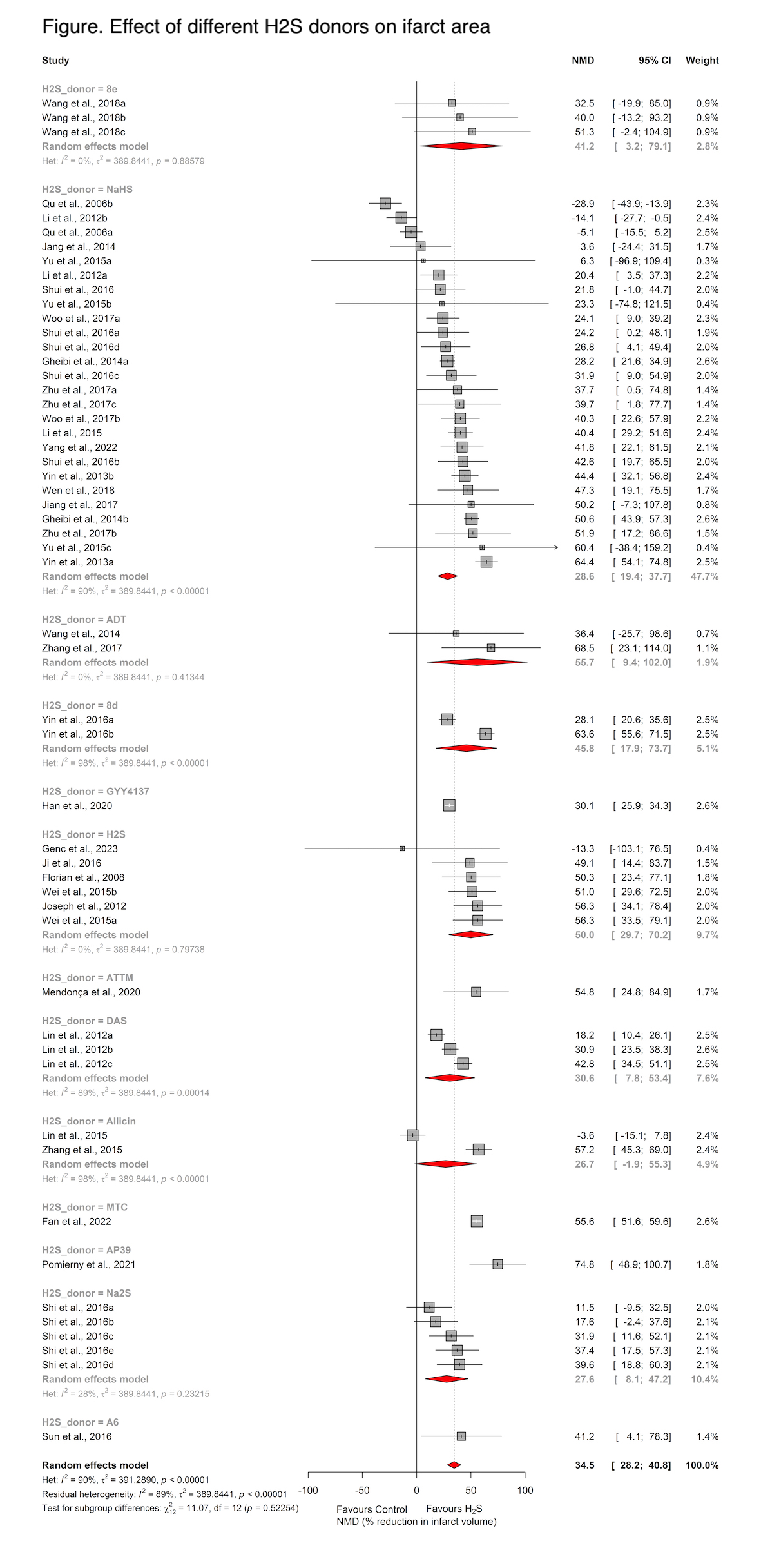

Supplement: Supplementary file 7 — Supplement 7. Forest plot showing the effect of various H2S donors on the infarct area (JPG 1117 kb) [file 210_2024_3291_MOESM7_ESM.jpg]
